# Serum Pharmacochemistry-Guided DARTS-MS Profiling Reveals Potential Mechanisms of *Caragana jubata* Against Hypoxic Pulmonary Hypertension

**DOI:** 10.3390/ijms27135815

**Published:** 2026-06-27

**Authors:** Jiacheng Hu, Yujie Qiao, Gaoxiang Lei, Xiangyun Gai, Qingqing Xia, Qiuqin Hu, Haotian Sun, Hongmai Wang, Zhanqiang Li, Yuefu Zhao, Jinyu Wang

**Affiliations:** 1 Department of Pharmacy, Qinghai Minzu University, Xining 810007, China; huxiansheng621@163.com (J.H.); 2023360859@qhmu.edu.cn (Y.Q.); leigaoxiang12@163.com (G.L.); xqing97@163.com (Q.X.); 19899704821@163.com (Q.H.); ssunht11@163.com (H.S.); wmm510921@163.com (H.W.); zhaoyuefu0208@163.com (Y.Z.); wangjinyu_0304@163.com (J.W.); 2 Plateau Medicine Research Center, Qinghai University, Xining 810001, China; zhanqiang_li@163.com

**Keywords:** *Caragana jubata* (Pall.) Poir., hypoxic pulmonary hypertension, vascular remodeling, pulmonary artery smooth muscle cells, DARTS-MS, chemoproteomics, serum pharmacochemistry

## Abstract

Hypoxic pulmonary hypertension (HPH) is a progressive vascular disease characterized by an abnormal increase in pulmonary arterial pressure resulting from pulmonary vasoconstriction and pulmonary vascular remodeling (PVR). Excessive proliferation and migration of pulmonary artery smooth muscle cells (PASMCs) are key drivers of PVR. *Caragana jubata* (Pall.) Poir. (*C. jubata*), known as “zuomaoxing” in Tibetan medicine, is traditionally used to treat blood-related disorders. However, the potential preventive and therapeutic effects of *C. jubata* on HPH remain unclear. Here, we integrated in vivo pharmacology, serum pharmacochemistry, PASMC assays, DARTS-MS chemoproteomics, and pathway validation to investigate the effects of *C. jubata* ethanol extract (ECJ) on HPH-associated PVR and the effects of a serum-exposed candidate component on CoCl_2_-induced PASMC activation. In HPH rats, ECJ reduced mean pulmonary arterial pressure and alleviated right ventricular hypertrophy and PVR. Serum pharmacochemistry detected 47 ECJ-derived serum-exposed features, including one prototype putatively annotated as ginkgolide J. Ginkgolide J attenuated CoCl_2_-induced PASMC proliferation, Ki-67 positivity, and migration without significantly affecting PASMC viability. DARTS-MS identified 1235 ginkgolide J-associated protease-susceptibility candidate proteins, and pathway validation indicated that ginkgolide J suppressed CoCl_2_-induced MEK1/ERK1/2 activation. These findings suggest that ECJ has potential value against HPH-associated PVR and that ginkgolide J is a candidate anti-proliferative compound in PASMCs.

## 1. Introduction

Hypoxic pulmonary hypertension (HPH) is a clinically important form of pulmonary hypertension characterized by progressive pulmonary vascular remodeling, increased pulmonary vascular resistance, elevated pulmonary arterial pressure, and right ventricular overload [1,2]. Chronic hypoxic exposure, including high-altitude hypoxia and hypoxia related to chronic respiratory disorders, promotes sustained pulmonary vasoconstriction and structural remodeling of small pulmonary arteries [3]. Pulmonary vascular remodeling is a central pathological feature of HPH. It involves medial wall thickening, luminal narrowing, extracellular matrix deposition, inflammatory activation, endothelial dysfunction, and abnormal behavior of vascular wall cells [4,5,6,7]. Pulmonary artery smooth muscle cells (PASMCs) play a particularly important role in this process. Under hypoxic stress, PASMCs undergo excessive proliferation, migration, phenotypic switching, and remodeling-associated signaling activation, collectively contributing to pulmonary arterial medial thickening and disease progression [8,9].

The MEK1/ERK1/2 pathway is closely involved in cellular proliferation, migration, and stress responses. Activation of MEK1 promotes phosphorylation of ERK1/2, thereby regulating downstream processes related to cell growth, survival, motility, and remodeling [10,11]. In PASMCs, MEK1/ERK1/2 signaling has been implicated in hypoxia- or growth factor-induced proliferation and migration [12,13]. Therefore, this pathway represents a biologically relevant candidate axis for investigating anti-remodeling mechanisms in hypoxia-associated pulmonary vascular disease.

*Caragana jubata* (Pall.) Poir. (*C. jubata*) is a shrubby medicinal plant distributed in high-altitude regions of China. Its heartwood is recorded as the original source of the Tibetan medicine “zuomaoxing”, which has traditionally been used to promote blood circulation and remove blood stasis for blood-related disorders [14,15]. Modern research indicates that *C. jubata* also has hypotensive, antioxidant, antithrombotic, and anti-inflammatory effects [16]. In addition, *C. jubata* has been reported to have therapeutic potential against high-altitude polycythemia (HAPC) [17,18,19]. HAPC is a chronic high-altitude-related disorder associated with an imbalance between oxygen supply and demand, excessive erythropoiesis, increased blood viscosity, increased blood flow resistance, and impaired oxygen uptake and transport, which may further contribute to pulmonary hypertension [20,21]. Both HPH and HAPC are closely associated with high-altitude hypoxia. However, whether *C. jubata* has preventive or therapeutic effects on HPH has not been clearly investigated. Moreover, as a complex herbal medicine, the ethanol extract of *C. jubata* (ECJ) contains multiple chemical constituents. A major challenge in mechanistic studies of herbal extracts is to determine which constituents are systemically exposed after oral administration and which of these candidates are functionally relevant to disease-associated cellular processes.

Serum pharmacochemistry provides a useful strategy for detecting blood-exposed chemical features of herbal medicines after in vivo administration. Compared with chemical profiling of crude extracts alone, serum pharmacochemistry prioritizes compounds or metabolic features that appear in systemic circulation and are therefore more biologically relevant for downstream pharmacological investigation. However, structural assignments based only on accurate mass, retention behavior, MS/MS fragmentation, and database matching should be interpreted cautiously when authentic standard co-elution and matched standard MS/MS confirmation are not available. Therefore, in this study, serum pharmacochemistry-derived chemical assignments were treated as putative annotations unless confirmed by reference standards.

In addition to expression-based proteomics, chemoproteomic strategies are increasingly used to identify drug–protein interactions and candidate therapeutic targets in disease-relevant models. Drug affinity responsive target stability coupled with mass spectrometry (DARTS-MS) is a label-free target-identification approach based on the principle that ligand binding can alter protein conformation and susceptibility to limited proteolysis [22,23]. Therefore, DARTS-MS does not primarily measure global protein abundance changes in response to treatment. Instead, it prioritizes proteins whose protease resistance or susceptibility changes after ligand exposure, thereby providing candidate drug-associated or drug-stabilized proteins. This feature makes DARTS-MS particularly useful for linking natural product-derived small molecules to candidate protein targets in disease-associated cellular systems.

**Figure 1:**
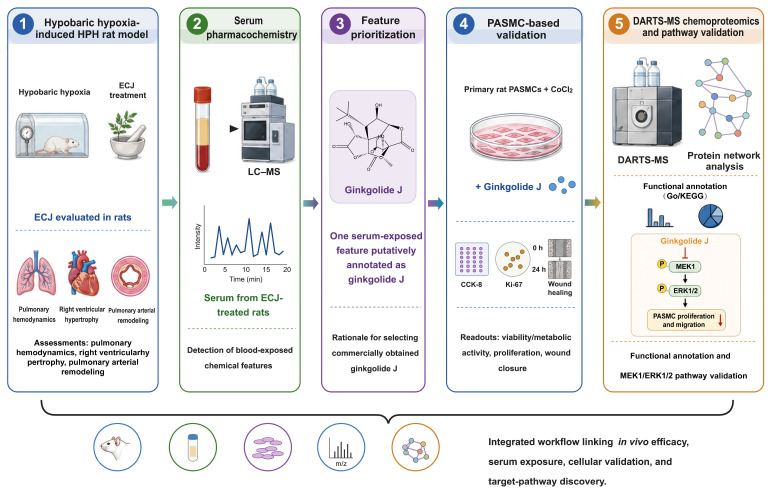
Overall study workflow. ECJ was first evaluated in a hypobaric hypoxia-induced HPH rat model to assess its effects on pulmonary hemodynamics, right ventricular hypertrophy, and pulmonary arterial remodeling. Serum pharmacochemistry was then performed using serum samples from ECJ-treated rats to detect blood-exposed chemical features. One serum-exposed feature was putatively annotated as ginkgolide J, providing the rationale for selecting ginkgolide J for PASMC-based validation. PASMCs were treated with CoCl_2_ to establish a hypoxia-mimetic cellular model, and the effects of ginkgolide J on PASMC viability/metabolic activity, proliferation, and wound closure were assessed. DARTS-MS chemoproteomics was subsequently used to identify ginkgolide J-associated protease-susceptibility candidate proteins, followed by functional annotation and MEK1/ERK1/2 pathway validation. The red downward arrow indicates decreased PASMC proliferation and migration.

In the present study, we used an integrated strategy to connect ECJ candidate compound selection, DARTS-MS-based target discovery, and PASMC proliferation mechanisms. First, ECJ was evaluated in a hypobaric hypoxia-induced rat model of HPH to assess its anti-remodeling activity and to provide serum samples for pharmacochemistry analysis. Second, serum pharmacochemistry was used to detect blood-exposed features of ECJ, including one feature putatively annotated as ginkgolide J. Third, ginkgolide J was evaluated in CoCl_2_-treated PASMCs for its effects on cell viability/metabolic activity, proliferation, and wound closure. Finally, DARTS-MS chemoproteomics was used to identify ginkgolide J-associated protease-susceptibility candidate proteins, followed by functional annotation and targeted validation of the MEK1/ERK1/2 axis. The detailed experimental procedure is shown in Figure 1.

## 2. Results

### 2.1. ECJ Reduces Mean Pulmonary Arterial Pressure (mPAP) in Hypobaric Hypoxia-Induced HPH Rats

**Figure 2:**
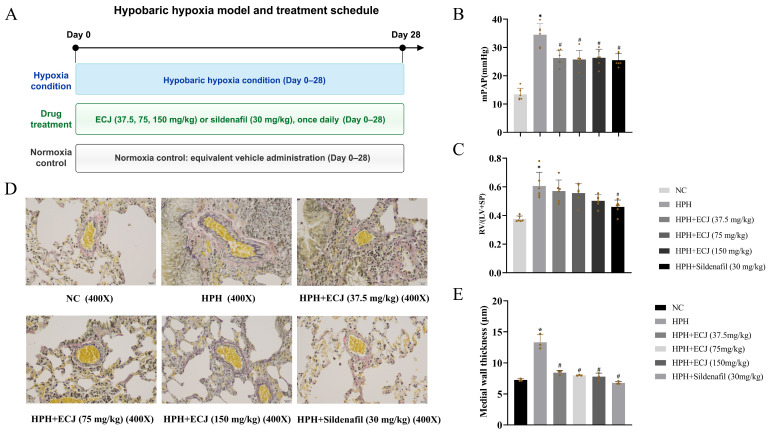
ECJ attenuates hypobaric hypoxia-induced pulmonary hypertension and pulmonary vascular remodeling in rats. (**A**) Experimental design of the hypobaric hypoxia-induced HPH model and ECJ administration. (**B**) Mean pulmonary arterial pressure (*n* = 6). * *p* < 0.05 vs. NC; ^#^ *p* < 0.05 vs. the HPH group. (**C**) Right ventricular hypertrophy index (*n* = 6). * *p* < 0.05 vs. NC; ^#^ *p* < 0.05 vs. the HPH group. (**D**) Representative EVG-stained lung sections showing pulmonary arterial remodeling. (**E**) Quantitative analysis of pulmonary arterial medial thickness (*n* = 6 rats; three vessels per rat). Scale bar = 50 μm. * *p* < 0.05 vs. NC; ^#^ *p* < 0.05 vs. the HPH group. Data are presented as mean ± SD with individual values shown as dots. Statistical significance was analyzed by one-way ANOVA followed by Dunnett’s multiple-comparison test.

To evaluate the protective effect of ECJ on HPH, rats were exposed to hypobaric hypoxia and administered ECJ or sildenafil according to the experimental schedule shown in Figure 2A. The mPAP was then measured in rats from the normoxia control (NC) group, HPH group, HPH + ECJ, and HPH + sildenafil groups (Figure 2B). Compared with the NC group, HPH exposure markedly increased mPAP, confirming the successful establishment of pulmonary hypertension (*p* < 0.05). The mPAP increased from 13.43 ± 2.19 mmHg in the NC group to 34.48 ± 3.94 mmHg in the HPH model group.

ECJ significantly reduced mPAP in HPH rats (*p* < 0.05). The mPAP values were decreased to 26.29 ± 2.71 mmHg, 25.76 ± 3.24 mmHg, and 26.33 ± 3.04 mmHg in the HPH + ECJ 37.5, 75, and 150 mg/kg groups, respectively. Compared with the HPH model group, all three ECJ doses significantly lowered mPAP (*p* < 0.05). Sildenafil, used as a positive control, also significantly reduced mPAP to 25.49 ± 2.26 mmHg compared with the HPH model group (*p* < 0.05).

These results indicate that ECJ effectively attenuates hypobaric hypoxia-induced elevation of pulmonary arterial pressure, supporting its protective effect against HPH-associated hemodynamic abnormalities.

### 2.2. ECJ Attenuates Right Ventricular Hypertrophy and Pulmonary Arterial Remodeling

Right ventricular hypertrophy was evaluated by calculating the right ventricular hypertrophy index (RVHI) (Figure 2C). Consistent with the elevation of mPAP, HPH exposure markedly increased RVHI compared with the NC group, indicating the development of right ventricular hypertrophy under hypobaric hypoxic conditions. The RVHI increased from 0.376 ± 0.021 in the NC group to 0.606 ± 0.095 in the HPH model group.

ECJ treatment reduced RVHI in HPH rats. The RVHI values were 0.571 ± 0.077, 0.557 ± 0.068, and 0.503 ± 0.042 in the HPH + ECJ 37.5, 75, and 150 mg/kg groups, respectively. Among the tested doses, ECJ at 150 mg/kg significantly reduced RVHI compared with the HPH model group (*p* < 0.05). These results suggest that high-dose ECJ alleviates HPH-induced right ventricular hypertrophy.

Pulmonary arterial remodeling was further assessed by EVG staining (Figure 2D,E). Compared with the NC group, HPH exposure significantly increased the medial thickness of pulmonary arteries from 7.25 ± 0.20 to 13.31 ± 1.22 (*p* < 0.05). ECJ significantly attenuated HPH-induced pulmonary arterial medial thickening, with medial thickness values reduced to 8.43 ± 0.36, 8.01 ± 0.07, and 7.80 ± 0.57 in the HPH + ECJ 37.5, 75, and 150 mg/kg groups, respectively, compared with the HPH model group (*p* < 0.05). Sildenafil also reduced medial thickness to 6.80 ± 0.24 compared with the HPH model group (*p* < 0.05). These findings indicate that ECJ effectively suppresses HPH-induced pulmonary arterial remodeling.

Together, these results demonstrate that ECJ alleviates both right ventricular hypertrophy and pulmonary arterial structural remodeling in HPH rats, supporting its protective effect against hypobaric hypoxia-induced pulmonary vascular remodeling.

### 2.3. Serum Pharmacochemistry Prioritizes a Serum-Exposed Feature Putatively Annotated as Ginkgolide J

**Figure 3:**
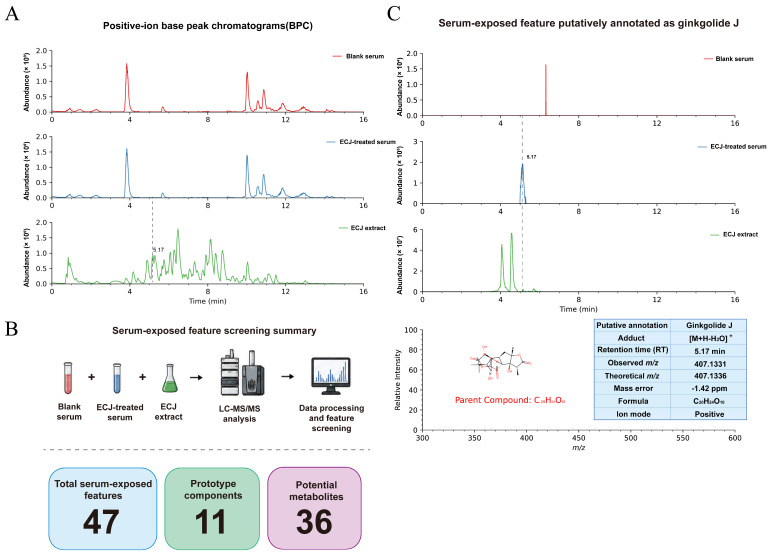
Serum pharmacochemistry prioritizes a serum-exposed feature putatively annotated as ginkgolide J. (**A**) Representative positive-ion base peak chromatograms of blank serum, ECJ-treated serum, and ECJ extract. (**B**) Summary of serum-exposed chemical features detected after ECJ administration. A total of 47 serum-exposed features were detected, including 11 prototype components and 36 potential metabolites. Among the prototype components, one serum-exposed feature putatively annotated as ginkgolide J was selected as a representative testable candidate for downstream validation. (**C**) Extracted ion chromatogram and annotation information for the serum-exposed feature putatively annotated as ginkgolide J. This feature was detected in positive ion mode at RT 5.17 min with an observed *m*/*z* of 407.1331, a theoretical *m*/*z* of 407.1336, an adduct assignment of [M + H − H_2_O]^+^, and a mass error of −1.42 ppm. Because authentic standard co-elution and matched standard MS/MS confirmation were not performed, and no informative MS/MS fragment ions were obtained, this feature was considered putatively annotated rather than unequivocally identified.

To prioritize bioavailable constituents of ECJ, serum pharmacochemistry analysis was performed by comparing blank serum, ECJ-treated serum, and ECJ extract in both positive and negative ion modes. Representative base peak chromatograms showed distinct chemical profiles among blank serum, ECJ-treated serum, and ECJ extract, supporting subsequent feature screening (Figure 3A). Additional positive- and negative-ion BPC profiles are provided in Supplementary Figure S6. Raw LC-MS data were processed using Progenesis QI, and blood-exposed features were screened according to their presence in ECJ-treated serum, comparison with blank serum, retention behavior, accurate mass, and database/reference matching.

Using this strategy, 47 serum-exposed chemical features were detected after ECJ administration, including 11 prototype components and 36 potential metabolites (Figure 3B). Prototype components were defined as features detected in both ECJ extract and ECJ-treated serum with matched retention behavior and increased or absent signals relative to blank serum, whereas potential metabolites were defined as features detected in ECJ-treated serum but not clearly detected in ECJ extract. The complete matrix, including retention time, ion mode, observed *m*/*z*, theoretical *m*/*z*, mass error, adduct type, putative formula, annotation level, and feature classification, is provided in Supplementary Table S8.

Among the 11 prototype components, one serum-exposed prototype feature was putatively annotated as ginkgolide J. This feature was detected in positive ion mode at a retention time of 5.17 min and was assigned the adduct ion [M + H − H_2_O]^+^. The observed *m*/*z* was 407.1331, and the theoretical *m*/*z* was 407.1336, corresponding to a mass error of −1.42 ppm. The molecular formula was assigned as C_20_H_24_O_10_ (Figure 3C).

Among the serum-exposed features, the prototype feature putatively annotated as ginkgolide J was prioritized as a representative candidate for downstream biological evaluation. This prioritization was based on its detection in ECJ-treated serum, acceptable accurate-mass matching within the predefined tolerance, and our preliminary analysis of serum-exposed prototype components, which suggested that ginkgolide J had marked inhibitory activity against CoCl_2_-induced PASMC proliferation. Because the serum feature was not confirmed by authentic standard co-elution and matched MS/MS, it was used as a candidate-guiding feature rather than as definitive evidence that ginkgolide J is the major or sole active constituent of ECJ.

### 2.4. Ginkgolide J Suppresses CoCl_2_-Induced PASMC Activation

To functionally examine the biological relevance of this candidate annotation, ginkgolide J was used in PASMC-based assays. The following experiments were designed to assess the anti-remodeling activity of ginkgolide J as an individual candidate compound, rather than to quantitatively determine its contribution to the overall pharmacological effect of ECJ.

**Figure 4:**
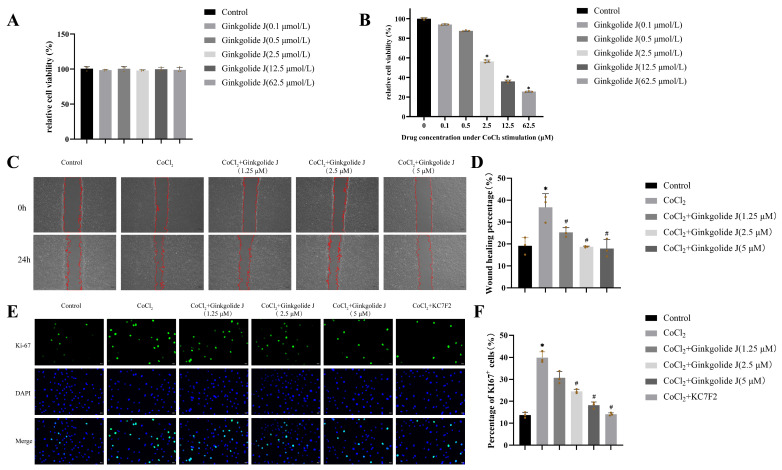
Ginkgolide J suppresses CoCl_2_-induced PASMC activation. (**A**) Cytotoxicity assessment of ginkgolide J in PASMCs over the concentration range of 0.1–62.5 μM. (**B**) Effect of ginkgolide J on the CoCl_2_-induced CCK-8 response (*n* = 6). * *p* < 0.05 vs. the control group. (**C**) Representative wound healing images obtained under serum-free conditions. Red lines indicate the wound margins used to visualize wound closure. (**D**) Quantitative analysis of wound closure (*n* = 3). * *p* < 0.05 vs. the control group; ^#^ *p* < 0.05 vs. the CoCl_2_ model group. (**E**) Representative Ki-67 immunofluorescence images. DAPI and Ki-67 channel images were sequentially acquired from the same fields and merged for presentation. For display, identical linear brightness/contrast settings were applied to the Ki-67 and DAPI channels across all groups, whereas quantification was performed on the original single-channel images. Scale bar = 50 μm. (**F**) Quantitative analysis of Ki-67-positive cells (*n* = 3). Ki-67-positive nuclei were defined as DAPI-positive nuclei with nuclear Ki-67 fluorescence intensity greater than the mean background intensity plus three standard deviations. * *p* < 0.05 vs. the control group; ^#^ *p* < 0.05 vs. the CoCl_2_ model group. Data are presented as mean ± SD. Statistical significance was analyzed by one-way ANOVA followed by Dunnett’s multiple-comparison test.

PASMCs isolated by the tissue explant method showed typical spindle-shaped morphology and positive cytoplasmic α-SMA immunocytochemical staining, supporting their smooth muscle cell identity (Figure S1). The cytotoxicity of ginkgolide J was assessed in PASMCs using the CCK-8 assay. At concentrations ranging from 0.1 to 62.5 μM, ginkgolide J did not significantly affect PASMC viability compared with the control group (*p* > 0.05; Figure 4A). Under hypoxia-mimetic conditions, CoCl_2_ stimulation significantly increased the CCK-8 signal in PASMCs compared with the control group (*p* < 0.05). Ginkgolide J effectively attenuated the CoCl_2_-induced increase in CCK-8 signal, with significant inhibition observed at 2.5–62.5 μM compared with the CoCl_2_ model group (*p* < 0.05; Figure 4B). Dose–response fitting estimated that ginkgolide J inhibited the CoCl_2_-induced CCK-8 response with an IC_50_ value of 2.145 μM.

Representative wound healing images showed that CoCl_2_ promoted wound closure, whereas ginkgolide J attenuated CoCl_2_-induced wound closure (Figure 4C). Quantitative analysis further confirmed that CoCl_2_ significantly increased the wound closure percentage compared with the control group (*p* < 0.05), while ginkgolide J at 2.5–5 μM significantly reduced the wound closure percentage compared with the CoCl_2_ model group (*p* < 0.05; Figure 4D). Ki-67 immunofluorescence further confirmed the inhibitory effect of ginkgolide J on CoCl_2_-induced PASMC proliferative activation (Figure 4E,F). Compared with the control group, CoCl_2_ significantly increased the percentage of Ki-67-positive nuclei from 13.74 ± 1.250% to 39.88 ± 2.612% (*p* < 0.05), indicating enhanced PASMC proliferation under hypoxia-mimetic stimulation. Ginkgolide J treatment reduced the percentage of Ki-67-positive nuclei to 30.74 ± 2.827%, 24.54 ± 0.922%, and 18.24 ± 1.441% at 1.25, 2.5, and 5 μM, respectively. Compared with the CoCl_2_ model group, ginkgolide J significantly decreased Ki-67 positivity at 2.5 and 5 μM (*p* < 0.05). KC7F2, used as a positive control, also significantly reduced the percentage of Ki-67-positive nuclei to 14.10 ± 0.641% compared with the CoCl_2_ model group (*p* < 0.05). Together, these results indicate that ginkgolide J suppresses CoCl_2_-induced PASMC activation, as evidenced by reduced CCK-8 response, impaired wound closure, and decreased Ki-67-positive proliferating cells.

### 2.5. DARTS-MS Identifies Ginkgolide J-Associated Protease-Susceptibility Candidate Proteins

**Figure 5:**
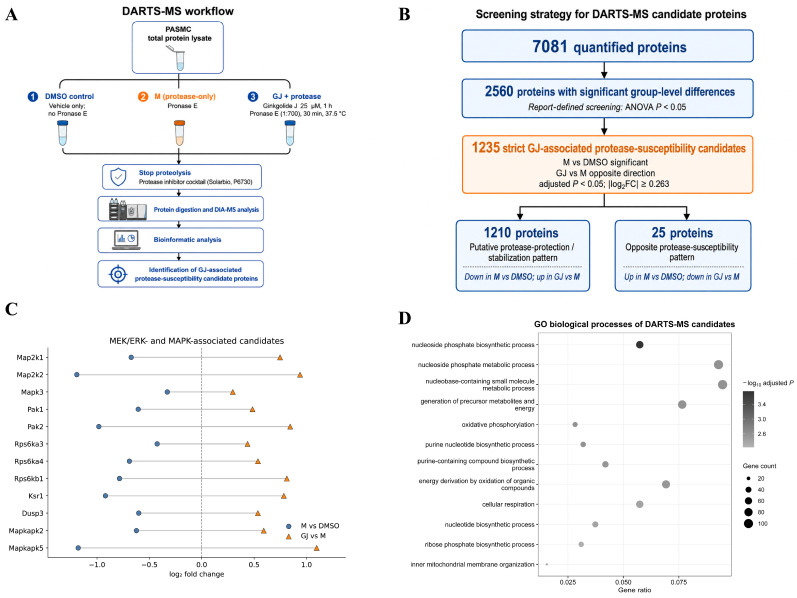
DARTS-MS-based profiling and functional annotation of ginkgolide J-associated protease-susceptibility candidate proteins in PASMC lysates. (**A**) Schematic workflow of DARTS-MS analysis. PASMC total protein lysates were divided into three groups: DMSO vehicle control without ginkgolide J or Pronase E, protease-only group, and ginkgolide J plus protease group. In the ginkgolide J plus protease group, lysates were incubated with ginkgolide J at 25 μM for 1 h and then subjected to limited proteolysis with Pronase E at an enzyme-to-protein ratio of 1:700 for 30 min at 37.5 °C. Proteolysis was terminated using a protease inhibitor cocktail before DIA-MS analysis. (**B**) Screening strategy for DARTS-MS candidate proteins. A total of 7081 proteins were quantified. The report-defined ANOVA screening identified 2560 proteins with significant group-level differences among the DMSO, protease-only, and ginkgolide J plus protease groups. Pairwise protease-susceptibility analysis further retained 1235 strict ginkgolide J-associated protease-susceptibility candidates, including 1210 proteins showing a putative protease-protection/stabilization pattern and 25 proteins showing the opposite pattern. (**C**) MEK/ERK- and MAPK-associated proteins detected among the 1235 strict ginkgolide J-associated protease-susceptibility candidates. Log_2_ fold changes are shown for the protease-only group vs. DMSO control and the ginkgolide J plus protease group vs. protease-only group comparisons. (**D**) GO biological process enrichment analysis of the 1235 strict ginkgolide J-associated protease-susceptibility candidates. All quantified DARTS-MS proteins were used as the background universe. Dot size represents gene count, and color represents −log_10_ adjusted *p* value.

To identify candidate proteins associated with ginkgolide J-mediated protease protection, a DARTS-MS workflow was performed using rat PASMC total protein lysates. Three experimental groups were included: DMSO vehicle control, protease-only group, and ginkgolide J plus protease group. In the ginkgolide J-treated group, PASMC protein lysates were incubated with ginkgolide J before limited proteolysis with Pronase E. The remaining protease-resistant protein fractions were then subjected to DIA-MS analysis (Figure 5A). This design allowed the detection of proteins whose protease susceptibility was altered in the presence of ginkgolide J, rather than conventional steady-state protein expression changes.

Quality control confirmed that the PASMC protein lysates were suitable for DARTS-MS analysis. SDS-PAGE assessment showed clear protein bands and acceptable sample integrity before mass spectrometric analysis (Figure S2). DIA-MS data were processed using DIA-NN, and protein-level quantitative matrices were used for downstream statistical analysis. Initial group-level screening identified proteins with significant abundance differences among the DMSO, protease-only, and ginkgolide J plus protease groups. Because DARTS-MS reflects changes in protease susceptibility, subsequent interpretation focused on residual protein abundance after Pronase E digestion.

A pairwise DARTS reanalysis strategy was then applied to distinguish ginkgolide J-associated protease-susceptibility changes from general protease digestion effects. Proteins were first required to show significant changes in the protease-only group compared with the DMSO control group, indicating sensitivity to Pronase E digestion. Proteins were then further filtered based on significant opposite changes in the ginkgolide J plus protease group compared with the protease-only group. Using an adjusted *p* value < 0.05 and |log_2_ fold change| ≥ 0.263 as filtering criteria, 1235 strict ginkgolide J-associated DARTS-MS candidate proteins were identified (Figure 5B). The full list of quantified proteins, ANOVA-significant proteins, strict protease-susceptibility candidates, and MAPK-associated candidates is provided in Supplementary Tables S2–S5.

Among these candidates, 1210 proteins showed a putative protease-protection/stabilization pattern, characterized by decreased residual abundance after Pronase E digestion and increased residual abundance after ginkgolide J incubation before protease treatment. This pattern is consistent with reduced protease susceptibility in the presence of ginkgolide J. In contrast, 25 proteins showed the opposite pattern, suggesting increased protease susceptibility or altered protein accessibility following ginkgolide J treatment. These findings indicate that ginkgolide J broadly reshaped the protease-susceptibility profile of PASMC proteins.

Because DARTS-MS detects ligand-associated changes in protein stability or protease accessibility, the identified proteins should be interpreted as ginkgolide J-associated protease-susceptibility candidate proteins rather than confirmed direct binding targets. Nevertheless, this unbiased target-discovery approach provided a proteome-wide candidate pool for subsequent pathway enrichment and mechanistic prioritization. The large proportion of proteins showing a putative protection pattern suggests that ginkgolide J may influence multiple protein complexes or signaling modules in PASMC lysates, thereby providing a molecular basis for further investigation of its protective effects against hypobaric hypoxia-induced pulmonary vascular remodeling.

To further connect the DARTS-MS candidate landscape with the pathway selected for downstream validation, MEK/ERK- and MAPK-associated proteins were extracted from the strict ginkgolide J-associated protease-susceptibility candidate list. Several pathway-related candidates, including Map2k1, Map2k2, Mapk3, Pak1, Pak2, Rps6ka3, Rps6ka4, Rps6kb1, Ksr1, Dusp3, Mapkapk2, and Mapkapk5, showed altered protease-susceptibility patterns in the DARTS-MS comparisons (Figure 5C). These findings supported subsequent validation of the MEK1/ERK1/2 axis in CoCl_2_-treated PASMCs, while the DARTS-MS candidates were interpreted as protease-susceptibility candidates rather than confirmed direct binding targets.

### 2.6. Functional Annotation of DARTS-MS Candidate Proteins Highlights Mitochondrial Energy Metabolism, Cytoskeletal Organization, and Vesicular Transport

Functional annotation was performed to characterize the biological context of the 1235 strict ginkgolide J-associated protease-susceptibility candidates. Using all quantified DARTS-MS proteins as the background universe, GO biological process analysis identified 54 significantly enriched terms (Figure 5D). These enriched terms were mainly associated with nucleoside phosphate biosynthesis and metabolism, nucleobase-containing small molecule metabolism, generation of precursor metabolites and energy, oxidative phosphorylation, cellular respiration, inner mitochondrial membrane organization, and respiratory electron transport.

GO cellular component analysis identified 27 significantly enriched terms, including mitochondrial inner membrane, organelle inner membrane, respiratory chain complex, oxidoreductase complex, microtubule, mitochondrial membrane, mitochondrial envelope, transmembrane transporter complex, cytosolic ribosome, proteasome regulatory particle base subcomplex, Soluble NSF Attachment Protein Receptor complex, and caveola (Figure S3). GO molecular function analysis identified 12 significantly enriched terms, including ligase activity, proton transmembrane transporter activity, structural constituent of cytoskeleton, syntaxin binding, ATP-dependent activity, proton-transporting ATP synthase activity, protein folding chaperone, proton channel activity, and Soluble NSF Attachment Protein Receptor binding (Figure S4).

KEGG pathway analysis identified 19 significantly enriched pathways, including oxidative phosphorylation, fatty acid metabolism, motor proteins, citrate cycle, fatty acid biosynthesis, synaptic vesicle cycle, carbon metabolism, phagosome, valine, leucine and isoleucine degradation, propanoate metabolism, thermogenesis, and pyruvate metabolism (Figure S5). These annotations suggest that the ginkgolide J-associated DARTS-MS candidate protein set is functionally linked to mitochondrial energy metabolism, oxidative phosphorylation, cytoskeletal organization, vesicular trafficking, and proteostasis-related processes. The complete GO and KEGG enrichment results of the strict ginkgolide J-associated protease-susceptibility candidates are provided in Supplementary Table S6.

### 2.7. Ginkgolide J Reduces Total ERK1/2 and MEK1 Expression in CoCl_2_-Stimulated PASMCs

Based on the DARTS-MS candidate protein profile and pathway prioritization, the MEK1/ERK1/2 axis was selected for further validation in CoCl_2_-stimulated PASMCs. Western blot analysis was first performed to examine the total protein expression levels of ERK1/2 and MEK1. For total ERK1/2, the combined immunoreactive ERK1/2 region was used for densitometric analysis. Compared with the control group, CoCl_2_ stimulation significantly increased the expression levels of total ERK1/2 and MEK1 (*p* < 0.05), suggesting that MEK1/ERK1/2-associated protein expression was upregulated under hypoxia-mimetic conditions.

**Figure 6:**
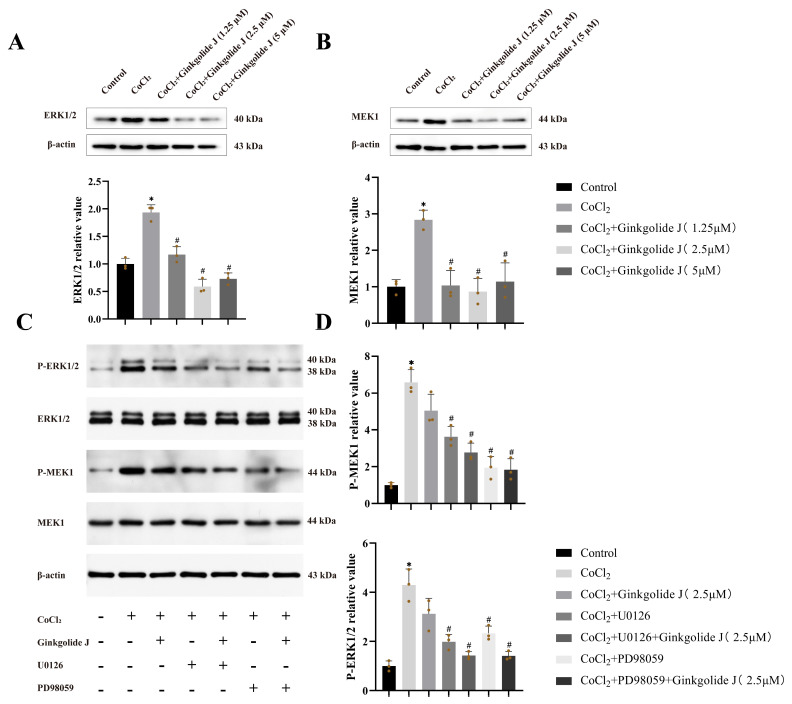
Ginkgolide J modulates MEK1/ERK1/2 signaling-associated responses in CoCl_2_-treated PASMCs. (**A**) Representative Western blot bands showing the dose-related effects of ginkgolide J on total ERK1/2 expression in CoCl_2_-treated PASMCs and quantitative analysis of total ERK1/2 expression. β-actin was used as the loading control (*n* = 3). * *p* < 0.05 vs. the control group; ^#^ *p* < 0.05 vs. the CoCl_2_ model group. (**B**) Representative Western blot bands showing the dose-related effects of ginkgolide J on total MEK1 expression in CoCl_2_-treated PASMCs and quantitative analysis of total MEK1 expression. β-actin was used as the loading control (*n* = 3). * *p* < 0.05 vs. the control group; ^#^ *p* < 0.05 vs. the CoCl_2_ model group. (**C**) Representative Western blot bands showing the effects of ginkgolide J, U0126, PD98059, and their combined treatments on p-ERK1/2, ERK1/2, p-MEK1, and MEK1 levels in CoCl_2_-treated PASMCs. (**D**) Quantitative analysis of the p-ERK1/2/ERK1/2 and p-MEK1/MEK1 ratio (*n* = 3). * *p* < 0.05 vs. the control group; ^#^ *p* < 0.05 vs. the CoCl_2_ model group. Total ERK1/2 and MEK1 were normalized to β-actin. Phosphorylated ERK1/2 and MEK1 were normalized to their corresponding total protein levels. Data are presented as mean ± SD. Statistical significance was analyzed by one-way ANOVA followed by Dunnett’s multiple-comparison test. For total ERK1/2 quantification, the total ERK1/2 immunoreactive region encompassing the closely migrating ERK1 and ERK2 isoforms was analyzed as a combined ERK1/2 signal. Full-length uncropped blot images corresponding to Figure 6 are shown in Supplementary Figure S7.

Ginkgolide J treatment significantly reduced CoCl_2_-induced total ERK1/2 expression. The relative ERK1/2 level increased from 1.00 ± 0.10 in the control group to 1.93 ± 0.14 in the CoCl_2_ model group (*p* < 0.05). Treatment with ginkgolide J decreased ERK1/2 levels to 1.17 ± 0.14, 0.59 ± 0.13, and 0.73 ± 0.10 at 1.25, 2.5, and 5 μM, respectively, compared with the CoCl_2_ model group (*p* < 0.05; Figure 6A).

A similar inhibitory pattern was observed for MEK1. CoCl_2_ significantly increased total MEK1 expression from 1.00 ± 0.20 in the control group to 2.84 ± 0.27 in the CoCl_2_ model group (*p* < 0.05). Ginkgolide J treatment significantly reduced MEK1 expression to 1.03 ± 0.41, 0.87 ± 0.36, and 1.14 ± 0.52 at 1.25, 2.5, and 5 μM, respectively, compared with the CoCl_2_ model group (*p* < 0.05; Figure 6B). These results indicate that ginkgolide J suppresses CoCl_2_-induced upregulation of total ERK1/2 and MEK1 proteins in PASMCs.

### 2.8. Western Blot Validation Supports the Involvement of the MEK1/ERK1/2 Axis

To further evaluate whether the MEK1/ERK1/2 axis was involved in the response of PASMCs to CoCl_2_ and ginkgolide J, the phosphorylation ratios of ERK1/2 and MEK1 were examined by Western blot (Figure 6C,D). Compared with the control group, CoCl_2_ stimulation significantly increased the phosphorylation ratios of both ERK1/2 and MEK1 (*p* < 0.05). The p-ERK/ERK ratio increased from 1.00 ± 0.21 in the control group to 4.30 ± 0.65 in the CoCl_2_ model group, while the p-MEK1/MEK1 ratio increased from 1.00 ± 0.14 to 6.59 ± 0.69.

Ginkgolide J at 2.5 μM significantly reduced CoCl_2_-induced phosphorylation of both ERK1/2 and MEK1 compared with the CoCl_2_ model group (*p* < 0.05). The p-ERK/ERK ratio decreased from 4.30 ± 0.65 in the CoCl_2_ model group to 3.12 ± 0.63 in the CoCl_2_ plus ginkgolide J group. Similarly, the p-MEK1/MEK1 ratio decreased from 6.59 ± 0.69 to 5.05 ± 0.88 after ginkgolide J treatment.

To further confirm the involvement of this pathway, pharmacological inhibitors were used. Compared with the CoCl_2_ model group, U0126 and PD98059 significantly reduced CoCl_2_-induced ERK1/2 phosphorylation to 1.99 ± 0.30 and 2.33 ± 0.30, respectively (*p* < 0.05). Combined treatment with ginkgolide J and U0126 or PD98059 reduced the p-ERK/ERK ratio to 1.43 ± 0.16 and 1.41 ± 0.18, respectively, compared with the CoCl_2_ model group (*p* < 0.05).

Similar inhibitory effects were observed for MEK1 phosphorylation. Compared with the CoCl_2_ model group, U0126 and PD98059 significantly reduced the p-MEK1/MEK1 ratio to 3.63 ± 0.56 and 1.95 ± 0.61, respectively (*p* < 0.05). Combined treatment with ginkgolide J and U0126 or PD98059 reduced the p-MEK1/MEK1 ratio to 2.78 ± 0.51 and 1.84 ± 0.60, respectively, compared with the CoCl_2_ model group (*p* < 0.05).

Together, these results demonstrate that CoCl_2_ activates the MEK1/ERK1/2 signaling axis in PASMCs and that ginkgolide J attenuates this activation. The inhibitor experiments further support the involvement of MEK1/ERK1/2 signaling in CoCl_2_-induced PASMC activation and in the modulatory effect of ginkgolide J.

## 3. Discussion

This study investigated the protective effects of ECJ against HPH-associated pulmonary vascular remodeling. Serum pharmacochemistry was used to prioritize serum-exposed ECJ-derived candidate components, and DARTS-MS-based chemoproteomics was subsequently applied to explore the PASMC-related mechanisms of ginkgolide J. The major findings are as follows: first, ECJ attenuated hypobaric hypoxia-induced pulmonary hemodynamic dysfunction, right ventricular hypertrophy, and pulmonary arterial remodeling in rats; second, serum pharmacochemistry detected 47 serum-exposed chemical features after ECJ administration, including 11 prototype components and 36 potential metabolites; third, one serum-exposed prototype feature was putatively annotated as ginkgolide J and was selected as a representative, testable candidate compound for downstream validation; fourth, ginkgolide J suppressed CoCl_2_-induced abnormal proliferation and migration of PASMCs; fifth, DARTS-MS identified ginkgolide J-associated protease-susceptibility candidate proteins rather than conventional expression-regulated proteins; and finally, functional annotation and pathway validation supported the involvement of mitochondrial energy metabolism-related target networks and MEK1/ERK1/2 signaling in the PASMC response to ginkgolide J.

The in vivo results support the protective effect of ECJ against hypoxia-induced pulmonary vascular remodeling. Compared with the HPH model group, ECJ significantly reduced mPAP at the tested doses (*p* < 0.05), indicating an improvement in pulmonary hemodynamic dysfunction. Although the mPAP-lowering effect did not show an obvious dose-dependent pattern across the three ECJ-treated groups, this apparent non-linear response may reflect a plateau effect within the tested dose range, nonlinear systemic exposure of active constituents, or the multi-component nature of ECJ. Pulmonary arterial pressure is an integrated hemodynamic endpoint influenced by pulmonary vasoconstriction, vascular remodeling, right ventricular adaptation, and systemic responses; therefore, it may not increase or decrease linearly with the administered extract dose. In addition to the reduction in mPAP, ECJ at 150 mg/kg significantly reduced RVHI compared with the HPH model group (*p* < 0.05), whereas lower doses showed a decreasing trend, suggesting attenuation of HPH-induced right ventricular hypertrophy. Long-term hypoxic exposure has been shown to increase pulmonary vascular resistance primarily through progressive vascular remodeling, ultimately leading to sustained elevations in pulmonary arterial pressure [24]. Consistent with previous reports, hypoxia-induced pulmonary vascular remodeling in our model was characterized by thickening of the small pulmonary artery wall, collagen deposition, and muscularization of pulmonary arterioles [25]. EVG staining further demonstrated that ECJ attenuated pulmonary arterial remodeling, as reflected by reduced pulmonary arterial medial thickness and improved vascular wall morphology. These findings suggest that ECJ may improve pulmonary arterial remodeling under chronic hypoxic conditions.

Serum pharmacochemistry was used as a candidate prioritization strategy rather than as definitive structural confirmation. For complex herbal extracts such as ECJ, compounds detected in the crude extract do not necessarily represent pharmacologically relevant constituents in vivo. Blood-exposed features therefore provide a more biologically relevant starting point for candidate component selection. In the present analysis, 47 serum-exposed features were detected, including 11 prototype components and 36 potential metabolites, suggesting that ECJ undergoes systemic exposure as a complex mixture of prototype constituents and biotransformation products rather than as a single circulating compound. The serum-exposed features were tentatively assigned to several chemical classes, including flavonoids, coumarins, phenols, anthraquinones, and phenylpropanoids, which may be relevant to the reported antioxidant and anti-inflammatory properties of ECJ. Previous studies on *C. jubata* and Lignum Caraganae have mainly focused on chemical profiling, fingerprint-based quality evaluation, compound isolation, and biological activities of extract-derived constituents [14,16,18]. However, to our knowledge, quantitative information on the abundance of ginkgolide J in *C. jubata* remains limited. Therefore, the present serum pharmacochemistry result should be viewed as a candidate-prioritization observation rather than quantitative evidence of ginkgolide J abundance in ECJ. Future targeted LC-MS/MS analysis using an authentic standard will be needed to quantify ginkgolide J in ECJ, serum, and tissue samples. Although the quantitative contribution of ginkgolide J to ECJ remains unresolved, previous studies have reported that ginkgolide J exerts anti-inflammatory activity by suppressing p38-dependent production of pro-inflammatory mediators in LPS-treated human synovial cells and protects against β-amyloid-induced synaptic dysfunction and neuronal cell death [26,27]. In addition, ginkgolides as a class have been associated with anti-inflammatory, antioxidant, neuroprotective, and MAPK/NF-κB-related regulatory activities [28,29]. For example, ginkgolide B has been shown to inhibit vascular remodeling after vascular injury by regulating the TGF-β1/Smad signaling pathway [30]. The complete serum-exposed feature matrix is provided in Supplementary Table S8.

Among the serum-exposed features, the prototype feature putatively annotated as ginkgolide J was selected as a representative candidate for downstream validation for three main reasons. First, it was annotated as a prototype ECJ-derived feature detected in ECJ-treated serum rather than as a predicted metabolite. Second, its accurate-mass match was within the predefined mass accuracy threshold, with a mass error of −1.42 ppm. Third, further screening of serum-exposed prototype components revealed that ginkgolide J exhibited a significant inhibitory effect on excessive PASMC proliferation and showed a favorable IC_50_ value. From a new drug development perspective, ginkgolide J may possess anti-remodeling potential and may therefore play a role in the treatment of pulmonary hypertension. Thus, further in-depth investigation of this compound is highly warranted. Importantly, this selection strategy does not imply that ginkgolide J is the sole, most abundant, or definitively dominant active constituent responsible for the in vivo effects of ECJ. Other serum-exposed prototype components and metabolites may also contribute to the overall pharmacological activity of ECJ and warrant further investigation.

Furthermore, the candidate compound ginkgolide J, which was screened from 11 prototype compounds, was identified based on accurate mass, retention behavior, adduct assignment, and database/reference matching, it was not unequivocally confirmed by authentic standard co-elution or matched standard MS/MS. By contrast, the PASMC functional assays and DARTS-MS experiments were performed using ginkgolide J. Therefore, the in vitro data demonstrate that ginkgolide J can suppress hypoxia-mimetic PASMC activation and modulate associated protease-susceptibility networks, but they do not prove that the serum-exposed feature is definitively ginkgolide J or that ginkgolide J quantitatively accounts for the in vivo efficacy of ECJ. This interpretation supports a conservative framework in which serum pharmacochemistry provided a candidate-prioritization clue, whereas ginkgolide J was used for functional validation.

PASMC proliferation and migration are key pathological processes in pulmonary vascular remodeling. CoCl_2_ was used to establish a hypoxia-mimetic model in PASMCs. In PASMCs, ginkgolide J did not significantly affect cell viability within the tested concentration range compared with the control group, but significantly reduced the CoCl_2_-induced increase in CCK-8 signal at 2.5–62.5 μM compared with the CoCl_2_ model group. Because CCK-8 reflects cell viability/metabolic activity and can be influenced by mitochondrial or redox changes, it was not interpreted as a stand-alone proliferation assay. The anti-proliferative interpretation was supported by Ki-67 immunofluorescence, which showed that ginkgolide J reduced the percentage of Ki-67-positive nuclei in CoCl_2_-treated PASMCs. Wound healing assays performed under serum-free conditions further showed that ginkgolide J reduced CoCl_2_-induced wound closure, suggesting reduced migratory capacity. In the context of HPH, excessive PASMC proliferation and migration are maladaptive responses that contribute to pulmonary arterial medial thickening and vascular remodeling. Therefore, suppression of CoCl_2_-induced PASMC activation is considered beneficial in this disease model. Importantly, ginkgolide J did not show obvious cytotoxicity within the tested concentration range, suggesting that its inhibitory effects on CCK-8 response, Ki-67 positivity, and wound closure reflect attenuation of pathological PASMC activation rather than nonspecific cell injury. Together, these results support the anti-remodeling activity of ginkgolide J in a PASMC-based hypoxia-mimetic model.

A key methodological feature of this study is the use of DARTS-MS as a chemoproteomic target-discovery platform. Unlike conventional expression-based proteomics, DARTS-MS does not primarily measure global changes in protein expression induced by disease or drug treatment. Instead, it identifies proteins whose susceptibility to limited proteolysis changes after ligand incubation, which may reflect direct or indirect ligand-associated stabilization or altered protein accessibility. Therefore, the DARTS-MS candidate protein list should not be interpreted as a conventional expression profile of PASMC remodeling. Rather, it provides a prioritized set of candidate ginkgolide J-associated proteins for functional annotation and downstream pathway validation.

In the present study, DARTS-MS was performed using native PASMC lysates without CoCl_2_ stimulation. Thus, the identified candidates reflect ginkgolide J-associated protease-susceptibility changes in the basal PASMC proteome, rather than a target landscape derived from CoCl_2_-activated PASMCs. The CoCl_2_-induced hypoxia-mimetic model was used in intact-cell functional assays and pathway validation to examine whether pathways prioritized from the DARTS-MS candidate landscape were functionally relevant under hypoxia-mimetic PASMC activation. This distinction should be considered when interpreting the translational relevance of the DARTS-MS candidates, particularly because CoCl_2_ can induce hypoxia-related responses but may also produce metal ion-associated off-target effects.

The DARTS-MS screening was performed using ginkgolide J at 25 μM, approximately 11.7-fold higher than the IC_50_ estimated from the CCK-8 assay. This concentration was selected as an exploratory screening concentration to improve the detection of ligand-associated protease-susceptibility changes in complex PASMC lysates. DARTS is a label-free target-identification strategy based on the principle that ligand binding can alter the protease susceptibility of target proteins, and it can be performed using purified proteins or total cell lysates, followed by proteolysis and protein detection or proteomics analysis [23,31]. Therefore, ligand concentrations in DARTS-based assays are generally optimized according to the detectability of target protection or protease-susceptibility changes in lysate systems, rather than being strictly determined by cellular IC_50_ values. DARTS-based strategies have also been applied to natural-product target identification without chemical modification or labeling of the ligand [22]. Nevertheless, the use of 25 μM may increase the possibility of detecting low-affinity or non-specific protease-susceptibility alterations. Therefore, the DARTS-MS results were interpreted as a hypothesis-generating candidate landscape rather than definitive evidence of direct target engagement at pharmacologically effective cellular concentrations.

The present DARTS-MS analysis used a three-group design consisting of DMSO control, protease-only, and ginkgolide J plus protease groups. In the initial exploratory group-level analysis, 2560 proteins showed significant differences among the DMSO control, protease-only, and ginkgolide J plus protease groups using one-way ANOVA with *p* < 0.05 as the preliminary threshold. Because DARTS-MS reflects ligand-associated changes in protease susceptibility rather than conventional changes in steady-state protein expression, we further performed pairwise protease-susceptibility reanalysis to identify proteins with a DARTS-consistent response pattern. The resulting 1235 strict ginkgolide J-associated protease-susceptibility candidates included 1210 proteins with reduced residual abundance after protease digestion and increased residual abundance after ginkgolide J incubation, consistent with a putative protection or stabilization pattern. The remaining 25 candidates showed the opposite pattern, suggesting that ginkgolide J may also alter protease susceptibility in the reverse direction for a small subset of proteins. Importantly, these proteins should be interpreted as DARTS-MS candidates rather than differentially expressed proteins.

Functional annotation of the DARTS-MS candidate target set suggested that candidate ginkgolide J-associated proteins were linked to mitochondrial energy metabolism, oxidative phosphorylation, cellular respiration, fatty acid metabolism, carbon metabolism, cytoskeletal organization, vesicular trafficking, and proteostasis-related processes. These categories are biologically relevant to PASMC remodeling because hypoxia-mimetic stress can alter mitochondrial function, bioenergetic adaptation, protein homeostasis, cytoskeletal dynamics, and intracellular trafficking. The enrichment of mitochondrial inner membrane, respiratory chain complex, oxidative phosphorylation, and proton transmembrane transporter activity suggests that ginkgolide J-associated candidate proteins may be connected to the metabolic and mitochondrial state of PASMCs. In parallel, enrichment of microtubule, Soluble NSF Attachment Protein Receptor complex, syntaxin binding, and motor proteins may be related to cytoskeletal remodeling and vesicular transport processes that participate in PASMC activation and migration.

The DARTS-MS results also provided a rationale for targeted validation of MEK1/ERK1/2 signaling. Although GO/KEGG enrichment did not indicate that ERK1/2 was the only or dominant pathway, several MEK/ERK- and MAPK-associated candidates were detected within the DARTS-MS candidate landscape, including Map2k1, Map2k2, Mapk3, Pak1, Pak2, Rps6ka3, Rps6ka4, Rps6kb1, Ksr1, Dusp3, Mapkapk2, and Mapkapk5. These findings, together with the known role of MEK1/ERK1/2 signaling in PASMC proliferation and migration, supported subsequent pathway validation. Western blot analysis showed that ginkgolide J reduced CoCl_2_-induced total ERK1/2 and MEK1 expression and attenuated the phosphorylation ratios of ERK1/2 and MEK1. Inhibitor experiments using U0126 and PD98059 further supported the involvement of the MEK/ERK cascade in CoCl_2_-induced PASMC activation. However, these findings do not prove that MEK1 or ERK1/2 is the direct molecular target of ginkgolide J. Instead, the data support a model in which modulation of MEK1/ERK1/2 signaling is one mechanism associated with the anti-remodeling response of PASMCs to ginkgolide J.

Several limitations should be acknowledged. First, serum pharmacochemistry was performed using serum from rats treated with ECJ at 150 mg/kg, which was selected to improve the detectability of circulating chemical features. Therefore, the detected serum-exposed feature putatively annotated as ginkgolide J cannot be assumed to quantitatively explain the efficacy of lower ECJ doses. Second, the serum pharmacochemistry analysis was qualitative and feature-prioritization oriented; absolute plasma concentrations, pharmacokinetic parameters, and exposure–response relationships of ginkgolide J or other serum-exposed ECJ-derived features were not determined. Third, the assignment of this serum-exposed feature as ginkgolide J was based on accurate-mass-supported putative annotation rather than authentic standard co-elution and matched standard MS/MS confirmation. Fourth, although ginkgolide J showed anti-remodeling activity in PASMCs, its relative contribution to the overall in vivo efficacy of ECJ remains unknown. Fifth, only male rats were used, and potential sex-dependent responses were not evaluated. Sixth, CoCl_2_ was used as a hypoxia-mimetic stimulus and does not fully reproduce true low-oxygen culture conditions. Seventh, the DARTS-MS assay was conducted at a single exploratory concentration of ginkgolide J, and concentration-dependent DARTS-MS or orthogonal target-engagement validation at lower concentrations would be needed to further refine target prioritization. Future studies are needed to evaluate concentration-dependent target stabilization and to strengthen target-prioritization confidence. Finally, DARTS-MS identifies candidate ligand-associated proteins based on altered protease susceptibility, but additional validation such as CETSA, purified protein binding assays, knockdown/overexpression rescue experiments, or direct target engagement assays would be needed to confirm direct targets and pathway dependency.

## 4. Materials and Methods

### 4.1. Chemicals and Reagents

Methanol, acetonitrile, and formic acid of LC-MS grade were purchased from Thermo Fisher Scientific (Waltham, MA, USA). Sodium carboxymethyl cellulose (CMC-Na) was obtained from Shanghai Yuanye Bio-Technology Co., Ltd. (Shanghai, China). Commercially obtained ginkgolide J with a purity of ≥98% was purchased from MedChemExpress (MCE, Shanghai, China; Cat# HY-N0786). CoCl_2_ was purchased from Sigma-Aldrich (St. Louis, MO, USA; Cat# 60818-50G). U0126 and PD98059 were purchased from MedChemExpress (MCE, Shanghai, China; Cat# HY-12031A and HY-12028, respectively). Cell Counting Kit-8 was purchased from Elabscience (Wuhan, China; Cat# E-CK-A362). Pronase E from *Streptomyces griseus* was purchased from Shanghai Yuanye Bio-Technology Co., Ltd. (Shanghai, China; Cat# S10014-250 mg). A protease inhibitor cocktail was purchased from Solarbio (Beijing, China; Cat# P6730).For Western blot, the following primary antibodies were used: anti-β-actin (rabbit, Proteintech, Wuhan, China; Cat# 81115-1-RR, 1:10,000), anti-phospho-ERK1/2 (rabbit, Proteintech, Wuhan, China; Cat# 28733-1-AP, 1:1000), anti-ERK1/2 (rabbit, Proteintech, Wuhan, China; Cat# 11257-1-AP, 1:2000), anti-phospho-MEK1 (rabbit, MCE, Shanghai, China; Cat# HY-P86143, 1:1000), and anti-MEK1 (mouse, Proteintech, Wuhan, China; Cat# 67872-1-Ig, 1:2000). HRP-conjugated goat anti-rabbit IgG (ASPEN, Wuhan, China; Cat# AS1107, 1:10,000) and HRP-conjugated goat anti-mouse IgG (ASPEN, Wuhan, China; Cat# AS1106, 1:10,000) were used as secondary antibodies.

### 4.2. Plant Material and Preparation of ECJ

*Caragana jubata* (Pall.) Poir. was collected from two locations in Qinghai Province, China: Daban Mountain, Datong County, Xining, and Laji Mountain, Hainan Tibetan Autonomous Prefecture. The plant material was taxonomically authenticated by Professors Pengcheng Lin and Dangwei Zhou from the School of Pharmacy, Qinghai Minzu University. A voucher specimen was deposited at the Northwest Institute of Plateau Biology, Chinese Academy of Sciences, under voucher number HNWP0334868.

The woody parts of *C. jubata* were air-dried and pulverized into a coarse powder. The powdered material was extracted with 80% ethanol using Soxhlet extraction under reflux. Briefly, the powder was soaked in 80% ethanol at a solid-to-liquid ratio of 1:3 (*w*/*v*) for 24 h and then extracted under reflux for three consecutive cycles, each lasting 2 h. The combined extracts were filtered, concentrated under reduced pressure, and evaporated to dryness to obtain ECJ. The dried extract was resuspended in 0.5% CMC-Na immediately before administration.

### 4.3. Animals and Ethics Statement

A total of 36 male Sprague-Dawley rats aged 6 to 8 weeks, weighing approximately 250 g, were purchased from Shaanxi Provincial Laboratory Animal Center, China (animal production license No. SCXK [Shaan] 2023-002). Rats were housed in a controlled environment with constant temperature and humidity, under a 12 h light/12 h dark cycle, with free access to standard food and tap water. Animals were acclimatized for 1 week prior to experimentation. All study protocols were performed in strict accordance with the institutional guidelines and the Animal Research: Reporting of In vivo Experiments (ARRIVE) guidelines 2.0. Animals were to be euthanized if they exhibited a body weight loss of ≥20%, severe lethargy, unresponsiveness, impaired mobility, or labored breathing. However, none of the animals exhibited clinical signs that met these predefined humane endpoint criteria during the study period.

The sample size was determined by power calculation based on preliminary data and the expected effect size for pulmonary arterial pressure and pulmonary arterial medial thickening. Six rats were included in each group. After acclimatization, animals were randomly assigned to experimental groups using a random number table. No animals died during the experiment, and no animals were excluded from the final analysis. After the modeling period, rats were anesthetized by an intraperitoneal injection of 20% urethane (1 g/kg) (Shanghai Macklin Biochemical Co., Ltd., Shanghai, China; Cat# U820333). Once the animals exhibited a loss of pedal and corneal reflexes, indicating a surgical plane of anesthesia, they were used for hemodynamic and hematological assessments. Death was confirmed according to the AVMA Guidelines for the Euthanasia of Animals, based on the cessation of heartbeat and respiratory movements, followed by exsanguination.

All animal procedures were performed in accordance with the National Institutes of Health Guide for the Care and Use of Laboratory Animals and the ARRIVE guidelines. The experimental protocol passed the science and technology ethics review of Qinghai Minzu University. (approval No. 2024-013; approved on 3 August 2024). All efforts were made to minimize animal suffering.

### 4.4. Hypobaric Hypoxia-Induced HPH Model and Drug Administration

After acclimatization, male Sprague-Dawley rats were randomly assigned to experimental groups, and each rat was assigned a unique identification number. The experimental groups were as follows: normoxia control group, HPH model group, HPH + ECJ 37.5 mg/kg group, HPH + ECJ 75 mg/kg group, HPH + ECJ 150 mg/kg group, and HPH + sildenafil 30 mg/kg group. Rats in the HPH and treatment groups were exposed to hypobaric hypoxia conditions (hypobaric conditions were as follows; DYC3000 chamber, Fenglei, Guizhou, China) for 28 days to establish the HPH model (Figure 2A).

Representative chamber parameters during hypobaric hypoxia exposure were as follows: chamber pressure, 49.37 kPa; oxygen partial pressure, 9.58 kPa; airflow rate, 152 m^3^/h; temperature, 19.4 °C; relative humidity, 57.0%; CO_2_ concentration, 2111 ppm; and oxygen fraction, 19.4%. Although the oxygen fraction remained close to atmospheric oxygen percentage, the reduced barometric pressure resulted in a markedly lower oxygen partial pressure.

ECJ was suspended in 0.5% CMC-Na and administered once daily by oral gavage at doses of 37.5, 75, and 150 mg/kg. Sildenafil was administered once daily at 30 mg/kg as a positive control. Rats in the normoxia and HPH model groups received an equivalent volume of 0.5% CMC-Na.

### 4.5. Hemodynamic Measurement and Right Ventricular Hypertrophy Index

After 28 days of hypoxic exposure and treatment, rats were anesthetized by intraperitoneal injection of 20% urethane (1 g/kg). A silicone catheter was inserted through the right external jugular vein and advanced into the pulmonary artery via the right atrium and right ventricle. Correct catheter positioning was confirmed by characteristic pressure waveforms. Mean pulmonary arterial pressure was continuously recorded using an MP100 pressure signal acquisition system.

After hemodynamic assessment, rats were euthanized by exsanguination under deep anesthesia. The heart was rapidly excised, and the right ventricular free wall was separated from the left ventricle plus septum (LV + S). The right ventricular hypertrophy index (RVHI) was calculated as RV/(LV + S).

### 4.6. Elastica van Gieson Staining and Morphometric Analysis

Lung tissues were collected after hemodynamic assessment, rinsed with saline, fixed in 4% paraformaldehyde, embedded in paraffin, and sectioned at 4 μm thickness. Pulmonary arterial remodeling was evaluated by Elastica van Gieson staining according to the manufacturer’s instructions.

Pulmonary arterioles with external diameters of 50–100 μm were selected for morphometric analysis. For each rat, three pulmonary arterioles were randomly selected and analyzed, resulting in 18 vessels per group. The mean value of the three vessels from each rat was used as the representative value for that animal. Histological images and morphometric measurements were analyzed by investigators blinded to group allocation.

### 4.7. Serum Pharmacochemistry Analysis

Serum pharmacochemistry was performed to characterize blood-exposed chemical features of ECJ after oral administration. Serum samples were collected from rats treated with ECJ at 150 mg/kg, which represented the highest dose within the current effective dose range and was selected to improve the detectability of circulating chemical features.

For serum sample preparation, frozen serum samples were thawed on ice. A total of 150 μL serum was mixed with 600 μL of protein precipitation solvent consisting of methanol/acetonitrile (2:1, *v*/*v*) containing 4 μg/mL mixed internal standards, including L-2-chlorophenylalanine, succinic acid-d4, and cholic acid-d4. The mixture was vortexed for 1 min, sonicated in an ice-water bath for 10 min, kept at −40 °C for 30 min, and centrifuged at 12,000 rpm for 10 min at 4 °C. A 500 μL aliquot of the supernatant was dried and reconstituted in 200 μL of water/methanol/acetonitrile (1:2:1, *v*/*v*/*v*). After vortexing, sonication, overnight storage at −40 °C, and centrifugation, 120 μL of the supernatant was transferred to an autosampler vial for LC-MS analysis.

For ECJ extract analysis, approximately 100 mg of dried ECJ powder was extracted with 1 mL of 70% methanol containing 4 μg/mL mixed internal standards. The sample was vortexed, homogenized, sonicated for 60 min in an ice-water bath, and centrifuged at 12,000 rpm for 10 min at 4 °C. The supernatant was diluted two-fold with 70% methanol containing internal standards before LC-MS analysis. Blank serum, ECJ-treated serum, and ECJ extract samples were processed with the same internal standard mixture to improve comparability.

Chromatographic separation was performed on an ACQUITY UPLC I-Class system using an ACQUITY UPLC HSS T3 column (100 mm × 2.1 mm, 1.8 μm) maintained at 45 °C. The mobile phases consisted of water containing 0.1% formic acid (A) and acetonitrile (B), with a flow rate of 0.35 mL/min and an injection volume of 5 μL. The gradient was as follows: 0–2 min, 5% B; 2–4 min, 5–30% B; 4–8 min, 30–50% B; 8–10 min, 50–80% B; 10–14 min, 80–100% B; 14–15 min, 100% B; 15–15.1 min, 100–5% B; and 15.1–16 min, 5% B. Mass spectrometric data were acquired using a high-resolution Q Exactive mass spectrometer equipped with a HESI source in both positive and negative ion modes. Full MS/dd-MS2 data were collected in a data-dependent acquisition mode, with the top eight precursor ions selected for MS/MS fragmentation after each full scan. The mass range was *m*/*z* 100–1500, the full MS resolution was 60,000, the MS/MS resolution was 15,000, and stepped collision energies of 10, 20, and 40 were used.

Raw LC-MS data were processed using Progenesis QI v3.0 for baseline filtering, smoothing, peak detection, peak integration, retention time alignment, peak alignment, and normalization. Serum-exposed features were screened by comparing blank serum, ECJ-treated serum, and ECJ extract. Prototype components were defined as features detected in both ECJ extract and ECJ-treated serum with matched retention behavior, together with a peak area fold change of ≥2 in ECJ-treated serum relative to blank serum or absence in blank serum. Potential metabolites were defined as features detected in ECJ-treated serum but not in ECJ extract, with a peak area fold change of ≥2 relative to blank serum or absence in blank serum and stable retention behavior. Feature annotation was performed using accurate mass, isotope pattern, adduct assignment, retention time, MS/MS fragmentation when available, and database/reference matching. Unless confirmed by authentic standard co-elution and matched standard MS/MS, serum-exposed chemical assignments were reported as putative annotations.

### 4.8. Isolation and Culture of Primary Rat PASMCs

Primary rat PASMCs were isolated from Sprague-Dawley rats using the tissue explant method. Pulmonary artery branches were isolated under aseptic conditions. The adventitia was carefully removed, and the endothelial layer was gently scraped off to obtain the medial layer. The medial tissue was cut into small explants and placed on the bottom of culture flasks. Explants were cultured in high-glucose DMEM supplemented with 20% fetal bovine serum and 1% penicillin-streptomycin at 37 °C in a humidified incubator containing 5% CO_2_. The identity of primary PASMCs was confirmed by α-smooth muscle actin (α-SMA) immunocytochemical staining. Cells showed typical spindle-shaped morphology and positive cytoplasmic α-SMA staining. Only cells from passages 3–8 were used for experiments.

### 4.9. CoCl_2_-Induced Hypoxia-Mimetic PASMC Model and Ginkgolide J Treatment

Ginkgolide J was dissolved in DMSO to prepare a stock solution and stored at −20 °C. CoCl_2_ was dissolved in PBS. Before each experiment, stock solutions were diluted to the desired working concentrations with serum-free DMEM. The final concentration of DMSO in all groups was maintained at ≤0.1%.

To establish a hypoxia-mimetic model, PASMCs were treated with CoCl_2_ at 100 μM for 24 h. This model was used to mimic hypoxia-associated cellular stress rather than to fully reproduce physiological low-oxygen culture conditions.

### 4.10. CCK-8 Assay and IC_50_ Estimation

Cell viability/metabolic activity was assessed using the CCK-8 assay. PASMCs were seeded into 96-well plates at a density of 1 × 10^4^ cells per well. After 24 h of attachment, the medium was replaced with serum-free DMEM for 24 h to synchronize the cells. All treatments were performed for 24 h.

For cytotoxicity assessment, PASMCs were treated with ginkgolide J at concentrations ranging from 0.1 to 62.5 μM. For assessment under hypoxia-mimetic conditions, PASMCs were treated with CoCl_2_ (100 μM) in the presence or absence of ginkgolide J at concentrations ranging from 0.1 to 62.5 μM.

The IC_50_ value was calculated using GraphPad Prism 9.5.0 by fitting normalized response data to a log(inhibitor) versus response model with variable slope using a four-parameter logistic equation.

### 4.11. Wound Healing Assay

PASMC migration-related wound closure was evaluated using a wound healing assay. Cells were seeded into six-well plates and cultured until approximately 80% confluent. Linear scratches were generated using a sterile 200 μL pipette tip. Detached cells were removed by washing with PBS, and fresh serum-free medium containing the corresponding treatments was added. To reduce the contribution of proliferation to wound closure, the assay was performed in serum-free medium during the 24 h observation period. Images were captured at 0 and 24 h after scratching. Wound closure was quantified using Fiji/ImageJ 1.54p (National Institutes of Health, Bethesda, MD, USA). Wound closure was calculated as [(wound area at 0 h − wound area at 24 h)/wound area at 0 h] × 100%.

### 4.12. Ki-67 Immunofluorescence

PASMC proliferation was further assessed by Ki-67 immunofluorescence. After treatment, cells were fixed, permeabilized, blocked, and incubated with an anti-Ki-67 primary antibody overnight at 4 °C. Cells were then incubated with the corresponding fluorescence-conjugated secondary antibody, and nuclei were counterstained with DAPI. Fluorescence images of the same fields were sequentially acquired in the DAPI and Ki-67 channels using identical exposure time, gain, and channel settings within each experiment. For each group, randomly selected fields were imaged and analyzed using Fiji/ImageJ 1.54p (National Institutes of Health, Bethesda, MD, USA). by investigators blinded to the treatment groups. DAPI-positive nuclei were used to determine the total number of nuclei. Ki-67-positive nuclei were defined as DAPI-positive nuclei with nuclear Ki-67 fluorescence intensity greater than the mean background intensity plus three standard deviations, determined from cell-free background regions in the corresponding Ki-67 channel. When necessary, automatic segmentation was manually checked to exclude overlapping nuclei, debris, or non-nuclear background signals. The percentage of Ki-67-positive cells was calculated as the number of Ki-67-positive nuclei divided by the total number of DAPI-positive nuclei × 100%.

### 4.13. DARTS-MS-Based Chemoproteomic Target Identification

DARTS-MS was performed to identify candidate ginkgolide J-associated proteins based on ligand-associated changes in protease susceptibility. PASMC total protein lysates were prepared under native conditions to preserve protein conformation. The DMSO group served as the vehicle control without protease or ginkgolide J treatment. The protease-only group was treated with protease but without ginkgolide J. For the ginkgolide J plus protease group, PASMC protein lysates were incubated with ginkgolide J at a final concentration of 25 μM for 1 h, with the final DMSO concentration maintained at ≤0.1%. The concentration of 25 μM was selected as an exploratory DARTS-MS screening concentration to facilitate detection of ligand-associated protease-susceptibility changes in complex PASMC lysates. Limited proteolysis was then performed using Pronase E from *Streptomyces griseus* (Shanghai Yuanye Bio-Technology Co., Ltd.; Cat# S10014-250 mg) at an enzyme-to-protein ratio of 1:700 for 30 min at 37.5 °C. The proteolysis reaction was terminated by adding a protease inhibitor cocktail (Solarbio, Cat# P6730).

Proteins remaining after limited proteolysis were processed for DIA-MS analysis. Each group included three biological replicates. DIA-MS detection was performed using a Vanquish Neo UHPLC system coupled to an Orbitrap Astral Zoom mass spectrometer (Thermo Fisher Scientific, Waltham, MA, USA). Raw MS files were processed using DIA-NN version 2.2.0 against the UniProt *Rattus norvegicus* FASTA database. The DARTS-MS raw and processed file descriptions are provided in Supplementary Table S7. Trypsin was used as the digestion enzyme for MS sample preparation. Carbamidomethylation of cysteine was set as a fixed modification, whereas methionine oxidation and protein N-terminal acetylation were set as variable modifications. The false discovery rate was controlled at 1% at the PSM and peptide levels. The DARTS-MS experiment included three groups, namely DMSO vehicle control, protease-only treatment, and ginkgolide J plus protease treatment, with three biological replicates per group. Detailed DARTS-MS sample metadata and experimental design are provided in Supplementary Table S1. After preprocessing, 7081 proteins were quantified. An initial exploratory group-level screening was performed using one-way ANOVA with *p* < 0.05, yielding 2560 proteins. Because this initial screening was intended for exploratory report-level filtering, downstream candidate prioritization was further performed using pairwise protease-susceptibility analysis with adjusted *p* value and fold-change thresholds, as described below.

### 4.14. Candidate Protein Screening and Functional Annotation

Initial exploratory group-level screening was performed among the DMSO control, protease-only, and ginkgolide J plus protease groups using one-way ANOVA with *p* < 0.05 as the preliminary threshold, yielding 2560 proteins. Because DARTS-MS measures ligand-associated changes in protease susceptibility rather than steady-state protein expression, these proteins were not interpreted as conventional differentially expressed proteins. Instead, they were used as an exploratory protein set for subsequent pairwise protease-susceptibility analysis.

To further prioritize proteins with a DARTS-consistent response pattern, we performed a pairwise protease-susceptibility analysis. Proteins were considered strict ginkgolide J-associated protease-susceptibility candidates if they were significantly altered in the protease-only group compared with the DMSO control group and were oppositely regulated in the ginkgolide J plus protease group compared with the protease-only group under adjusted *p* value < 0.05 and |log_2_ fold change| ≥ 0.263. This analysis identified 1235 strict ginkgolide J-associated protease-susceptibility candidates, including 1210 proteins showing decreased residual abundance after protease digestion and increased residual abundance after ginkgolide J incubation, consistent with a putative protease-protection/stabilization pattern, and 25 proteins showing the opposite pattern.

Functional annotation was performed using the clusterProfiler package (version 4.20.0) in R software (version 4.6.0; R Foundation for Statistical Computing, Vienna, Austria). GO term-to-gene annotation was derived from org.Rn.eg.db (version 3.23.0), and GO enrichment analysis was performed using clusterProfiler: enricher. KEGG pathway enrichment analysis was performed using clusterProfiler: enrichKEGG with the organism code rno. All quantified DARTS-MS proteins were used as the background universe. Benjamini–Hochberg correction was applied for multiple testing.

### 4.15. Western Blot Analysis

PASMCs were lysed in RIPA buffer supplemented with protease and phosphatase inhibitors. Equal amounts of protein were separated by SDS-PAGE and transferred onto PVDF membranes. Membranes were blocked, incubated with primary antibodies overnight at 4 °C, and then incubated with HRP-conjugated secondary antibodies. Signals were detected using enhanced chemiluminescence. Western blot band intensities were quantified by densitometric analysis using ImageJ. For ERK1/2, the total immunoreactive region corresponding to the closely migrating ERK1 and ERK2 isoforms was quantified as a combined ERK1/2 signal. Only linear brightness and contrast adjustments were applied uniformly to the entire blot image when necessary for figure presentation. No selective enhancement, deletion, splicing, or local adjustment was performed. Full-length uncropped blot images are provided in Supplementary Figure S7. Total ERK1/2 and MEK1 were normalized to β-actin. ERK1/2 pathway activation was evaluated using the ratios of phospho-ERK1/2 to total ERK1/2 and phospho-MEK1 to total MEK1.

For inhibitor validation, PASMCs were divided into control, CoCl_2_ model, ginkgolide J, U0126, PD98059, U0126 plus ginkgolide J, and PD98059 plus ginkgolide J groups. Treatments were performed for 24 h using CoCl_2_ at 100 μM, ginkgolide J at 2.5 μM, U0126 at 10 μM, and PD98059 at 20 μM.

### 4.16. Statistical Analysis

Data are presented as mean ± standard deviation. Statistical analyses were performed using GraphPad Prism 9.5.0, SPSS v26.0, and R software (version 4.6.0; R Foundation for Statistical Computing, Vienna, Austria). Normality and homogeneity of variance were assessed before group comparisons. One-way ANOVA followed by Dunnett’s multiple comparisons test was used for multiple-group comparisons when appropriate. Exact *p* values are provided where available. A value of *p* < 0.05 was considered statistically significant.

## 5. Conclusions

In conclusion, this study establishes a serum pharmacochemistry- and DARTS-MS chemoproteomics-guided framework for linking ECJ exposure to PASMC-based mechanistic validation. ECJ attenuated hypobaric hypoxia-induced pulmonary hemodynamic dysfunction, right ventricular hypertrophy, and pulmonary vascular remodeling in rats. Serum pharmacochemistry detected 47 serum-exposed chemical features after ECJ administration, including one prototype feature putatively annotated as ginkgolide J, which provided the rationale for selecting ginkgolide J for downstream validation. In PASMCs, ginkgolide J suppressed CoCl_2_-induced PASMC activation. DARTS-MS identified 1235 strict ginkgolide J-associated protease-susceptibility candidates, whose functional annotation highlighted mitochondrial energy metabolism, oxidative phosphorylation, cytoskeletal organization, vesicular transport, and proteostasis-related processes. MEK/ERK- and MAPK-associated candidates and Western blot validation supported the involvement of MEK1/ERK1/2 signaling in the PASMC response to ginkgolide J. These findings support ginkgolide J as a candidate compound with PASMC anti-remodeling activity and demonstrate the value of DARTS-MS chemoproteomics for connecting serum-exposed natural product candidates with disease-relevant protein target networks.

## Data Availability

The DARTS-MS raw data, processed quantitative matrices, candidate protein tables, and R scripts used for functional annotation have been deposited in the ProteomeXchange Consortium via the iProX partner repository under accession number PXD078800 and iProX project ID IPX0017397000.

## Supplementary Materials

The following supporting information can be downloaded at: https://www.mdpi.com/article/10.3390/ijms27135815/s1.

## Abbreviations

The following abbreviations are used in this manuscript: ANOVA, analysis of variance; ARRIVE, Animal Research: Reporting of In Vivo Experiments; CCK-8, Cell Counting Kit-8; CMC-Na, sodium carboxymethyl cellulose; CoCl_2_, cobalt chloride; DAPI, 4′,6-diamidino-2-phenylindole; DARTS-MS, drug affinity responsive target stability coupled with mass spectrometry; DIA-MS, data-independent acquisition mass spectrometry; DMEM, Dulbecco’s modified Eagle’s medium; DMSO, dimethyl sulfoxide; ECJ, ethanol extract of *Caragana jubata*; ERK1/2, extracellular signal-regulated kinase 1/2; EVG, Elastica van Gieson staining; FBS, fetal bovine serum; GO, Gene Ontology; HESI, heated electrospray ionization; HPH, hypoxic pulmonary hypertension; HAPC, high-altitude polycythemia; HRP, horseradish peroxidase; IC_50_, half-maximal inhibitory concentration; KEGG, Kyoto Encyclopedia of Genes and Genomes; LC-MS, liquid chromatography–mass spectrometry; LC-MS/MS, liquid chromatography–tandem mass spectrometry; LV + S, left ventricle plus septum; MEK1, mitogen-activated protein kinase kinase 1; mPAP, mean pulmonary arterial pressure; MS/MS, tandem mass spectrometry; GJ, ginkgolide J; PASMCs, pulmonary artery smooth muscle cells; PBS, phosphate-buffered saline; PVDF, polyvinylidene difluoride; RV, right ventricle; RVHI, right ventricular hypertrophy index; SD, standard deviation; SDS-PAGE, sodium dodecyl sulfate–polyacrylamide gel electrophoresis; UPLC, ultra-performance liquid chromatography; α-SMA, α-smooth muscle actin; CETSA, cell thermal shift assay.
