# Supplementary material for: Serum Pharmacochemistry-Guided DARTS-MS Profiling Reveals Potential Mechanisms of Caragana jubata Against Hypoxic Pulmonary Hypertension
Source: Int J Mol Sci. 2026 Jun 27;27(13):5815. doi: 10.3390/ijms27135815 (PMC13361411; doi:10.3390/ijms27135815)
Supplement: Supplementary file 1 [file ijms-27-05815-s001.zip › Supplementary Figures/Supplementary Figure S7(1).pdf]

Figure 6A

(1)

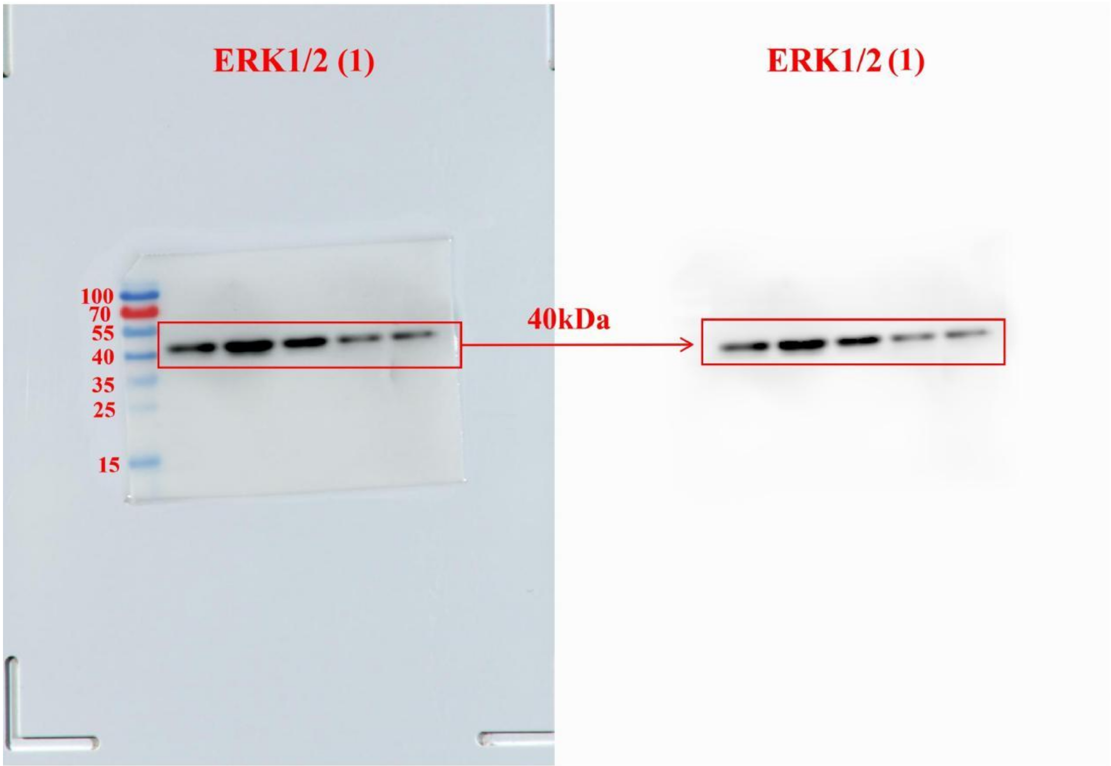
ERK1/2 (1)
ERK1/2 (1)
100
70
55
40
35
25
15
40kDa

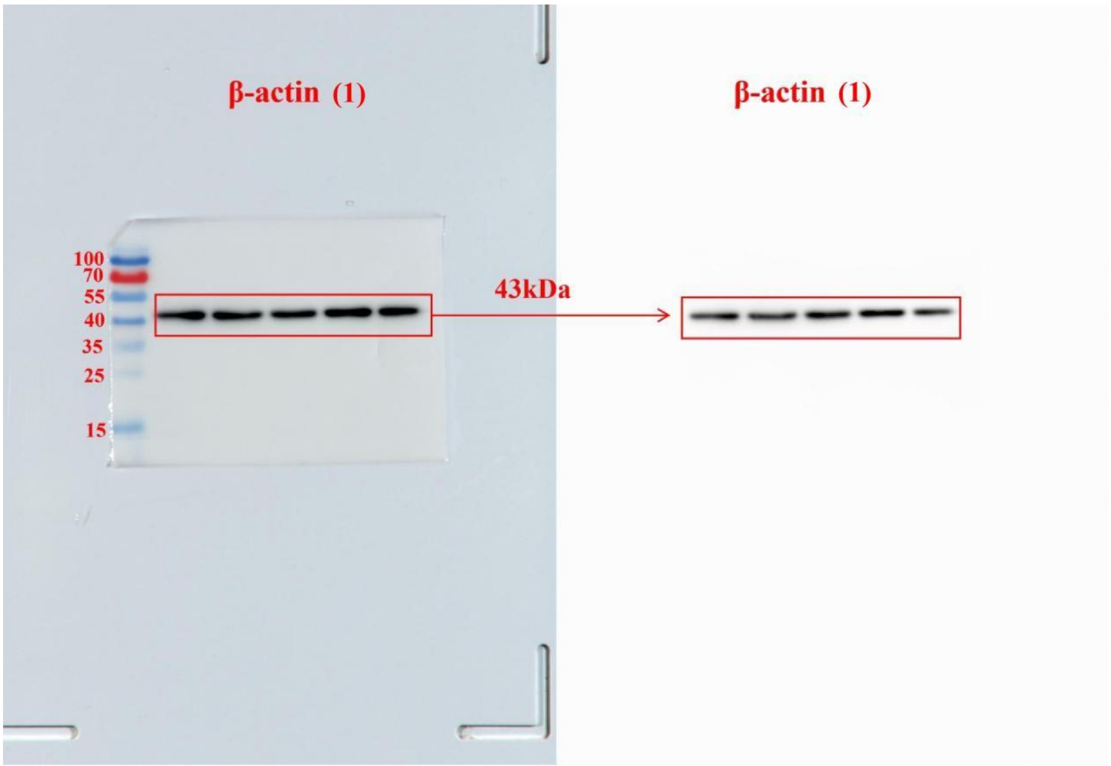
β-actin (1)
β-actin (1)
100
70
55
40
35
25
15
43kDa

(2)

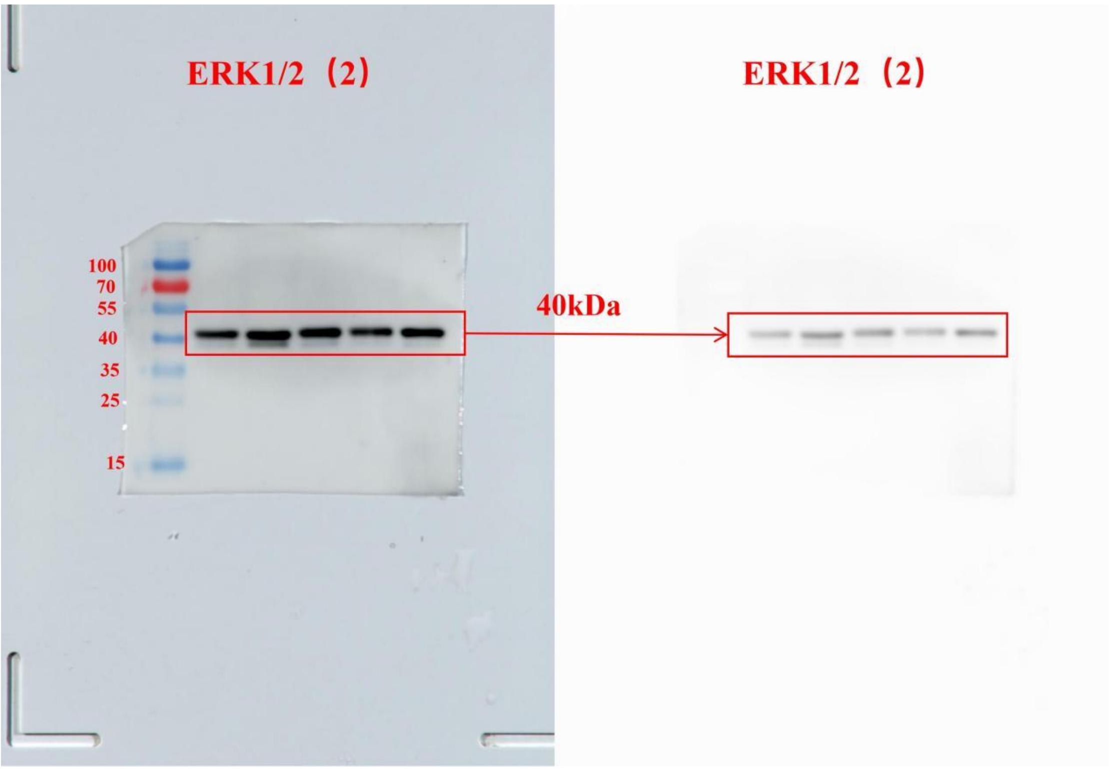
ERK1/2（2）
ERK1/2（2）
100
70
55
40
35
25
15
40kDa

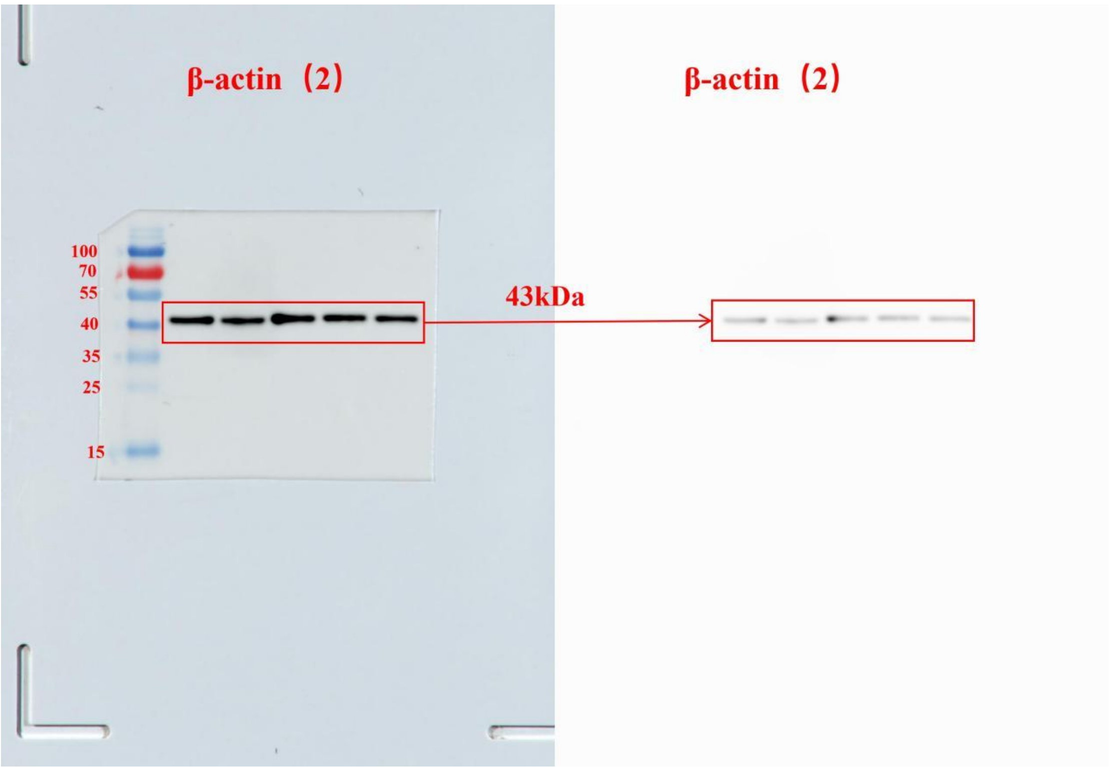
β-actin（2）
β-actin（2）
100
70
55
40
35
25
15
43kDa

(3)

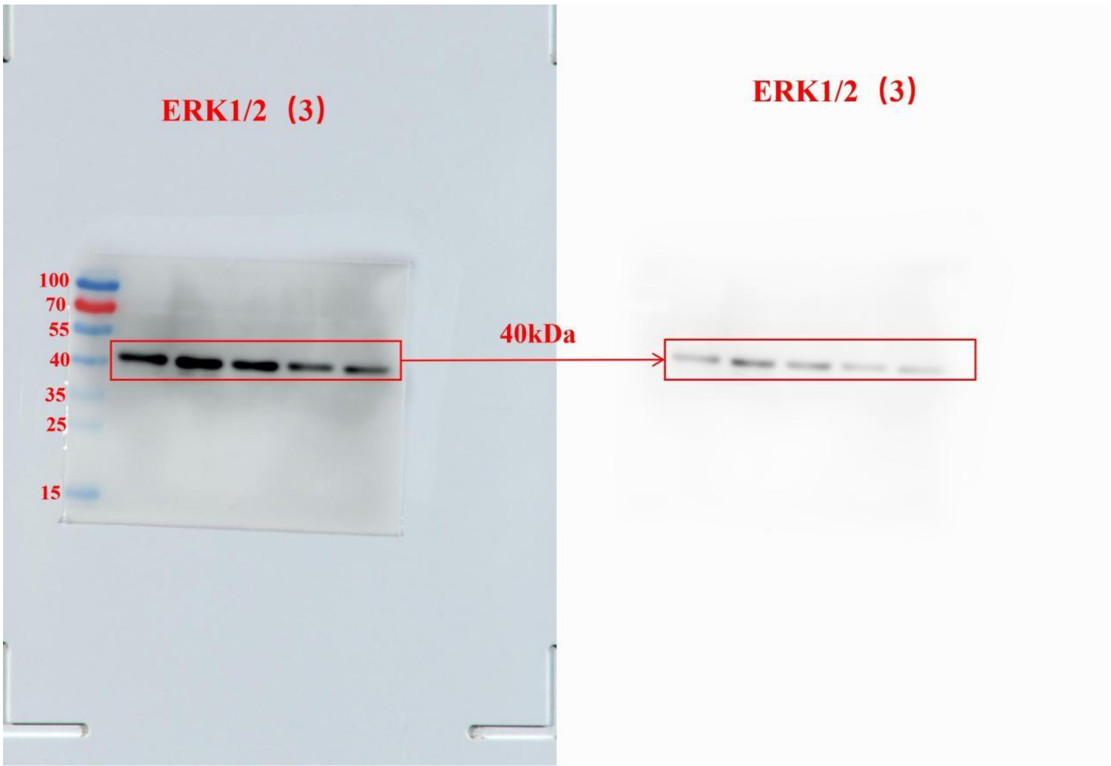
ERK1/2（3）
100
70
55
40
35
25
15
40kDa
ERK1/2（3）

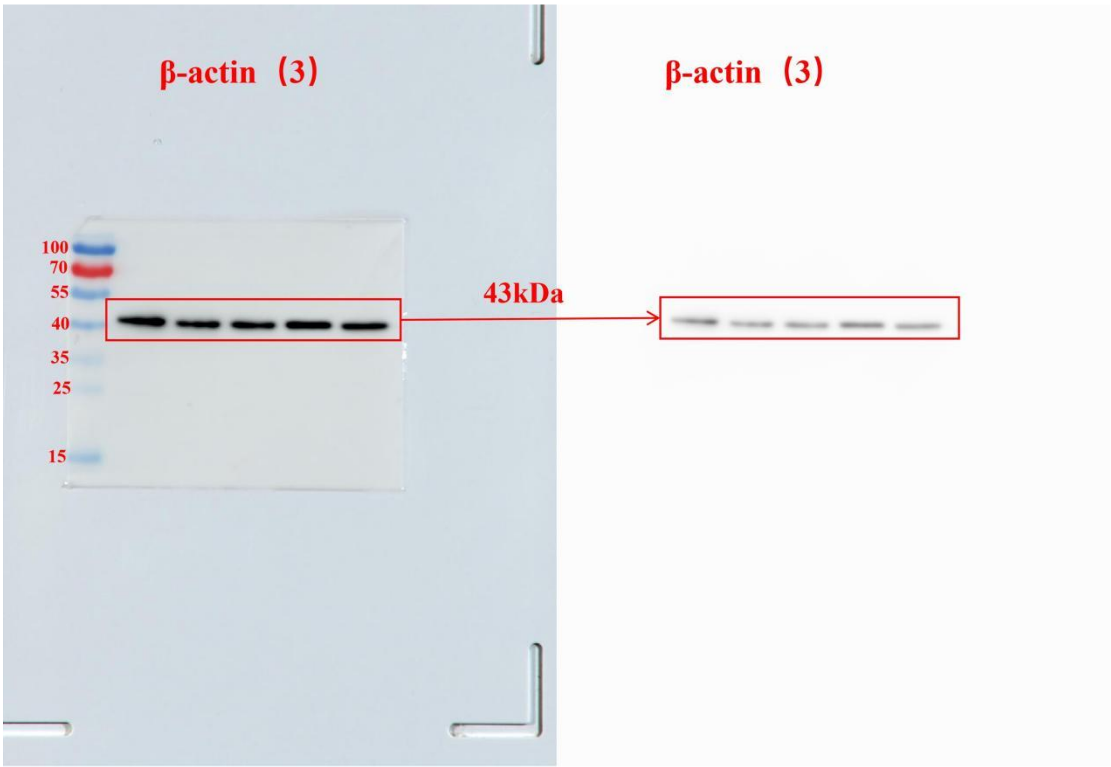
β-actin（3）
100
70
55
40
35
25
15
43kDa
β-actin（3）

Figure 6B

(1)

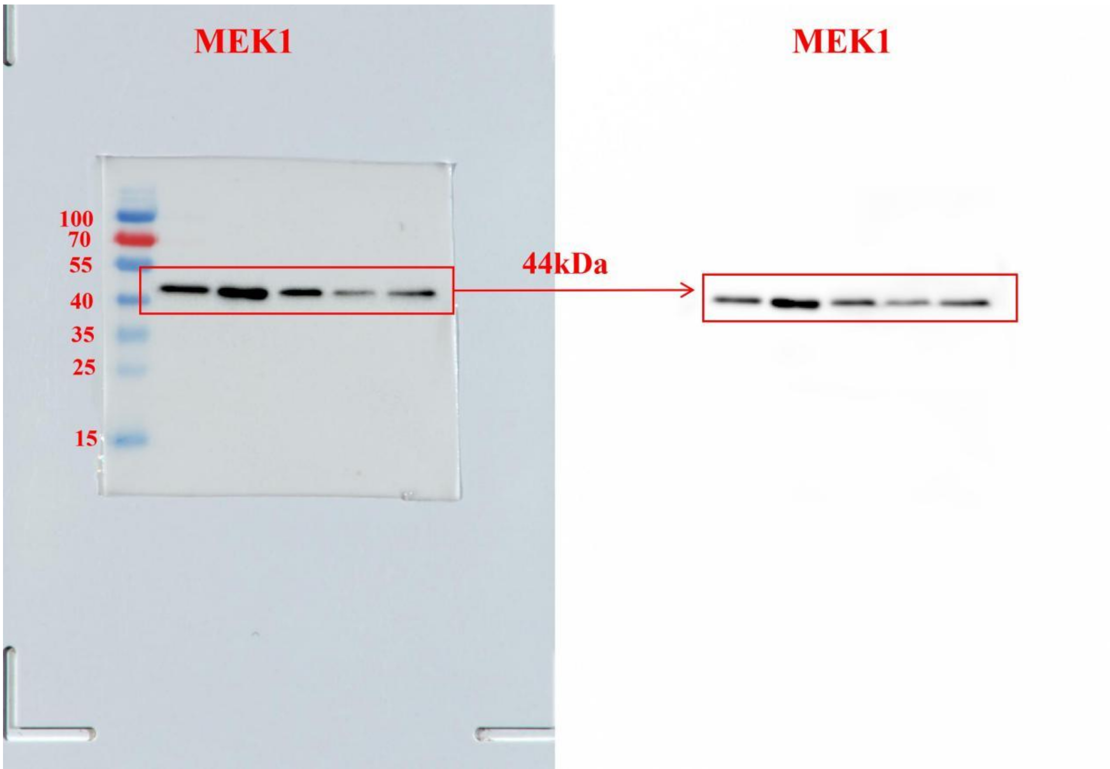
MEK1
100
70
55
40
35
25
15
44kDa
MEK1

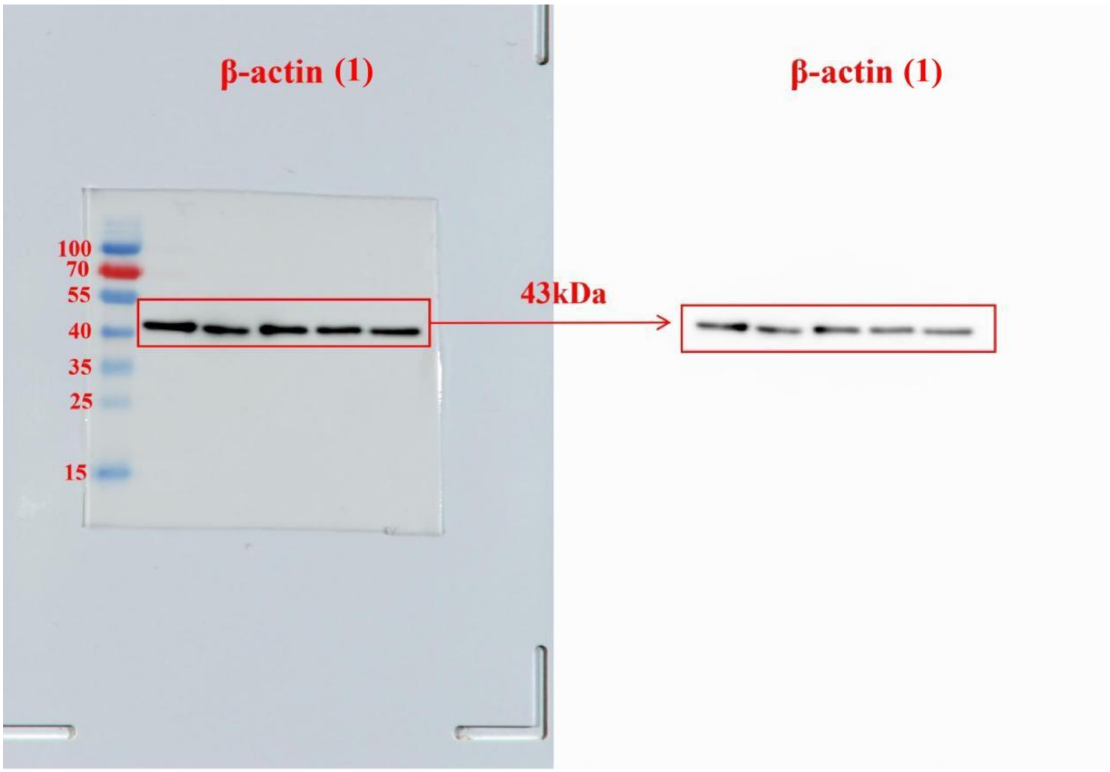
β-actin (1)
100
70
55
40
35
25
15
43kDa
β-actin (1)

(2)

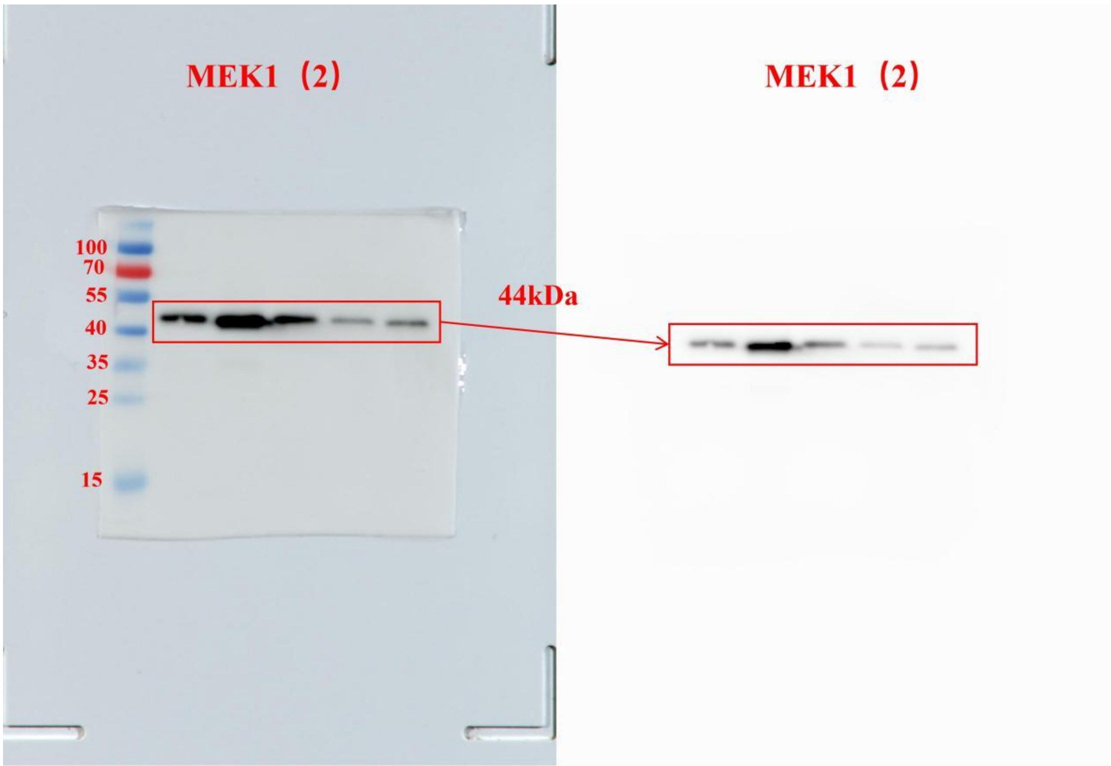
MEK1（2）
100
70
55
40
35
25
15
44kDa
MEK1（2）

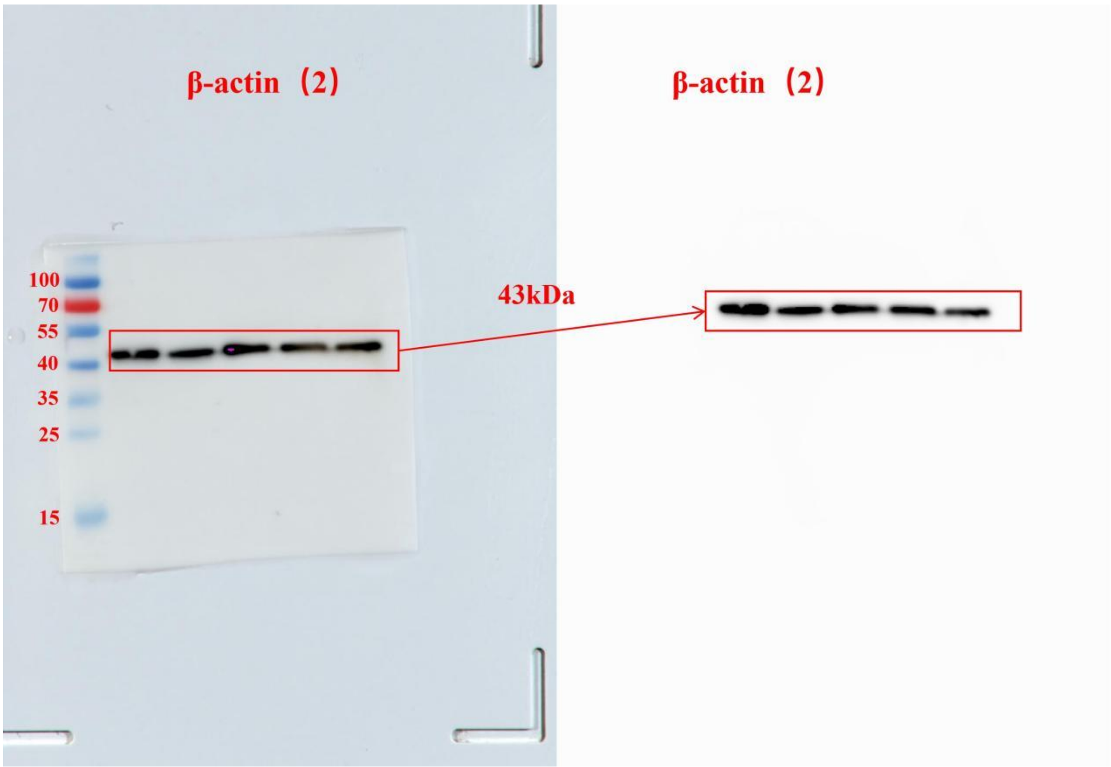
β-actin（2）
100
70
55
40
35
25
15
43kDa
β-actin（2）

(3)

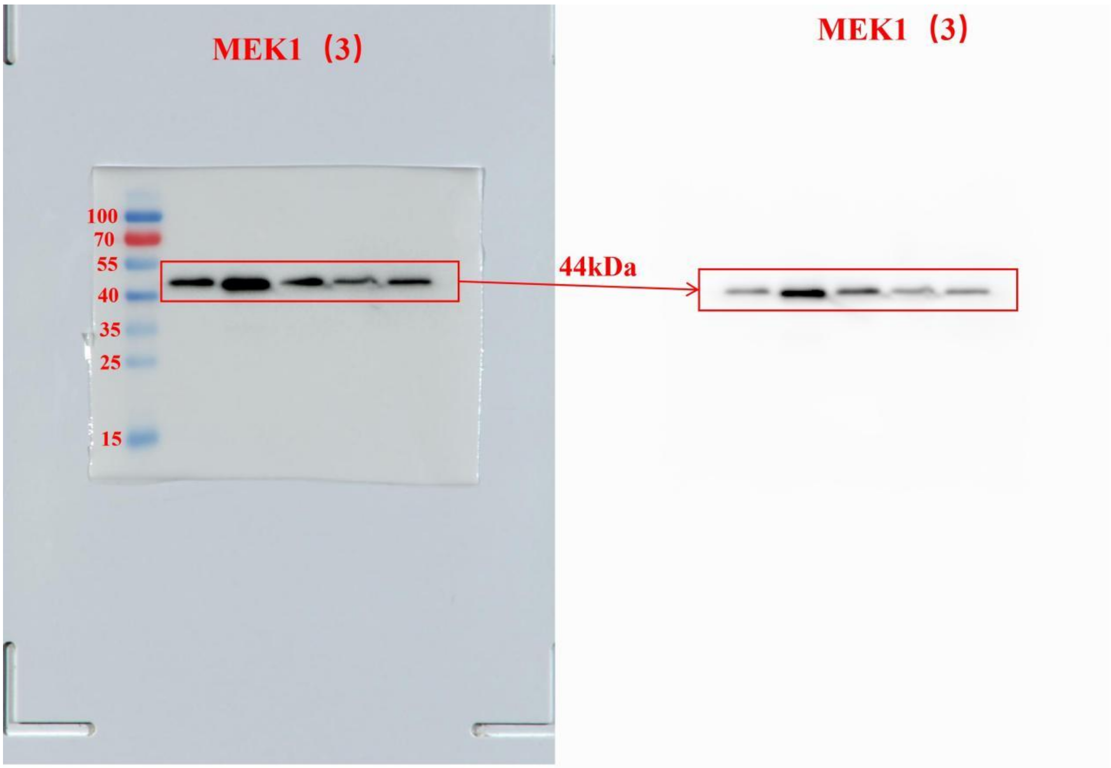
MEK1（3）
100
70
55
40
35
25
15
44kDa
MEK1（3）

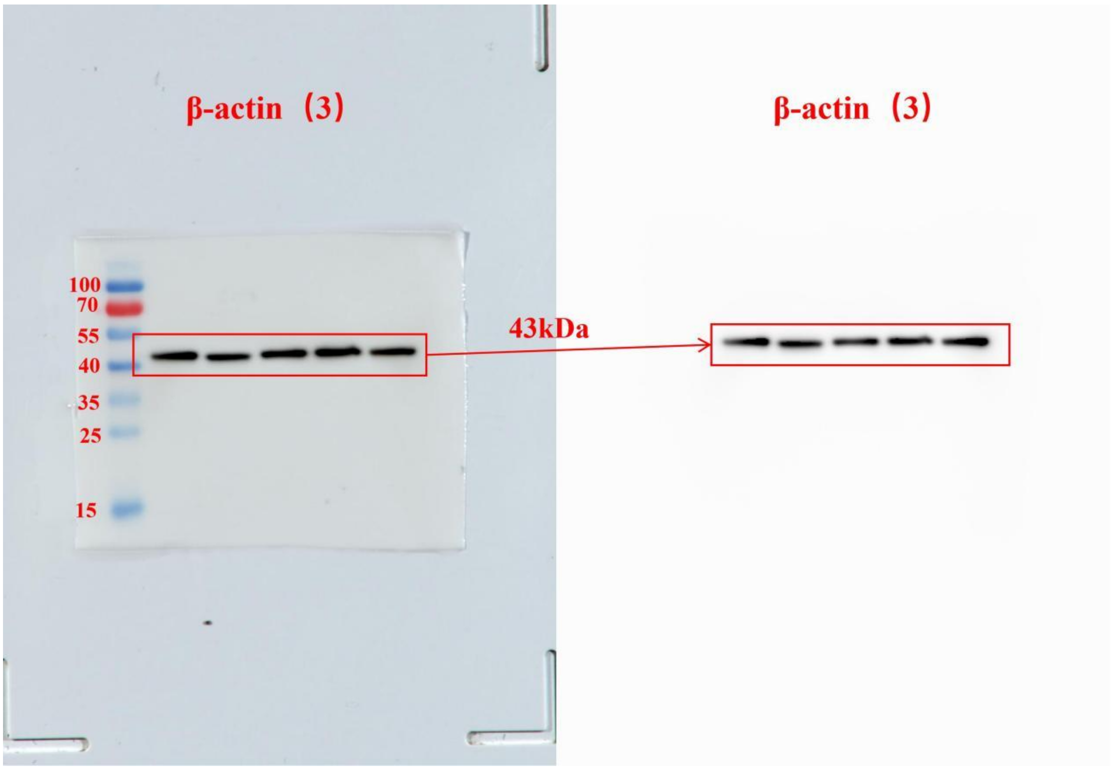
β-actin（3）
100
70
55
40
35
25
15
43kDa
β-actin（3）

Figure 6C

(1)

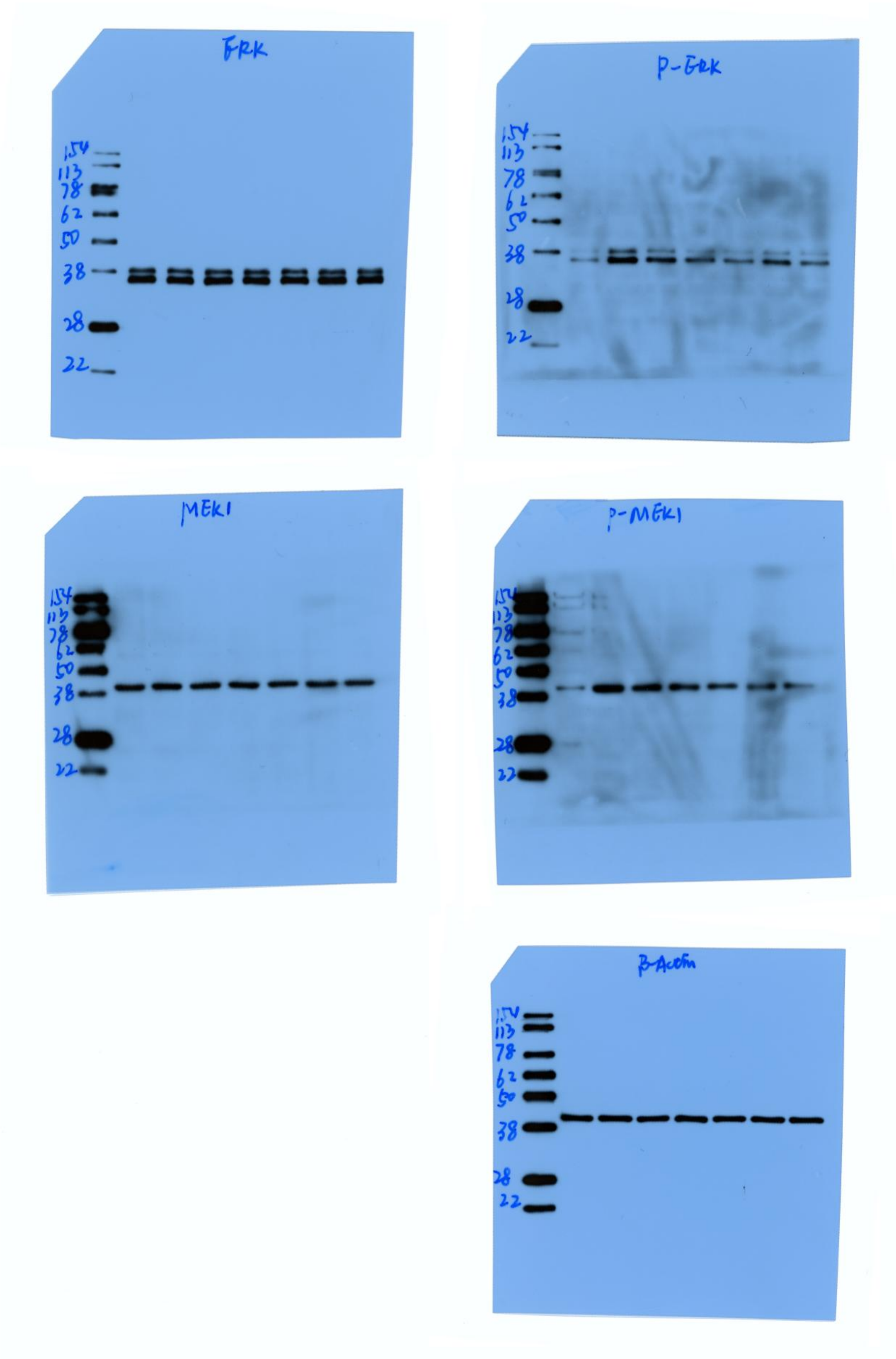

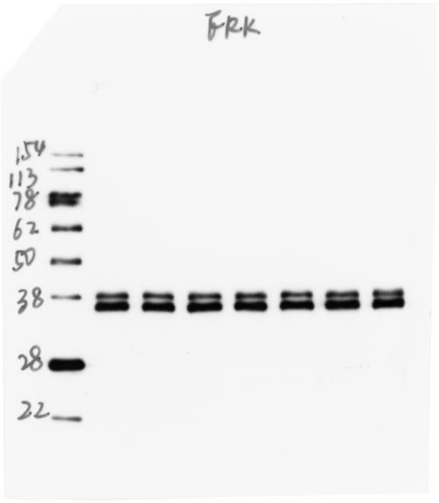
ERK
154
113
78
62
50
38
28
22

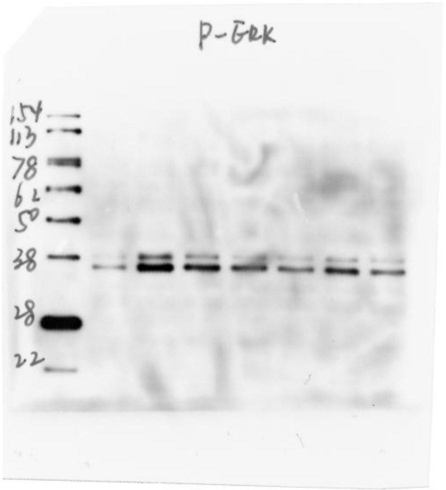
P-ERK
154
113
78
62
50
38
28
22

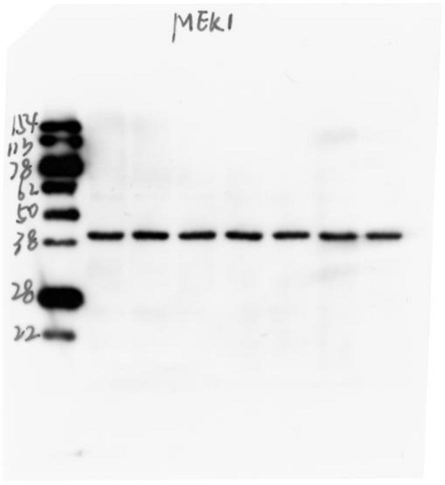
MEK1
154
113
78
62
50
38
28
22

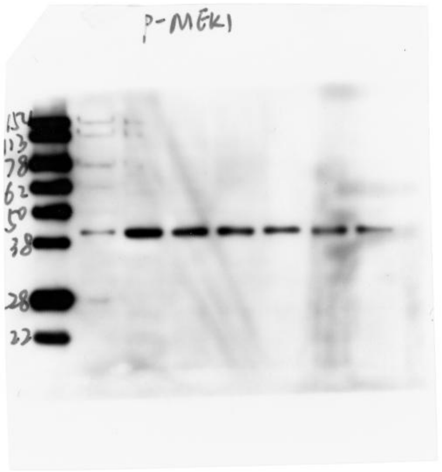
P-MEK1
154
113
78
62
50
38
28
22

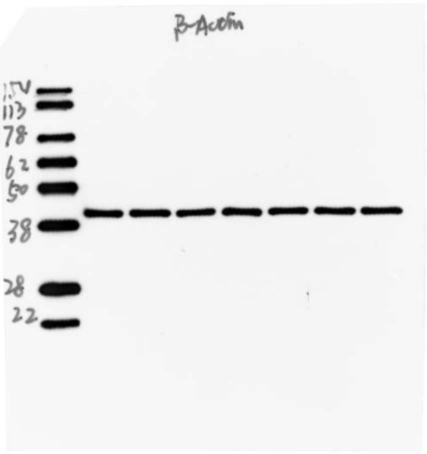
β-Actin
154
113
78
62
50
38
28
22

(2)

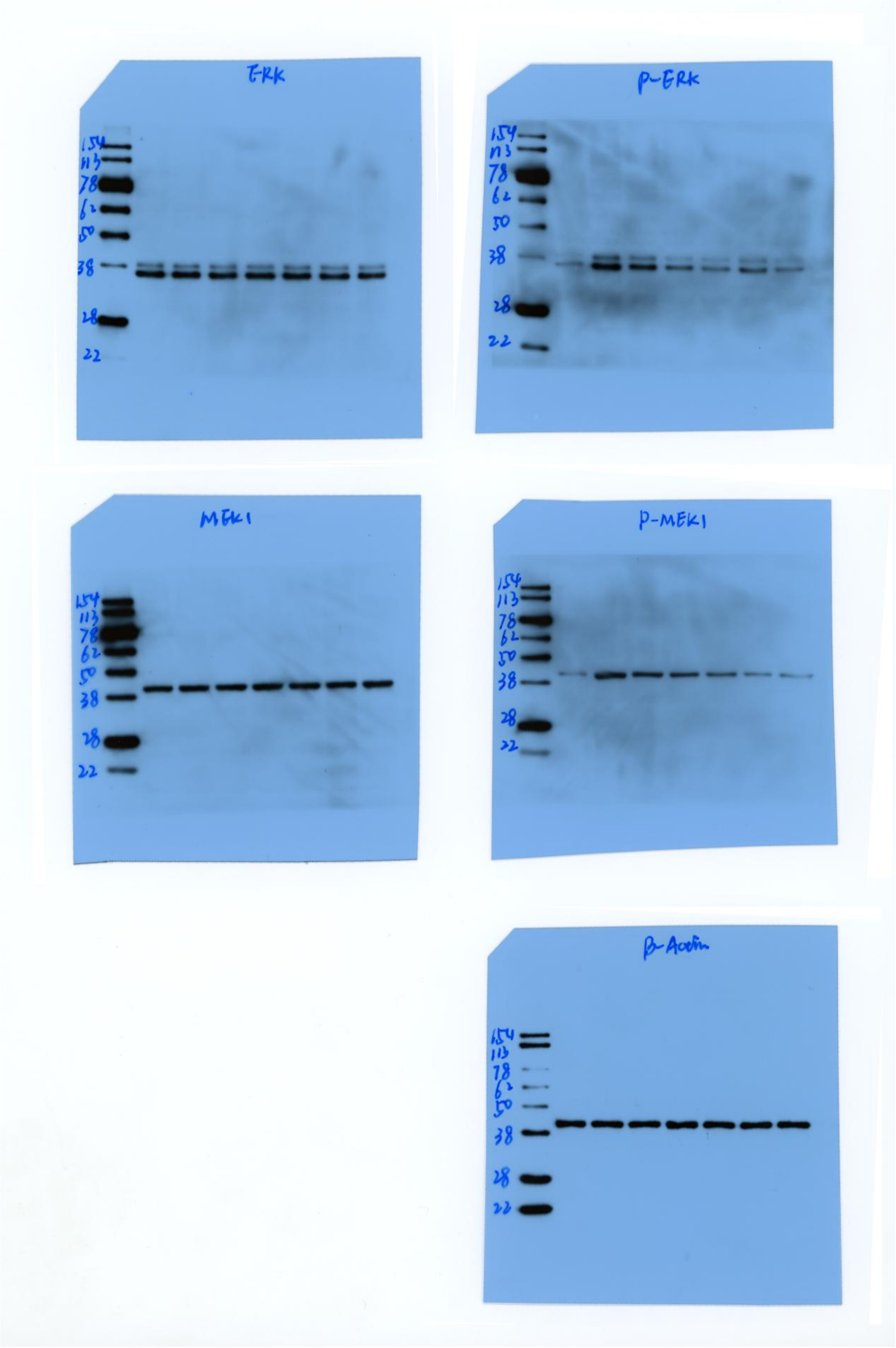

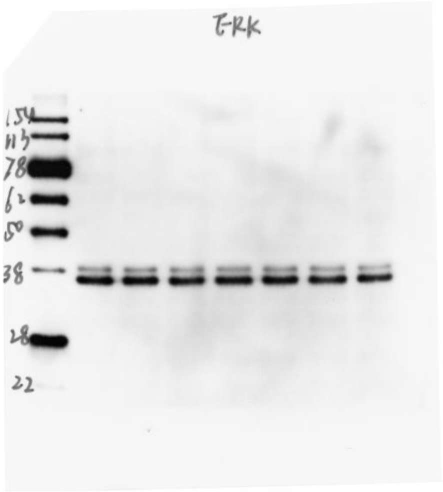
ERK
154
113
78
62
50
38
28
22

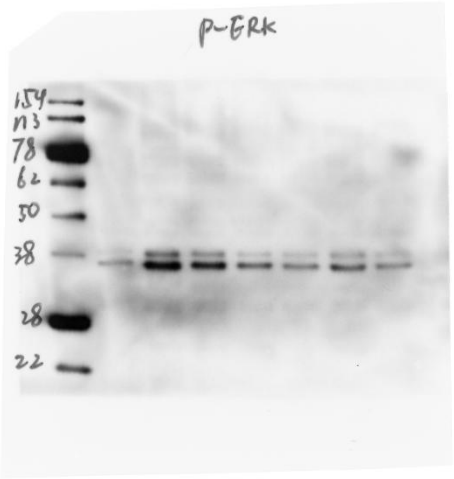
P-ERK
154
113
78
62
50
38
28
22

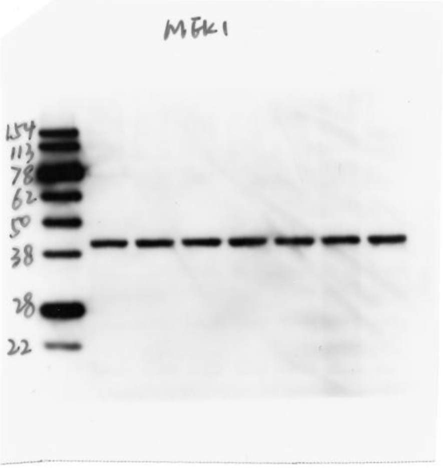
MEK1
154
113
78
62
50
38
28
22

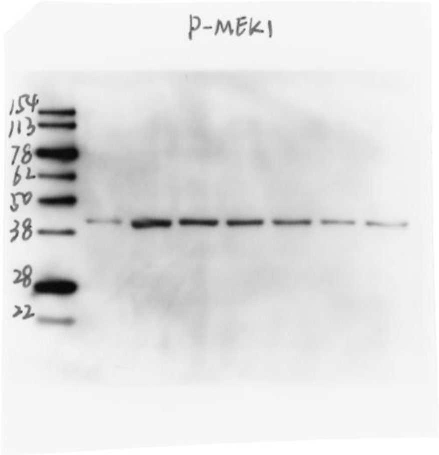
P-MEK1
154
113
78
62
50
38
28
22

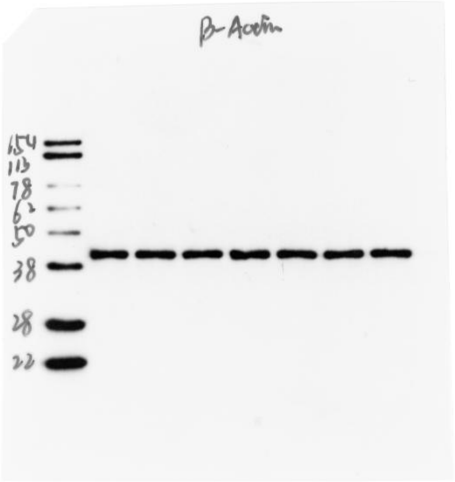
β-Actin
154
113
78
62
50
38
28
22

(3)

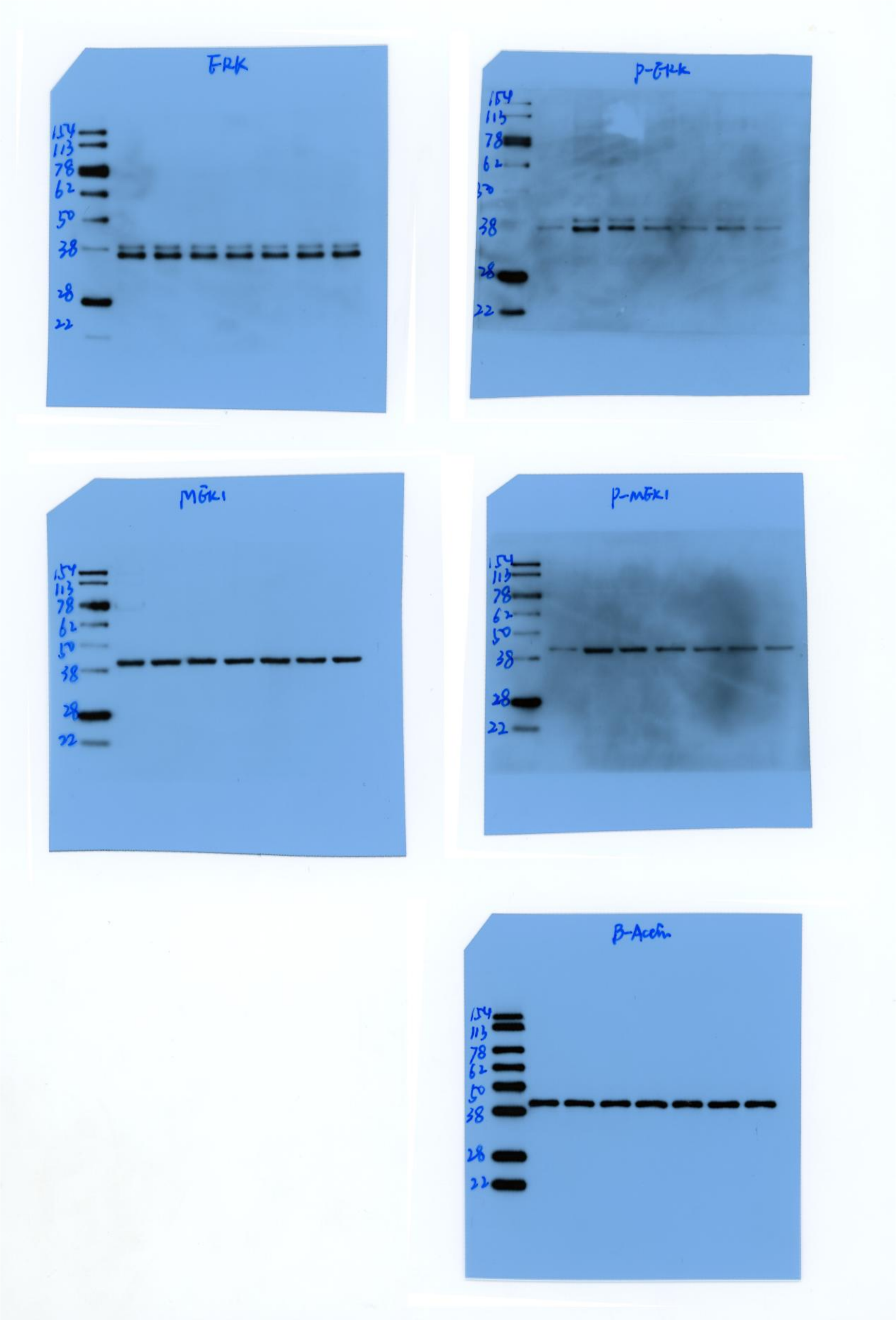

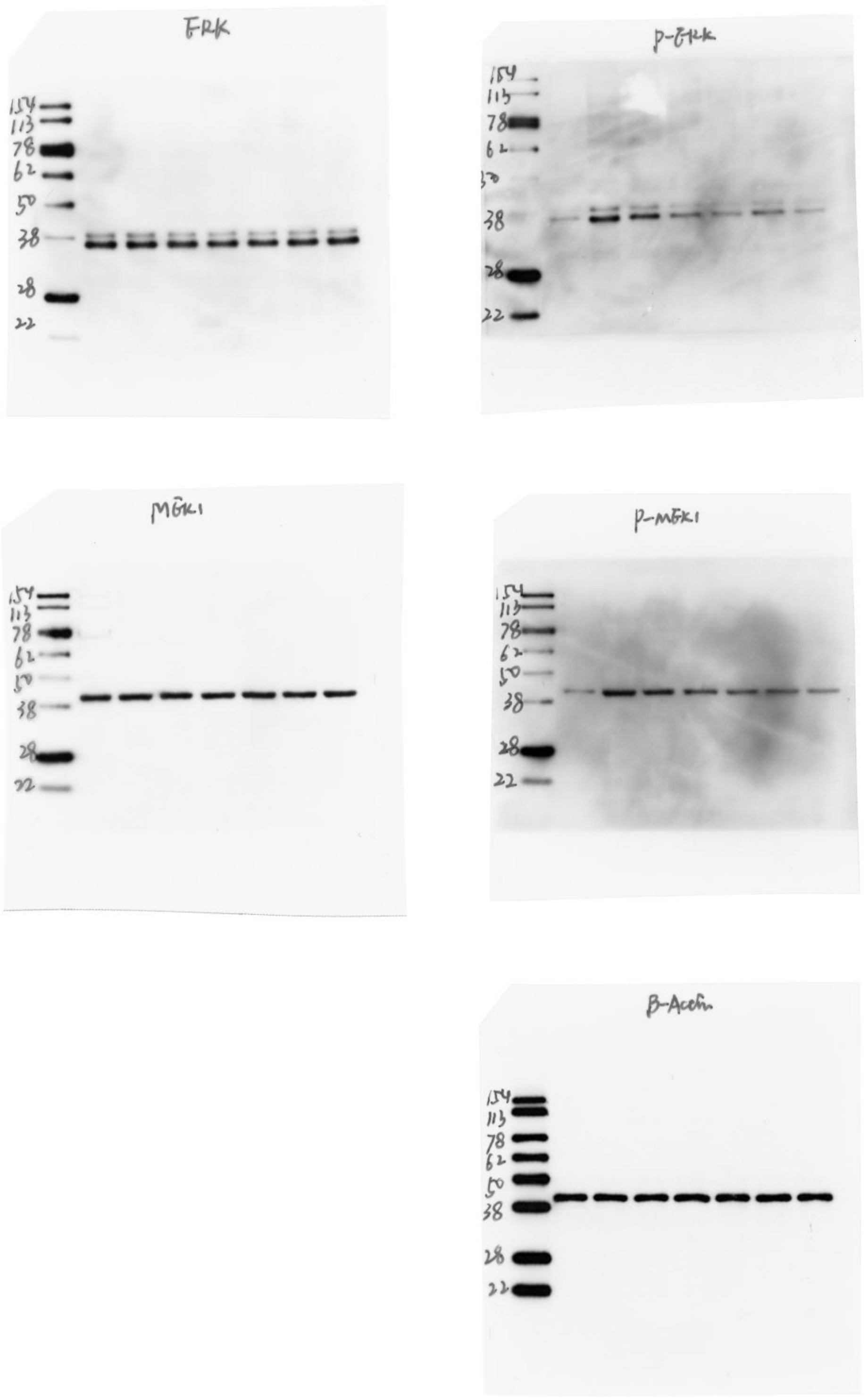

Figure S7. Full-length uncropped western blot images corresponding to Figure 6. Full-length uncropped western blot images corresponding to the cropped western blot panels shown in Figure 6. The cropped regions used in the main figure are indicated where applicable. Molecular weight marker regions are shown where available.
