# Supplementary figures and images for: Serum Pharmacochemistry-Guided DARTS-MS Profiling Reveals Potential Mechanisms of Caragana jubata Against Hypoxic Pulmonary Hypertension
Source: Int J Mol Sci. 2026 Jun 27;27(13):5815. doi: 10.3390/ijms27135815 (PMC13361411; doi:10.3390/ijms27135815)

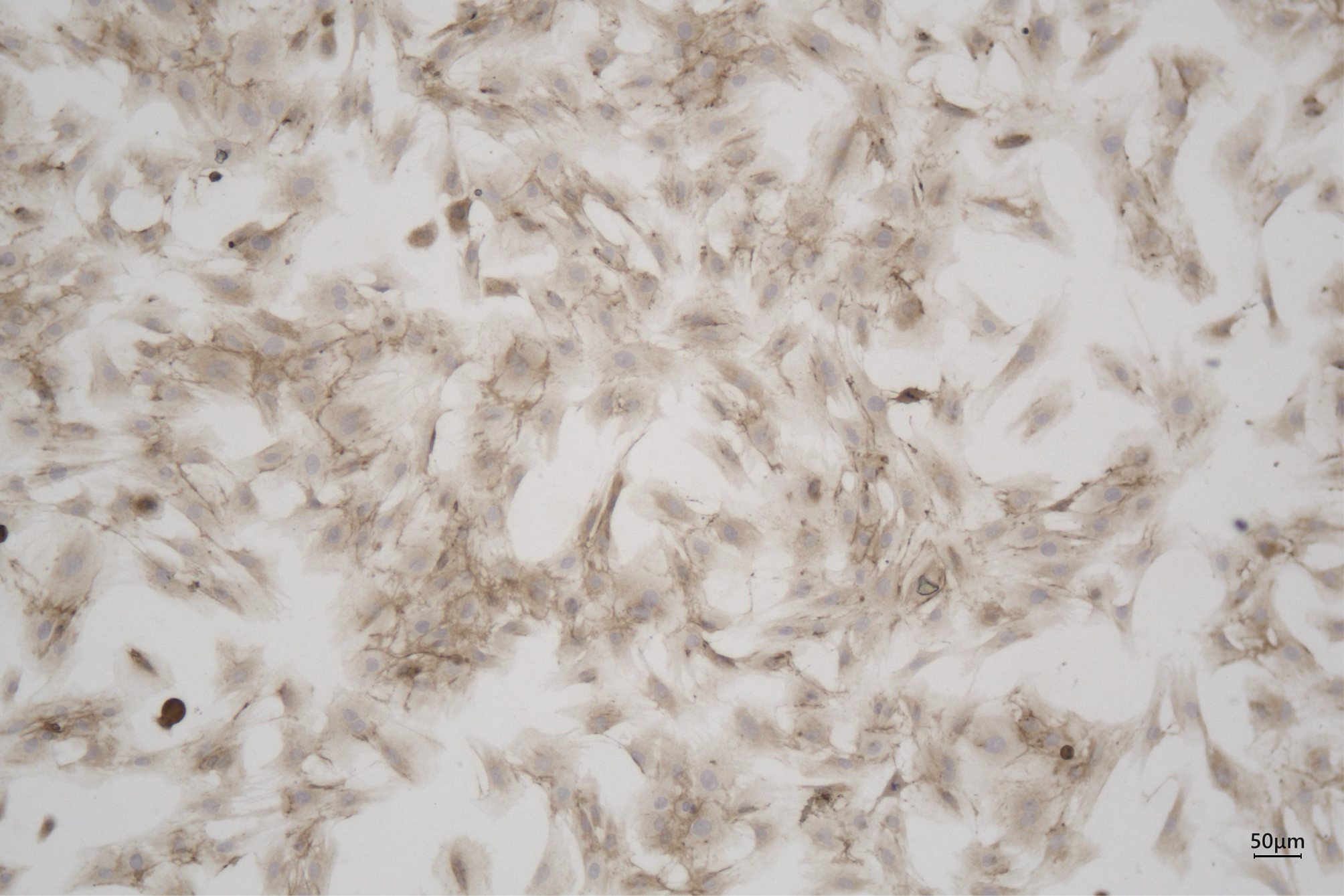

Supplement: Supplementary file 1 [file ijms-27-05815-s001.zip › Supplementary Figures/Supplementary Figure S1.tif]

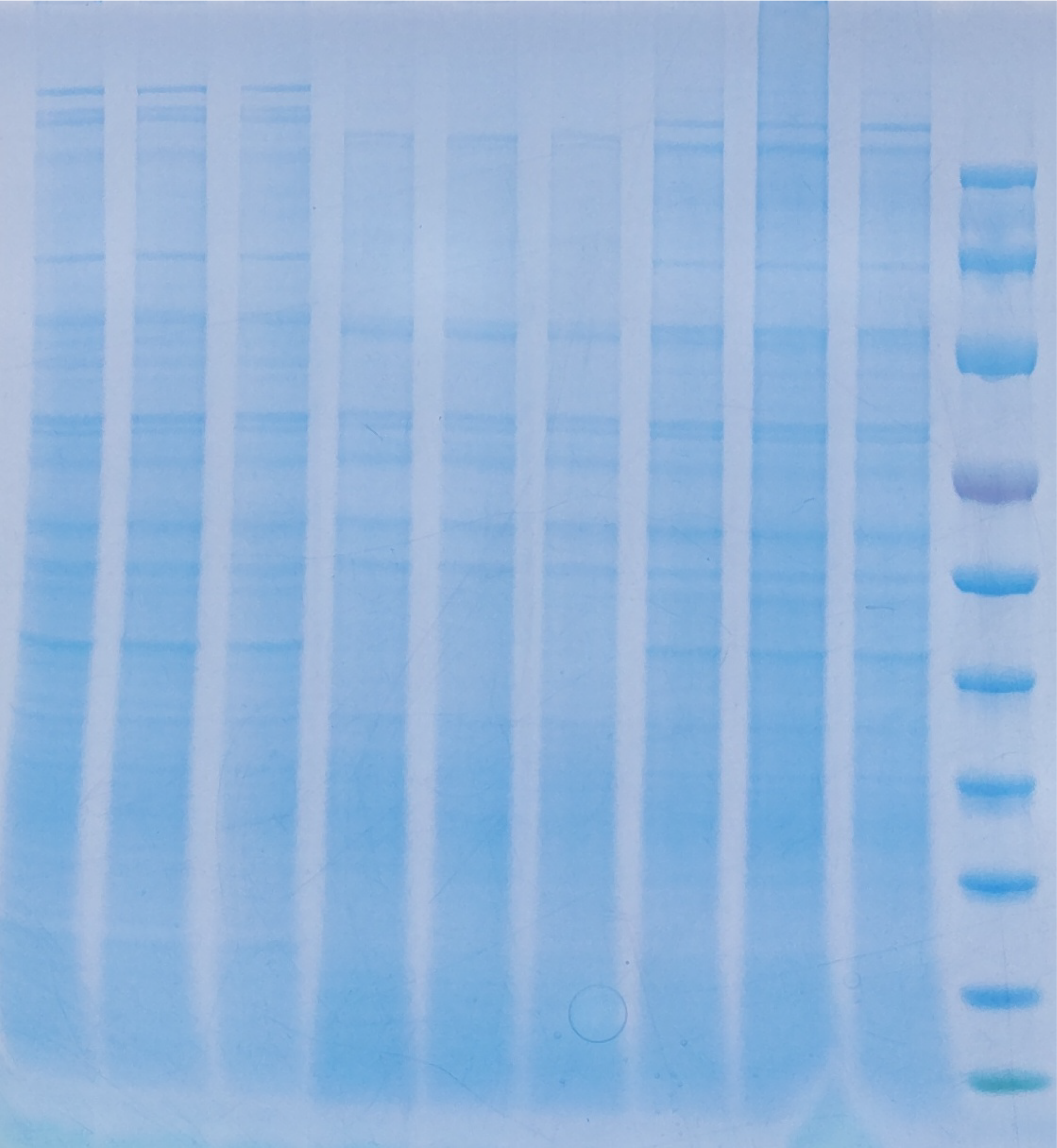

Supplement: Supplementary file 1 [file ijms-27-05815-s001.zip › Supplementary Figures/Supplementary Figure S2.tif]

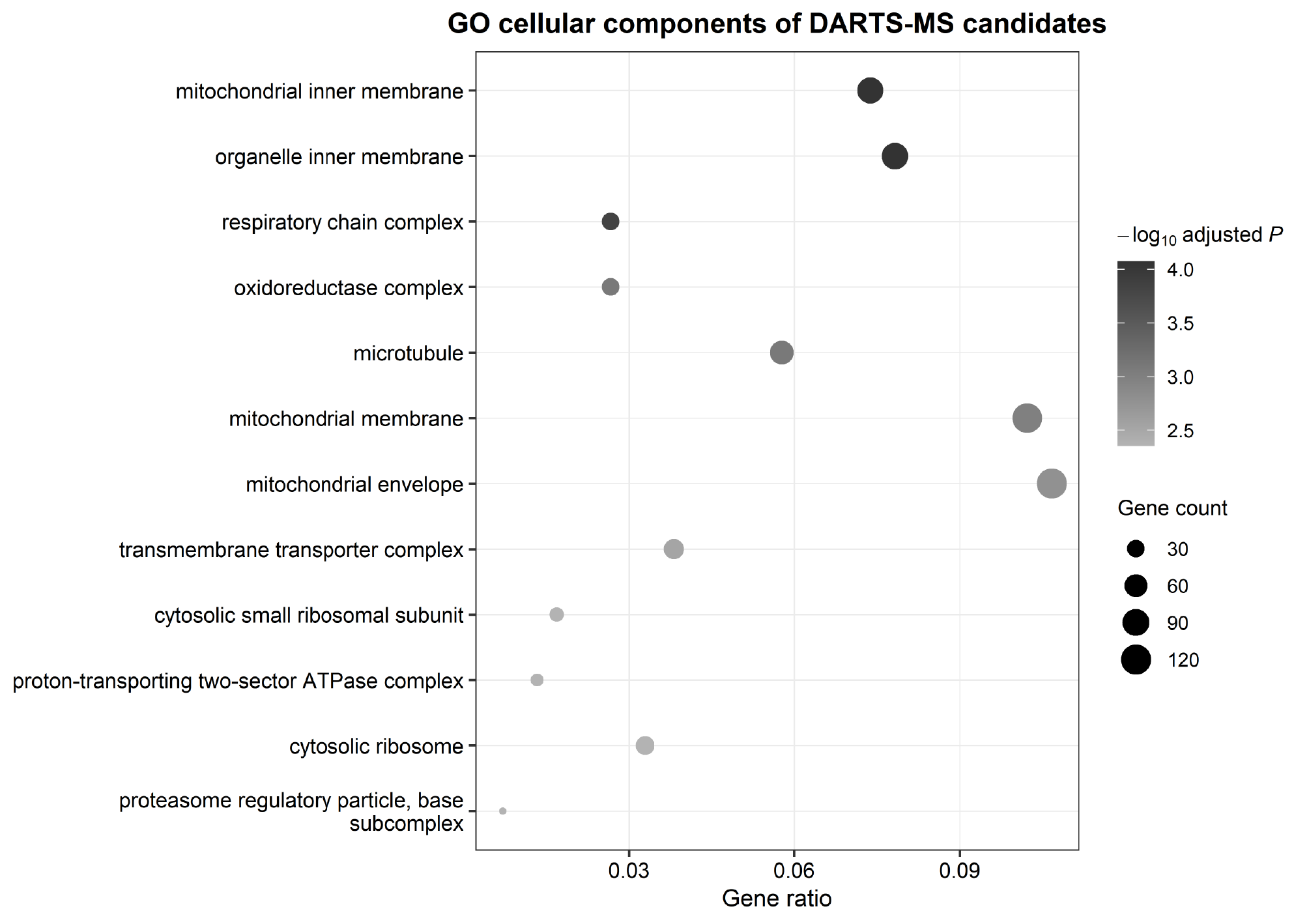

Supplement: Supplementary file 1 [file ijms-27-05815-s001.zip › Supplementary Figures/Supplementary Figure S3.tif]

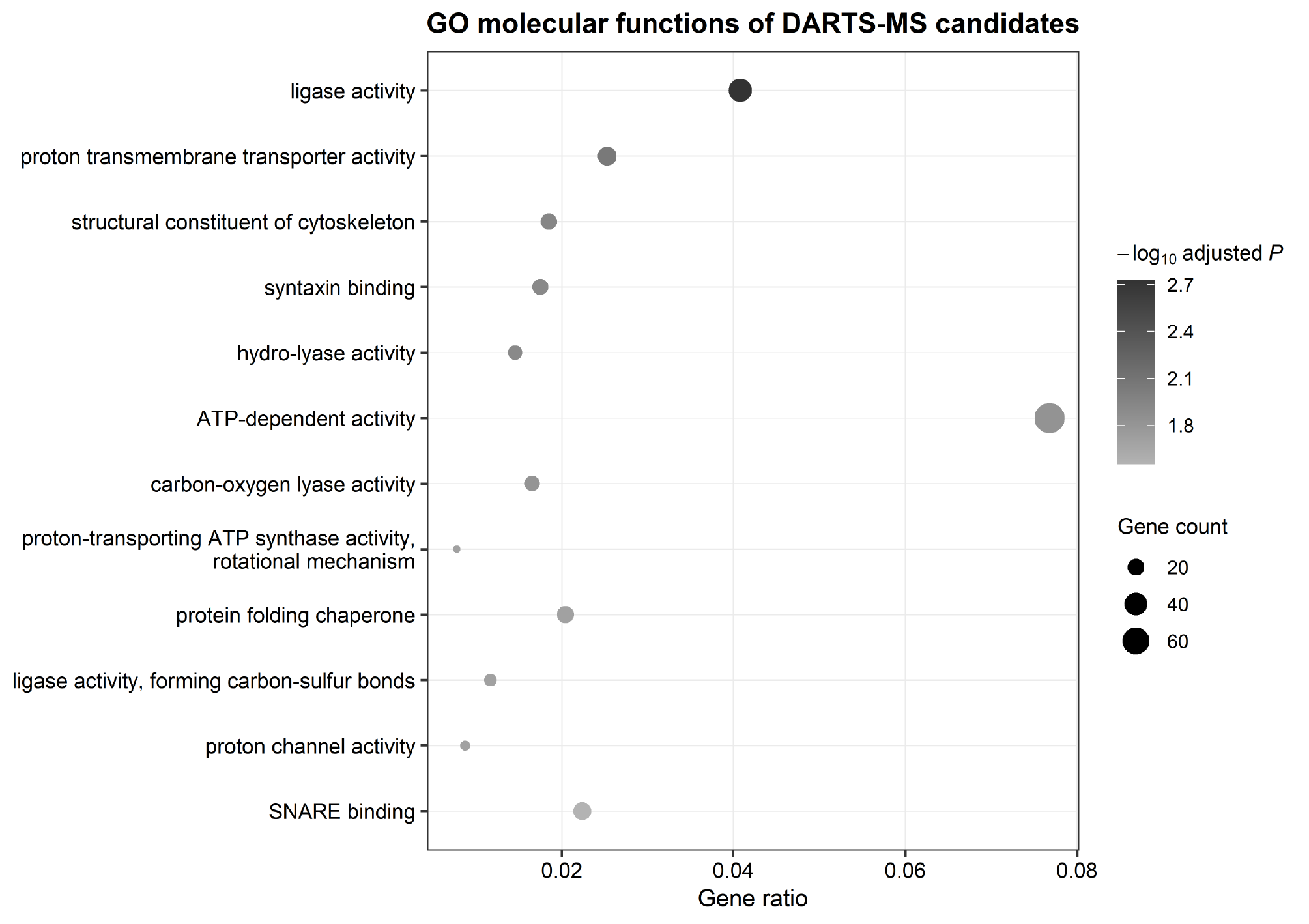

Supplement: Supplementary file 1 [file ijms-27-05815-s001.zip › Supplementary Figures/Supplementary Figure S4.tif]

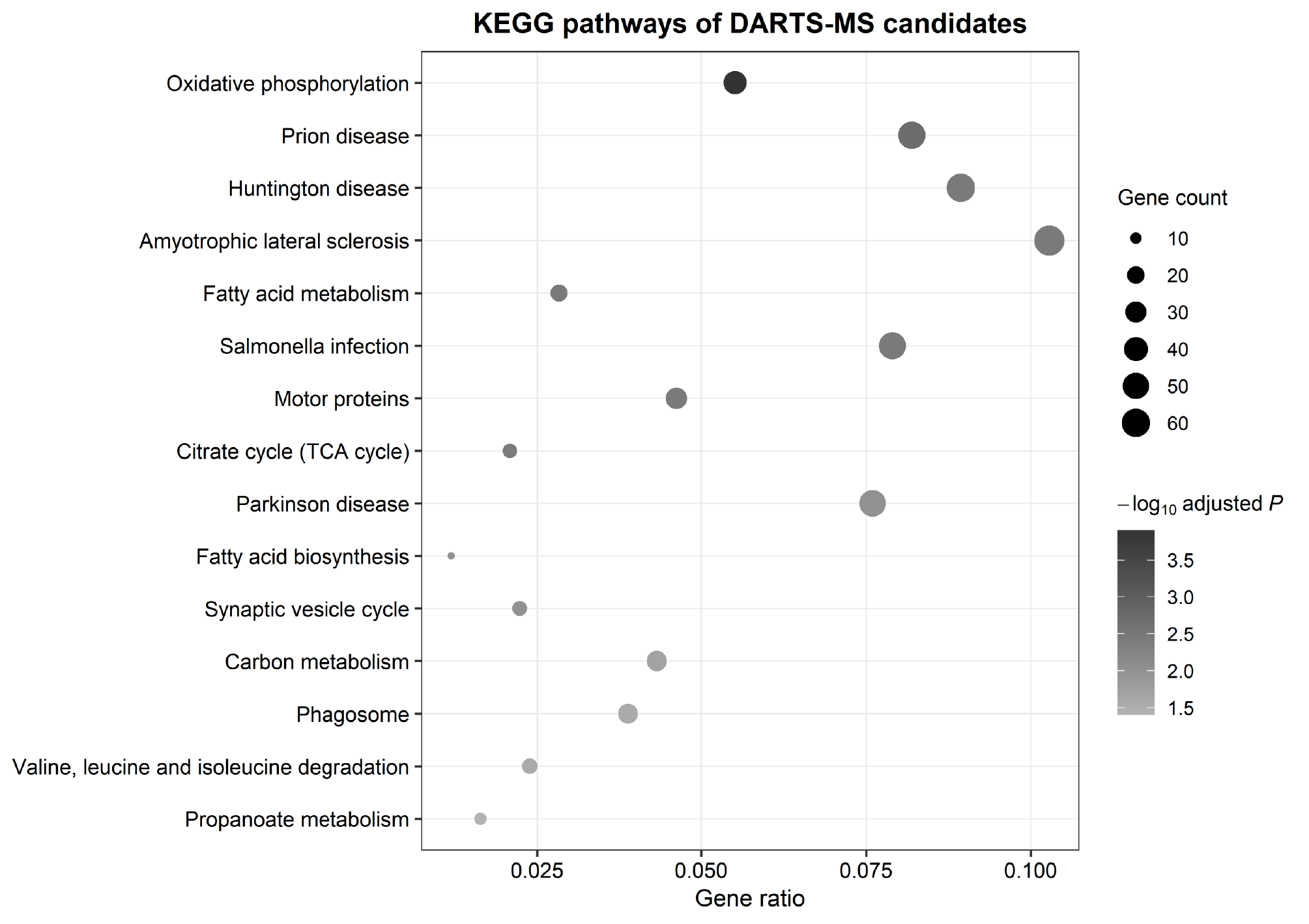

Supplement: Supplementary file 1 [file ijms-27-05815-s001.zip › Supplementary Figures/Supplementary Figure S5.tif]

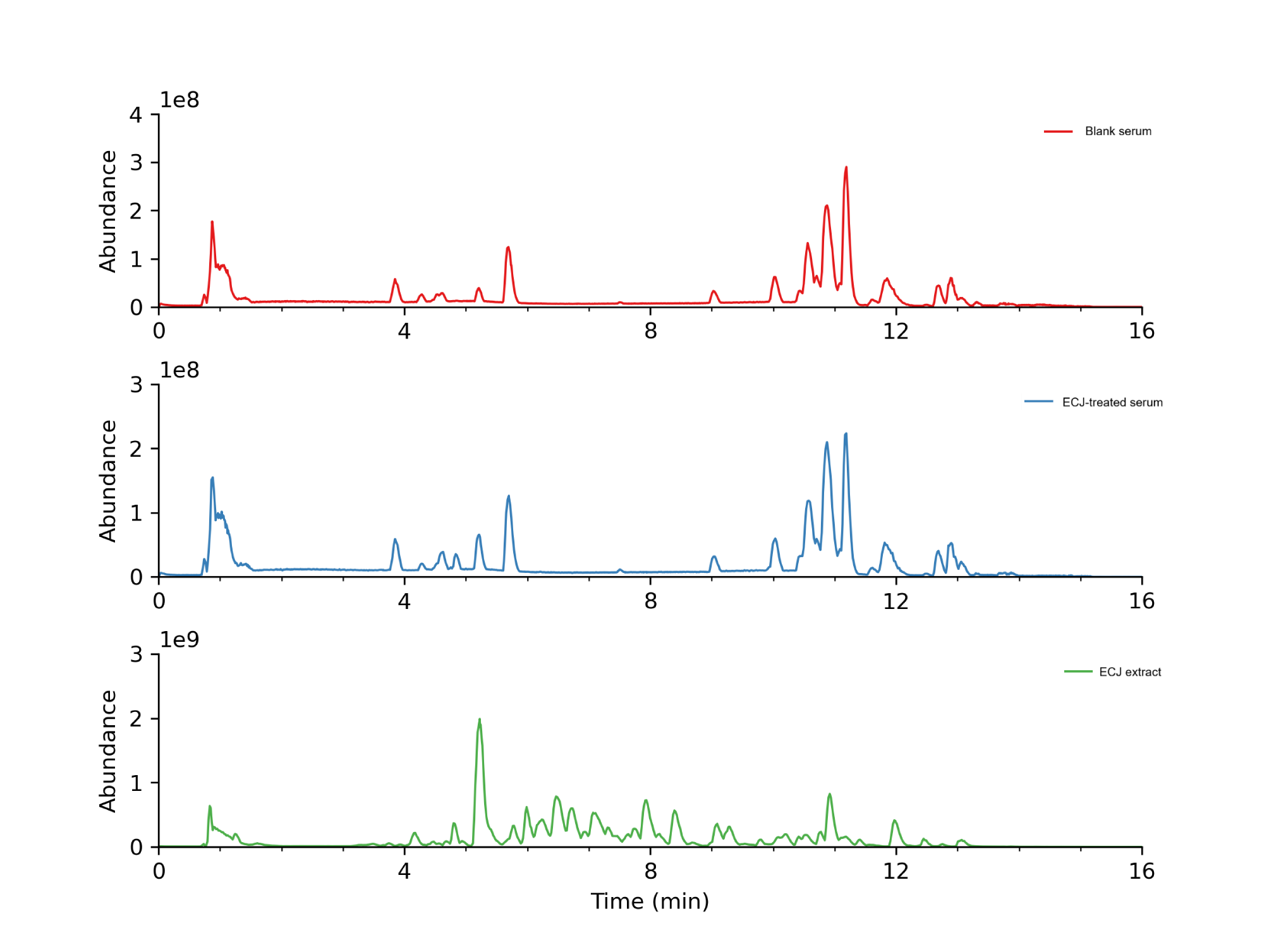

Supplement: Supplementary file 1 [file ijms-27-05815-s001.zip › Supplementary Figures/Supplementary Figure S6.tif]

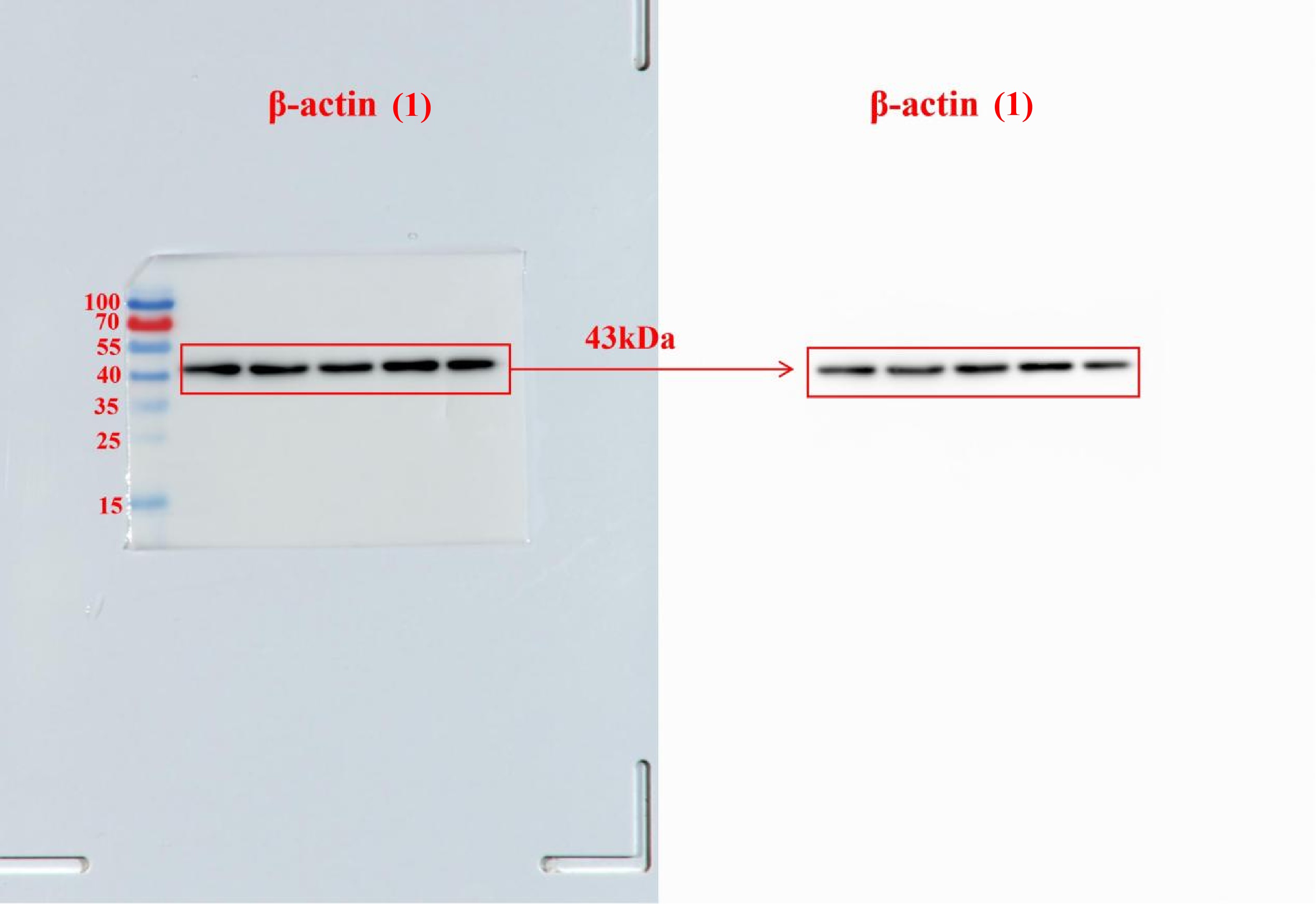

Supplement: Supplementary file 1 [file ijms-27-05815-s001.zip › Supplementary Figures/Supplementary Figure S7/Figure 6A/b-actin(1).jpg]

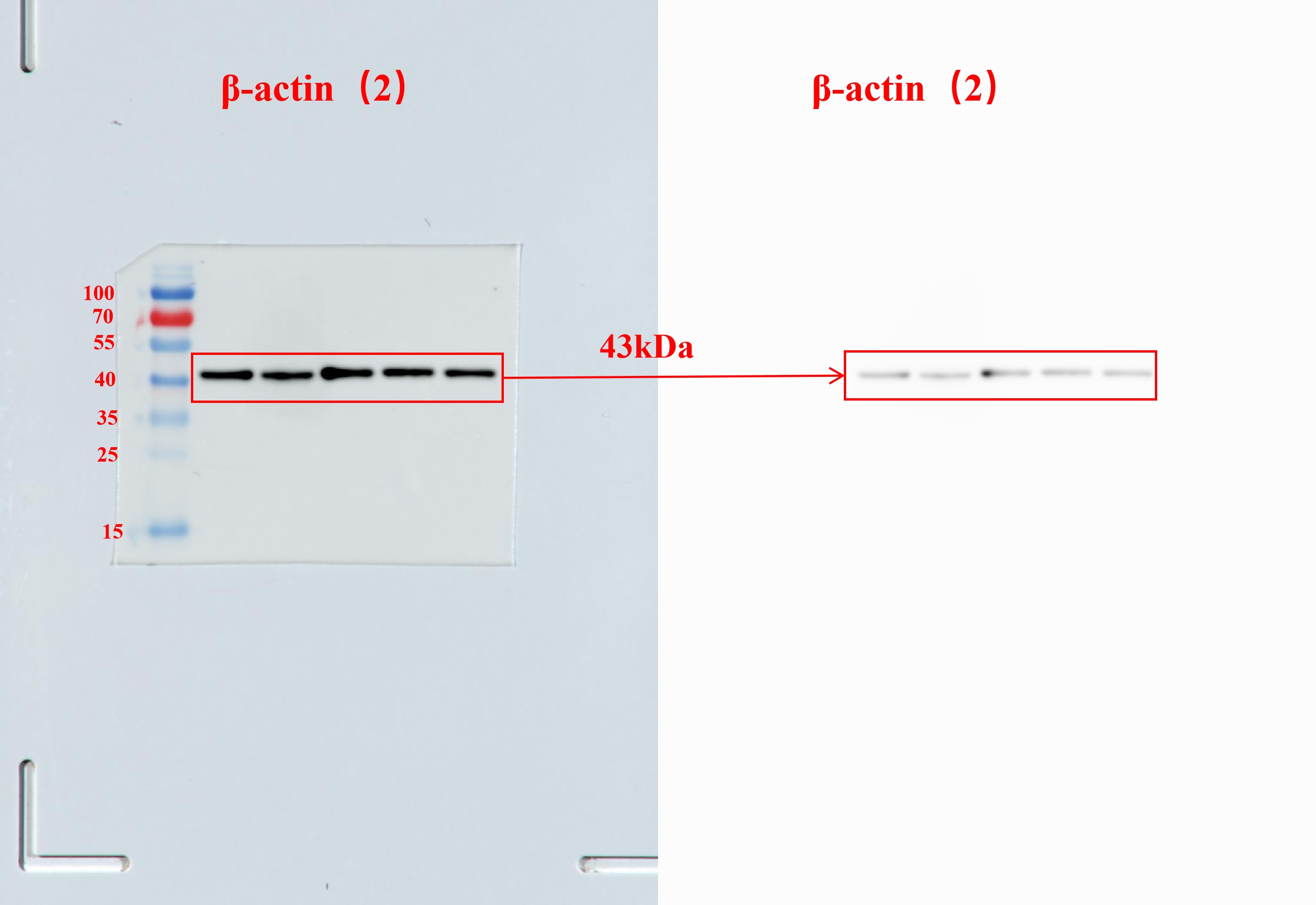

Supplement: Supplementary file 1 [file ijms-27-05815-s001.zip › Supplementary Figures/Supplementary Figure S7/Figure 6A/b-actin(2).jpg]

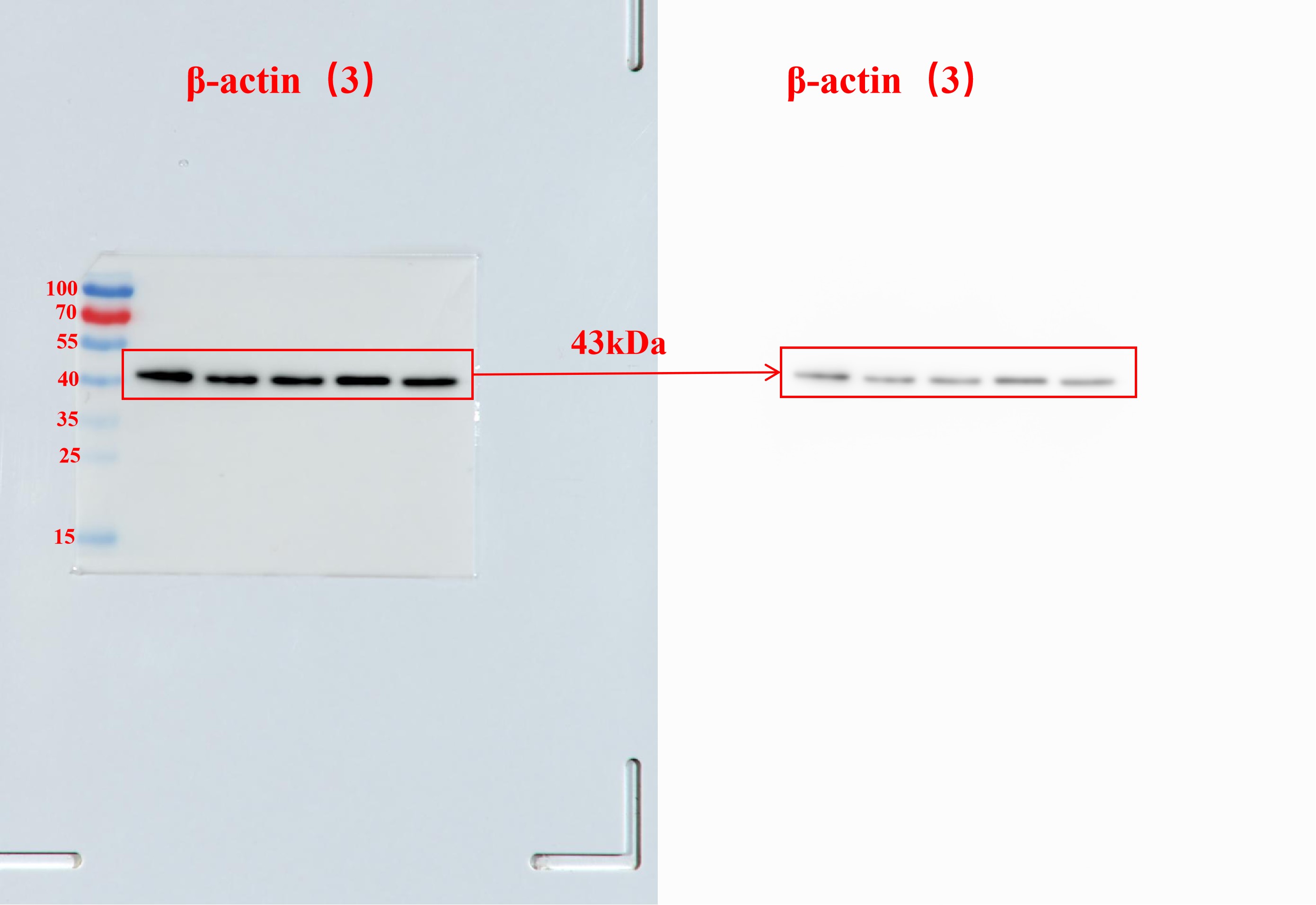

Supplement: Supplementary file 1 [file ijms-27-05815-s001.zip › Supplementary Figures/Supplementary Figure S7/Figure 6A/b-actin(3).jpg]

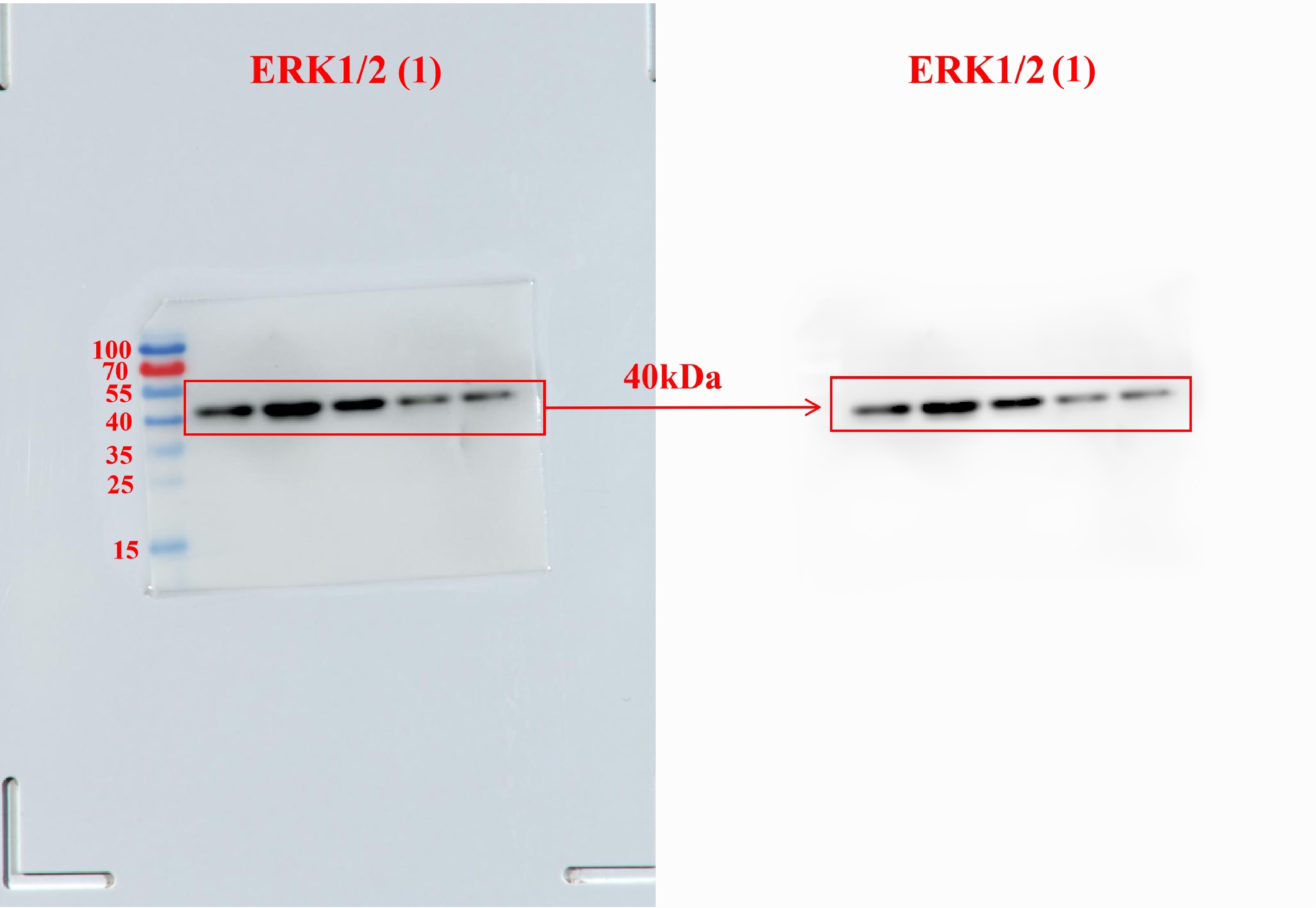

Supplement: Supplementary file 1 [file ijms-27-05815-s001.zip › Supplementary Figures/Supplementary Figure S7/Figure 6A/erk1 2(1).jpg]

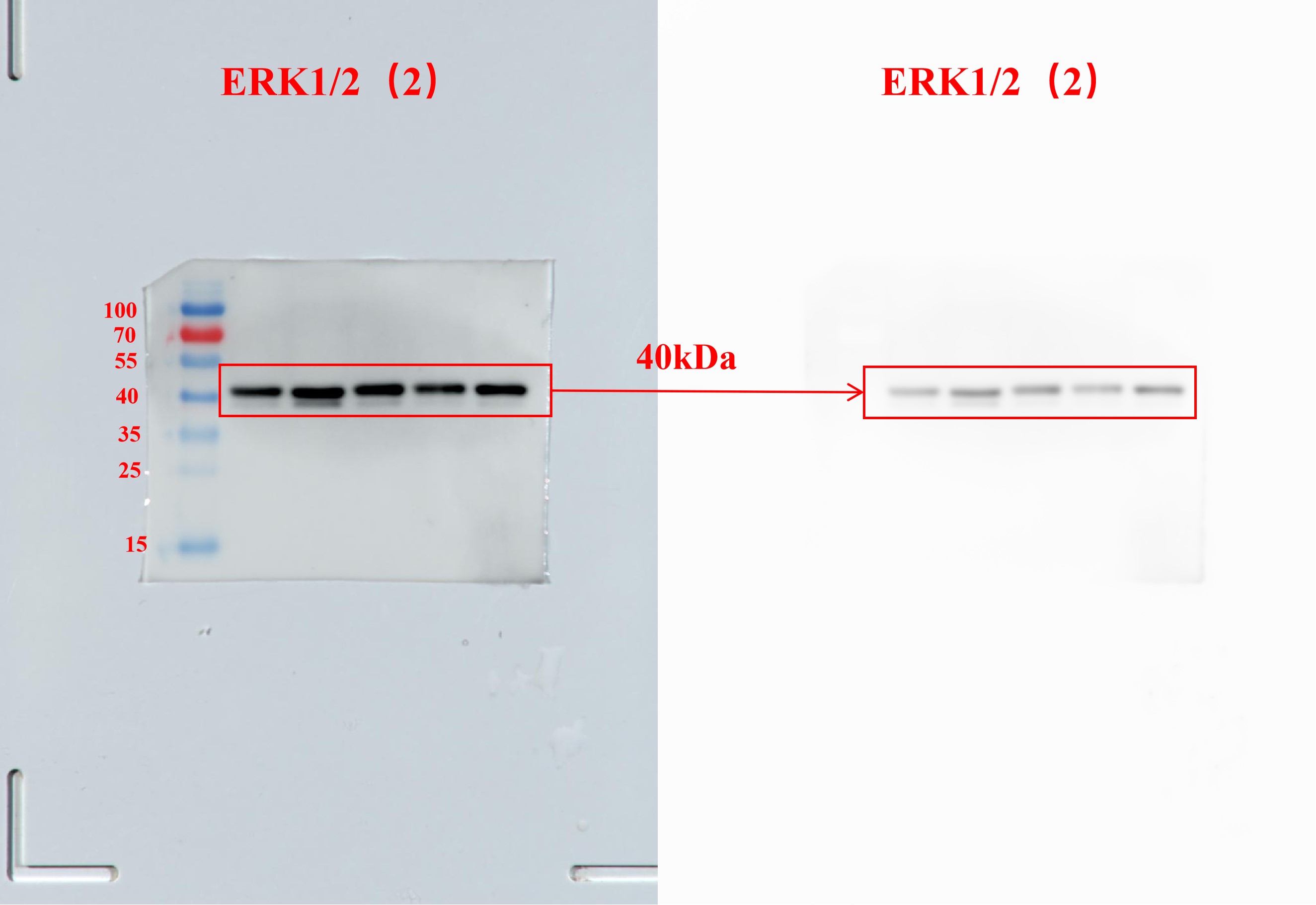

Supplement: Supplementary file 1 [file ijms-27-05815-s001.zip › Supplementary Figures/Supplementary Figure S7/Figure 6A/erk1 2(2).jpg]

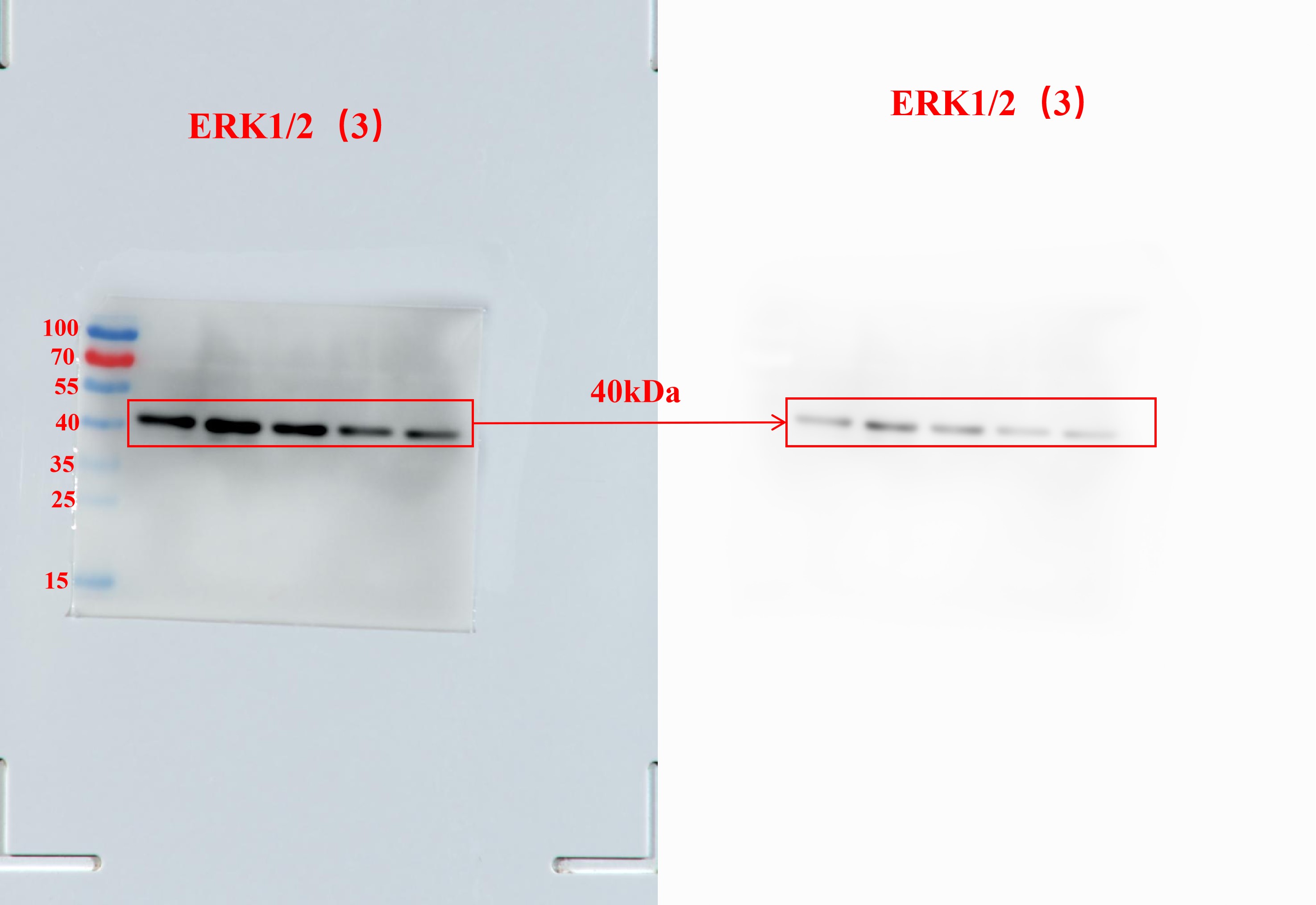

Supplement: Supplementary file 1 [file ijms-27-05815-s001.zip › Supplementary Figures/Supplementary Figure S7/Figure 6A/erk1 2(3).jpg]

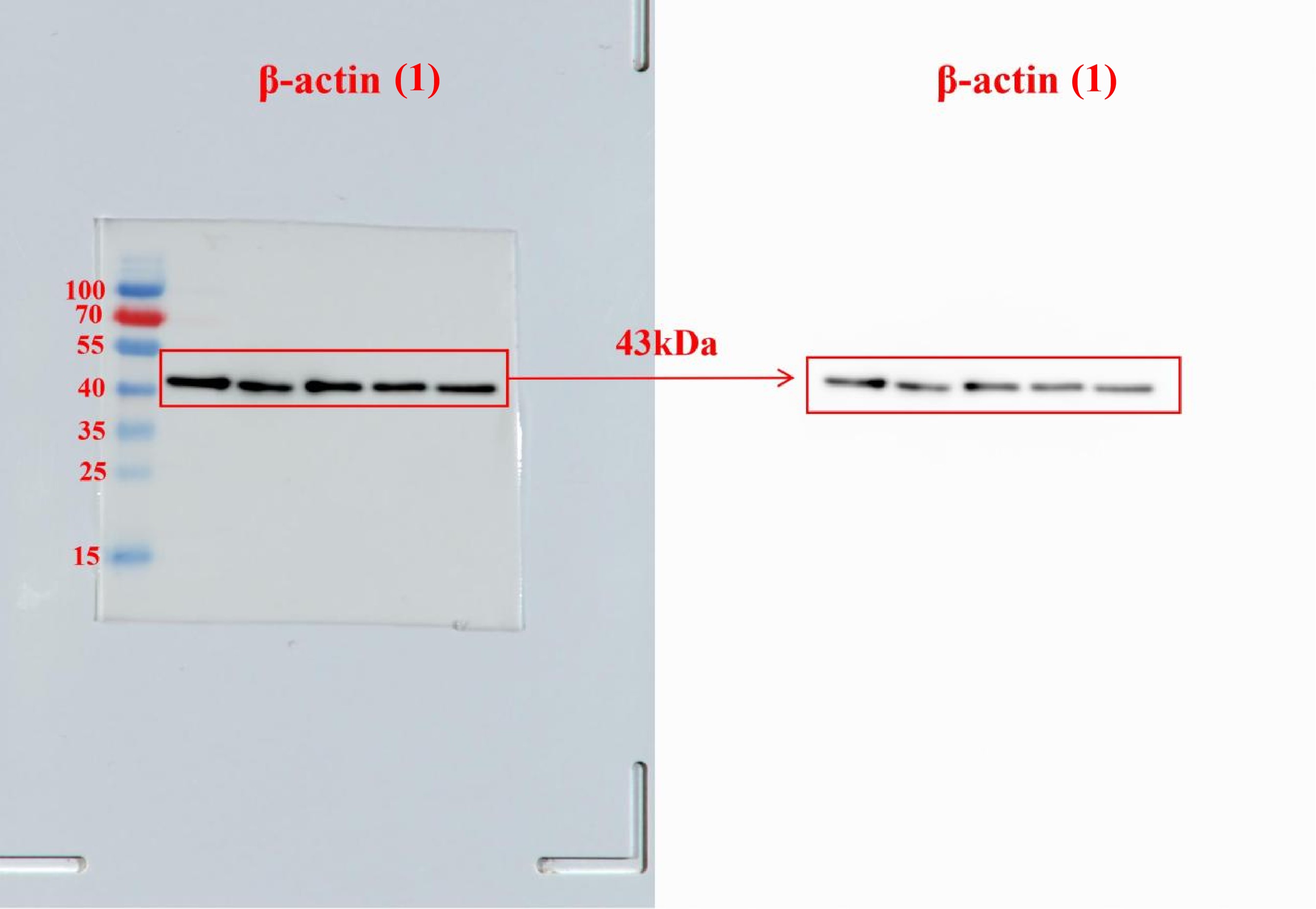

Supplement: Supplementary file 1 [file ijms-27-05815-s001.zip › Supplementary Figures/Supplementary Figure S7/Figure 6B/b-actin1(mek1).jpg]

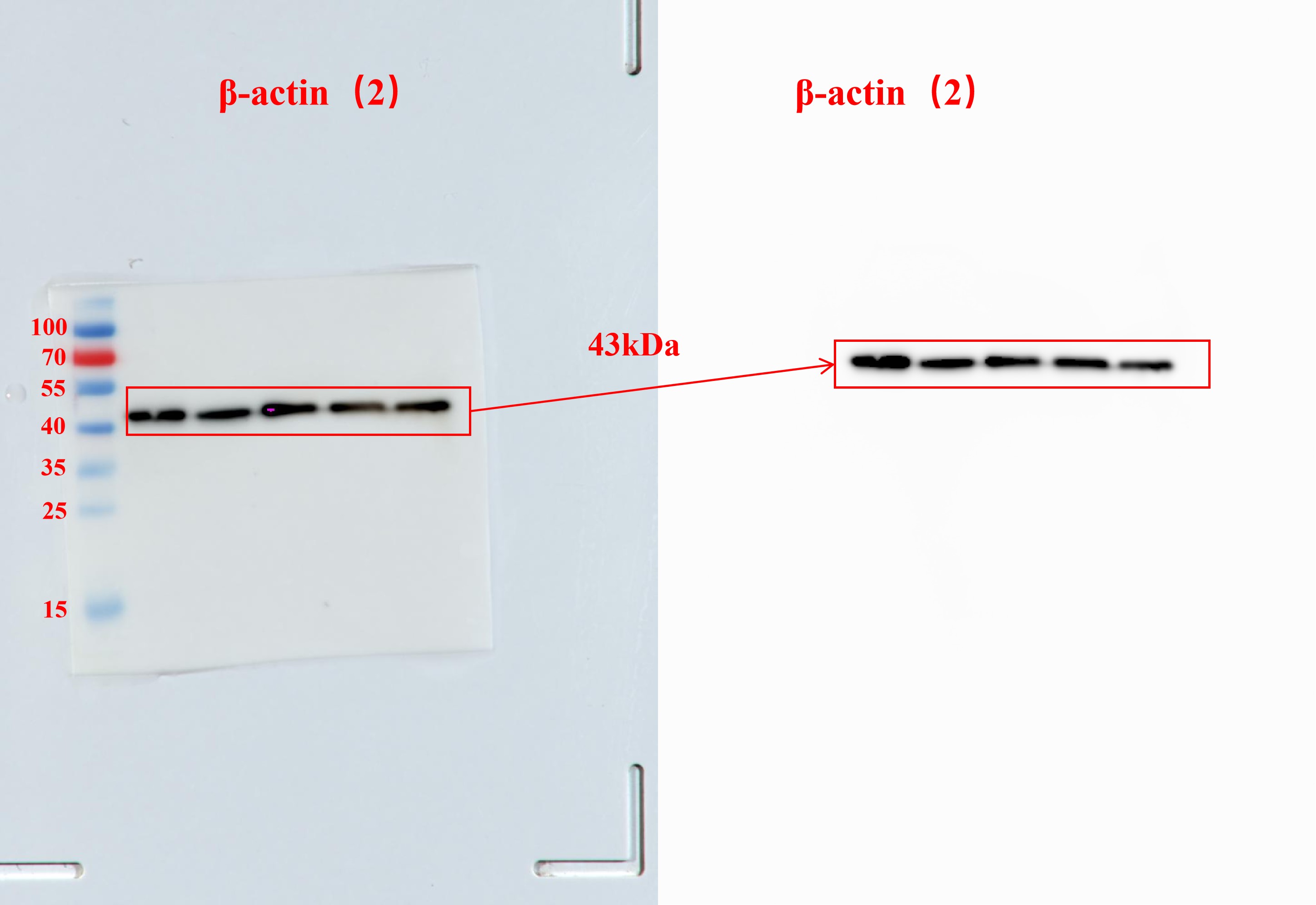

Supplement: Supplementary file 1 [file ijms-27-05815-s001.zip › Supplementary Figures/Supplementary Figure S7/Figure 6B/b-actin2(mek1).jpg]

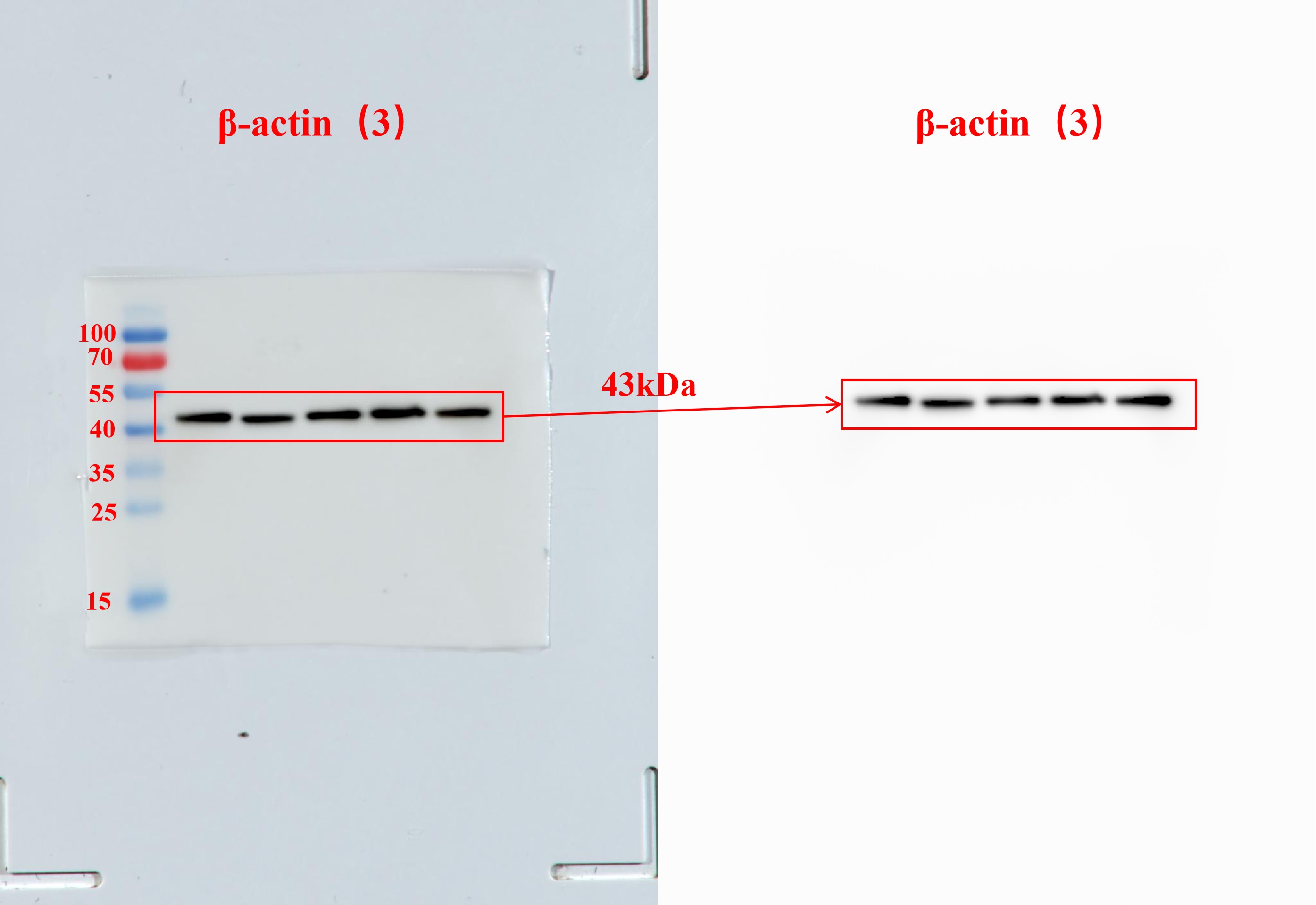

Supplement: Supplementary file 1 [file ijms-27-05815-s001.zip › Supplementary Figures/Supplementary Figure S7/Figure 6B/b-actin3(mek1).jpg]

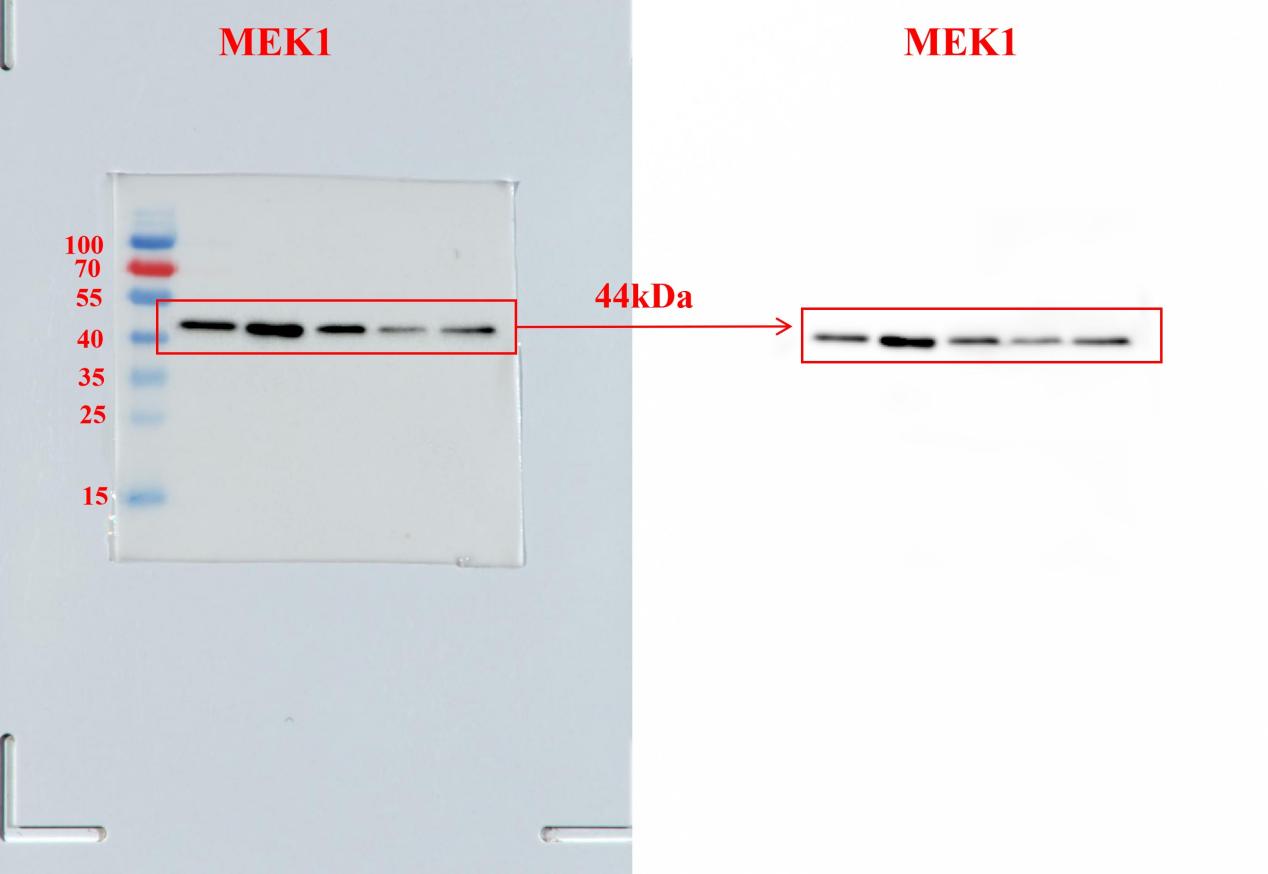

Supplement: Supplementary file 1 [file ijms-27-05815-s001.zip › Supplementary Figures/Supplementary Figure S7/Figure 6B/mek1(1).jpg]

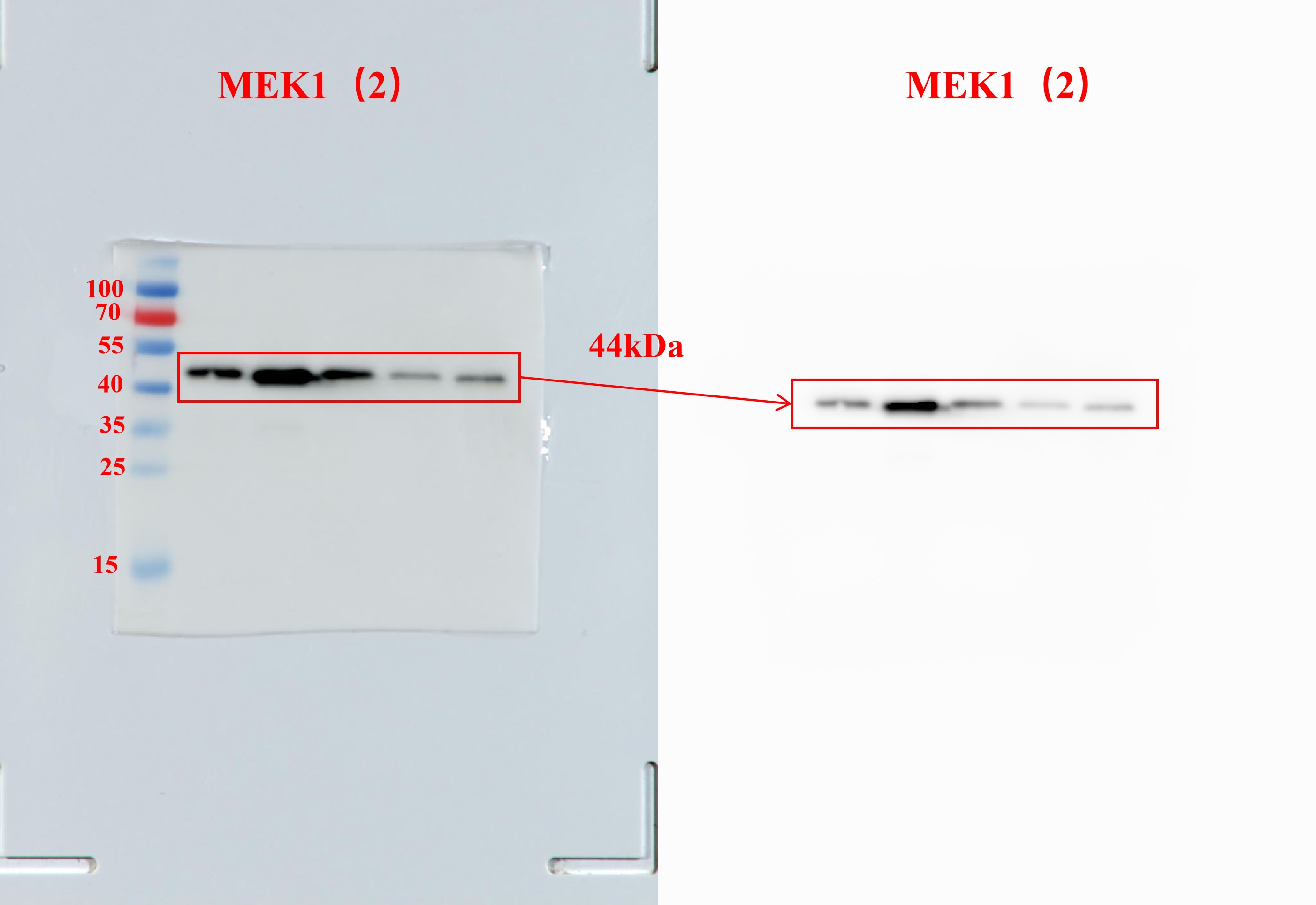

Supplement: Supplementary file 1 [file ijms-27-05815-s001.zip › Supplementary Figures/Supplementary Figure S7/Figure 6B/mek1(2).jpg]

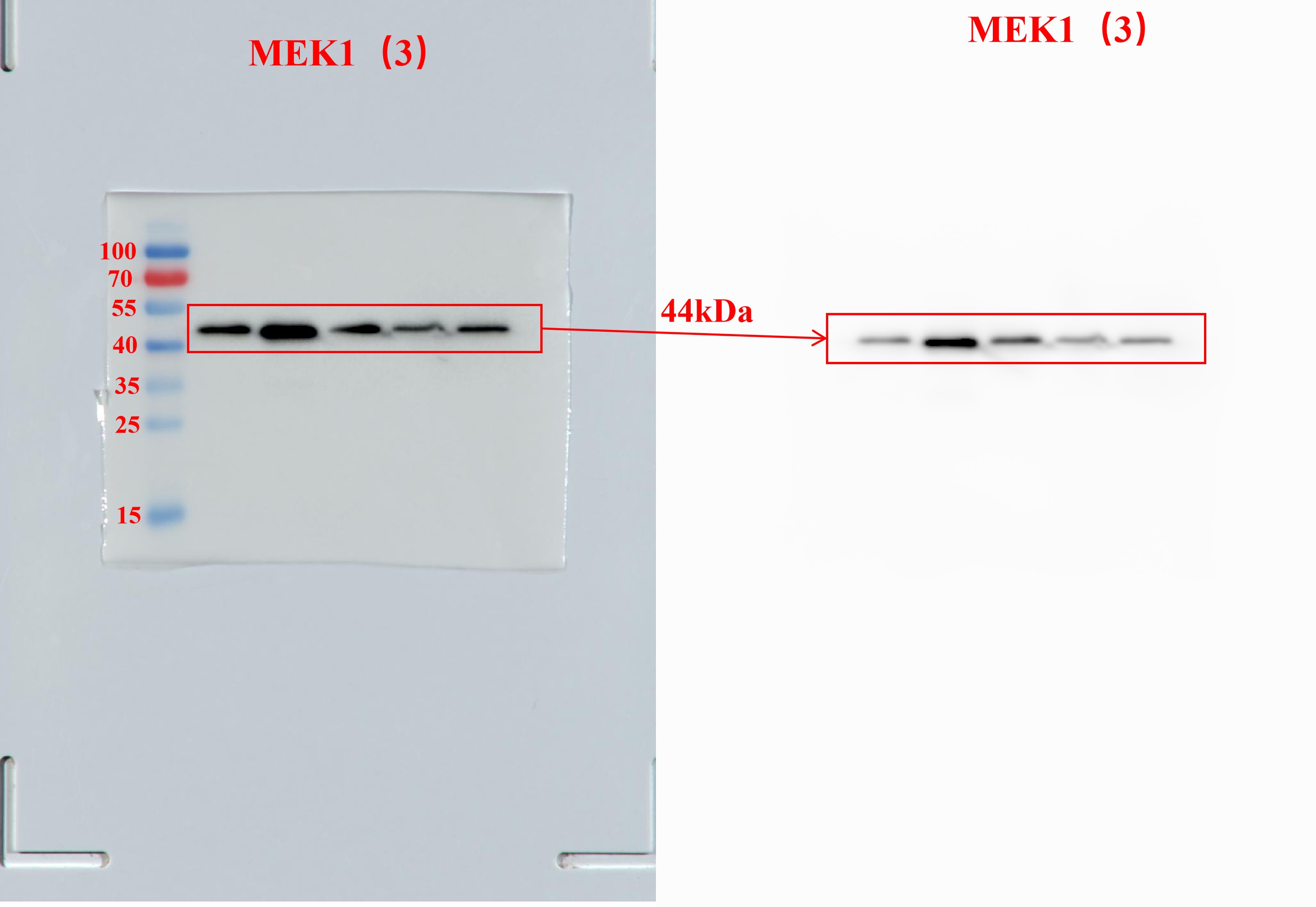

Supplement: Supplementary file 1 [file ijms-27-05815-s001.zip › Supplementary Figures/Supplementary Figure S7/Figure 6B/mek1(3).jpg]

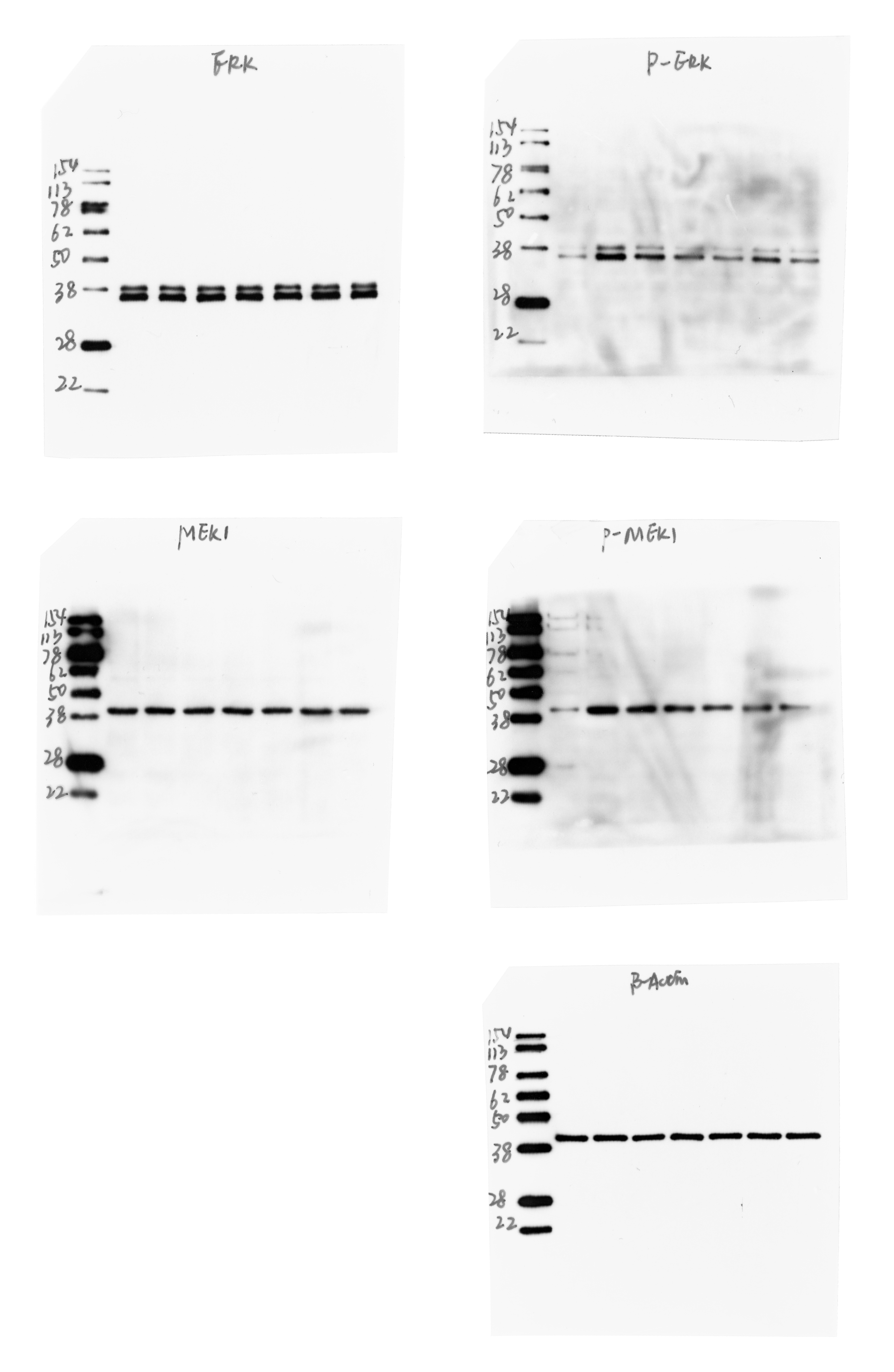

Supplement: Supplementary file 1 [file ijms-27-05815-s001.zip › Supplementary Figures/Supplementary Figure S7/Figure 6C/1-1.tif]

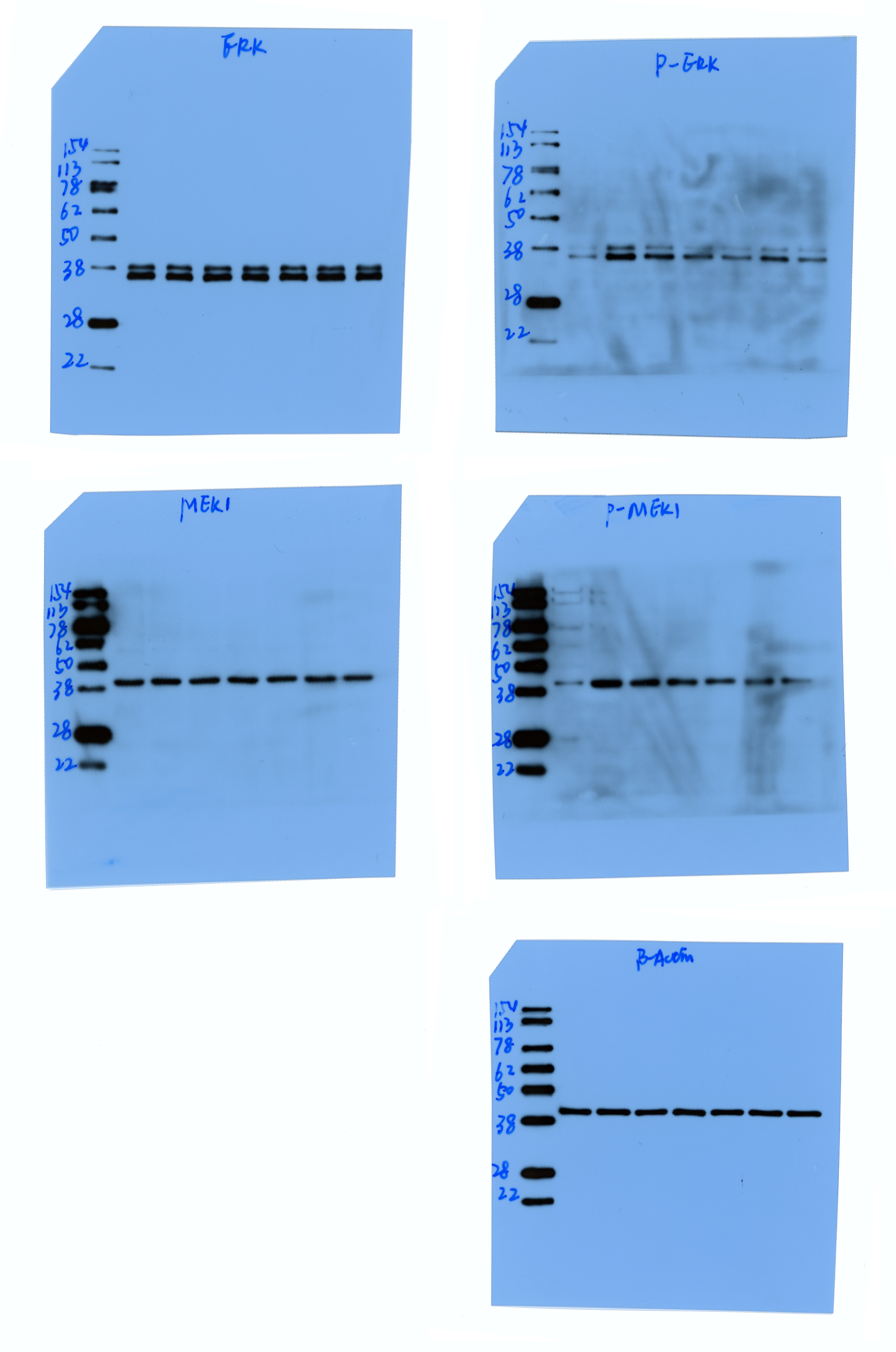

Supplement: Supplementary file 1 [file ijms-27-05815-s001.zip › Supplementary Figures/Supplementary Figure S7/Figure 6C/1-2.tif]

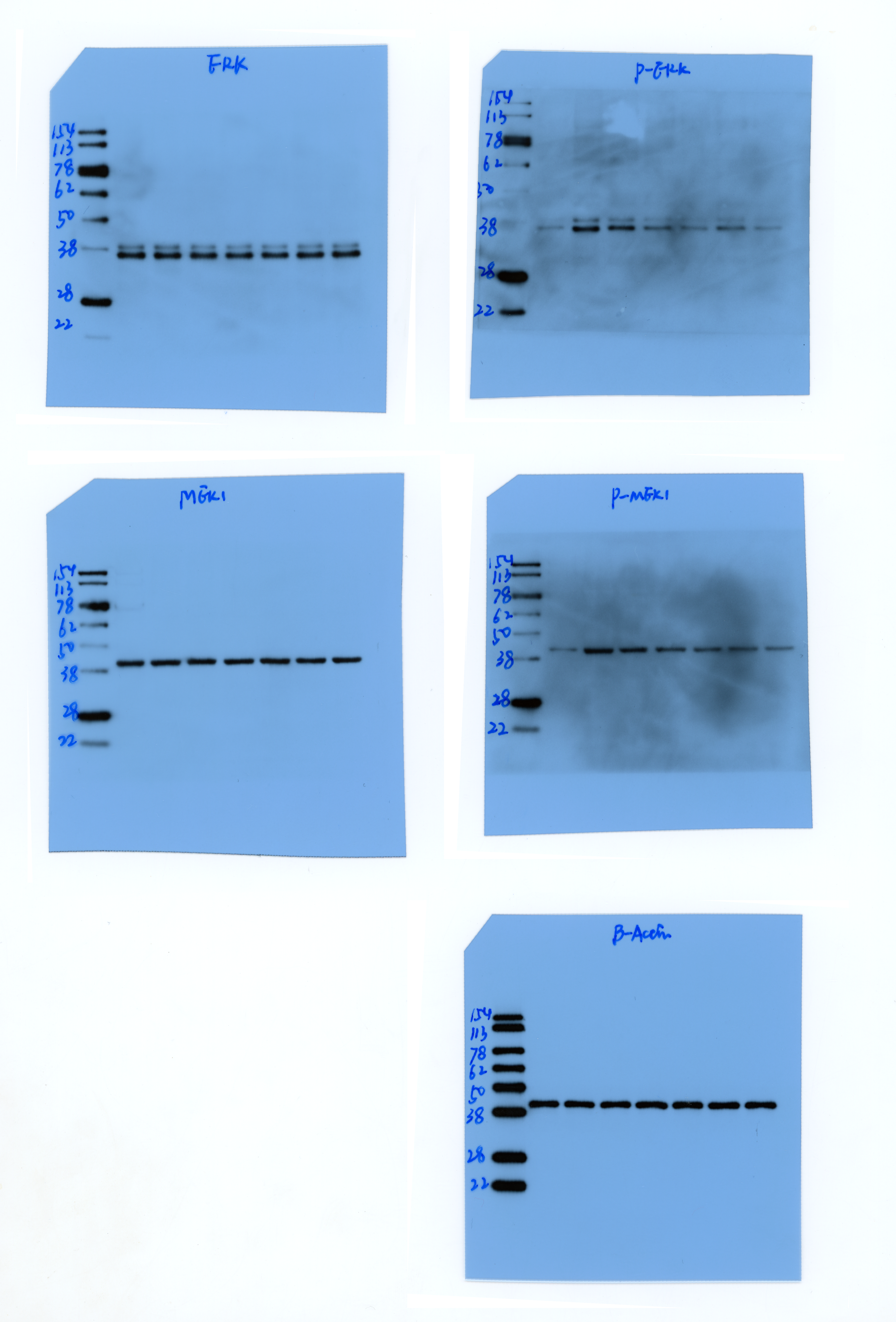

Supplement: Supplementary file 1 [file ijms-27-05815-s001.zip › Supplementary Figures/Supplementary Figure S7/Figure 6C/2-1.tif]

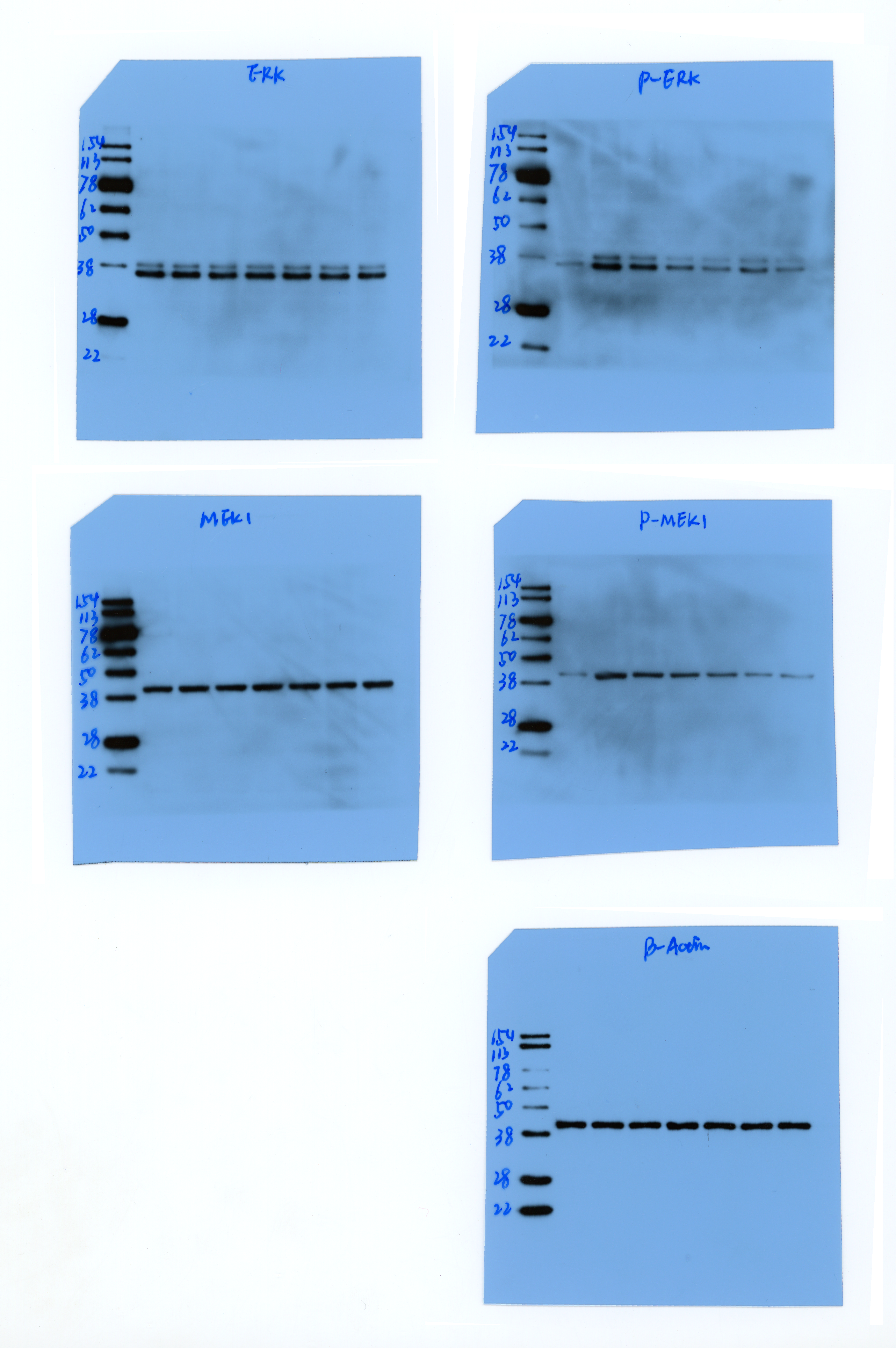

Supplement: Supplementary file 1 [file ijms-27-05815-s001.zip › Supplementary Figures/Supplementary Figure S7/Figure 6C/2-2.tif]

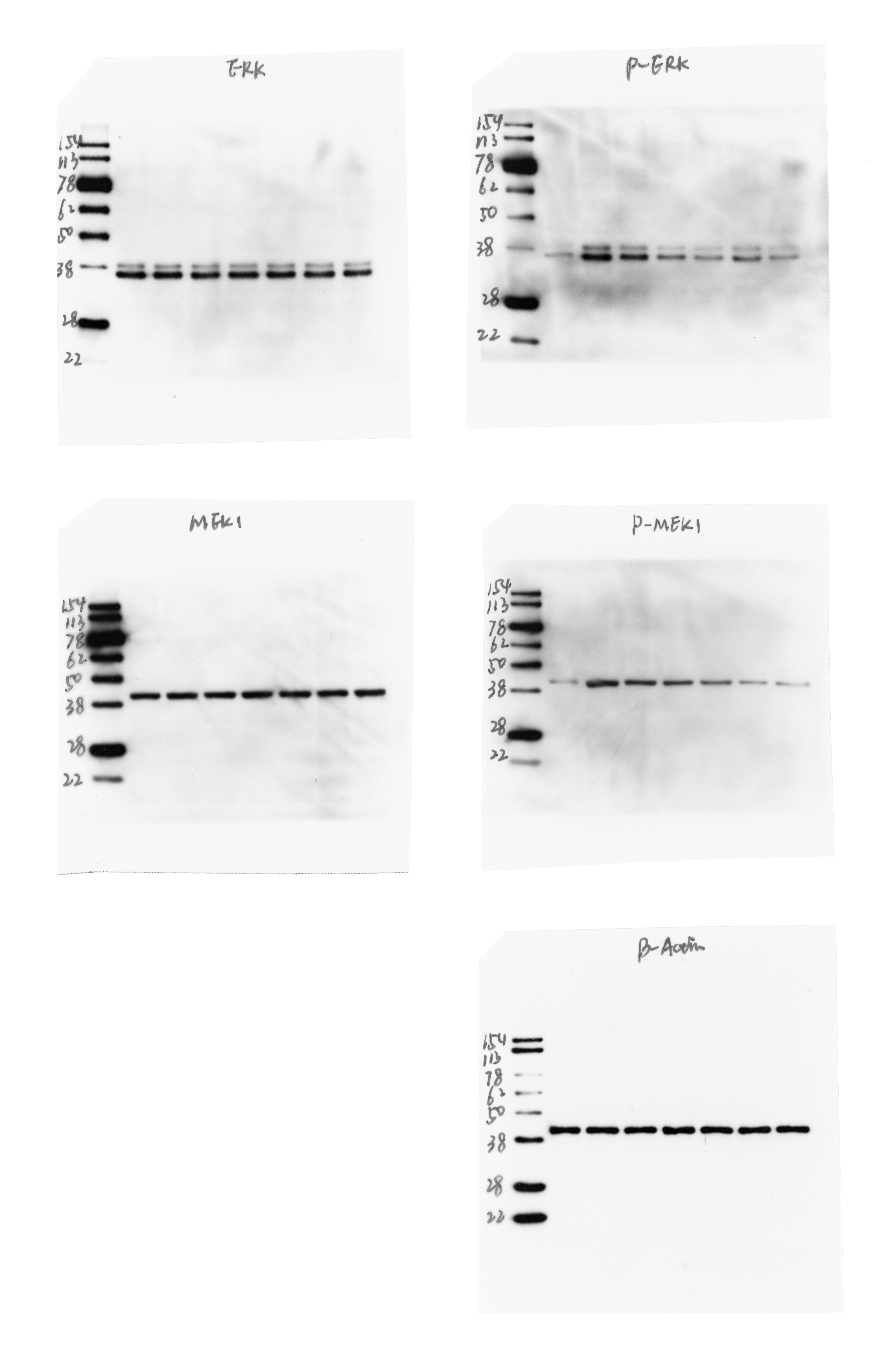

Supplement: Supplementary file 1 [file ijms-27-05815-s001.zip › Supplementary Figures/Supplementary Figure S7/Figure 6C/3-1.tif]

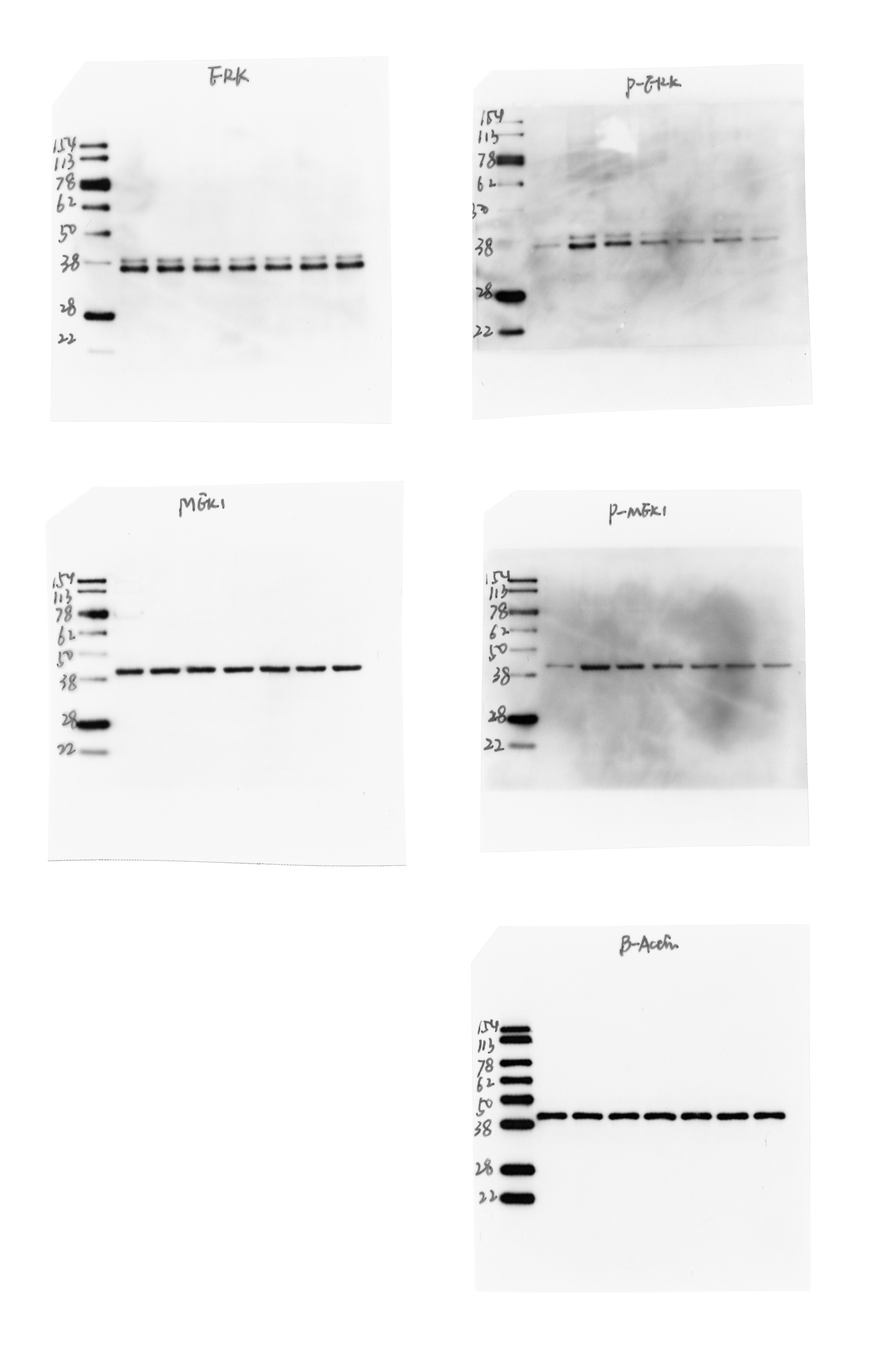

Supplement: Supplementary file 1 [file ijms-27-05815-s001.zip › Supplementary Figures/Supplementary Figure S7/Figure 6C/3-2.tif]
